# Infrared spectroscopy of O^•−^ and OH^−^ in water clusters: evidence for fast interconversion between O^•−^ and OH^•^OH^−^†

**DOI:** 10.1039/c7cp04577h

**Published:** 2017-09-27

**Authors:** Jozef Lengyel, Milan Ončák, Andreas Herburger, Christian van der Linde, Martin K. Beyer

**Affiliations:** Institut für Ionenphysik und Angewandte Physik, Universität Innsbruck, Technikerstraße 25, 6020 Innsbruck, Austria

## Abstract

We present infrared multiple photon dissociation (IRMPD) spectra of (H_2_O)_*n*_O^•−^ and (H_2_O)_*n*_OH^−^ cluster ensembles for $\bar{n}\approx 8$ and 47 in the range of 2400–4000 cm^−1^. Both hydrated ions exhibit the same spectral features, in good agreement with theoretical calculations. Decomposition of the calculated spectra shows that bands originating from H_2_O⋅⋅⋅O^•−^ and H_2_O⋅⋅⋅OH^−^ interactions span almost the whole spectral region of interest. Experimentally, evaporation of OH^•^ is observed to a small extent, which requires interconversion of (H_2_O)_*n*_O^•−^ into (H_2_O)_*n*–1_OH^•^OH^−^, with subsequent H_2_O evaporation preferred over OH^•^ evaporation. The modeling shows that (H_2_O)_*n*_O^•−^ and (H_2_O)_*n*–1_OH•OH^−^ cannot be distinguished by IRMPD spectroscopy.

## Introduction

Many natural processes occur due to interactions of ions in the water environment including proton-transfer dynamics, protein stability, ion transport across cell walls, *etc*., in which the chemistry at the molecular level is still not well understood.^1^ To gain insight into the microscopic interactions between solvent and solute, small cluster ions provide suitable model systems, with mass spectrometry using traps being a useful technique to probe such systems, as the stored cluster ions can be irradiated for rather long times and sensitively analyzed.^2,3^ To approach the structure of the primary hydration shell of ions, infrared multiple photon dissociation (IRMPD) spectroscopy has been successfully applied.^4–11^ The hydroxide ion (OH^−^) is an essential ionic species in aqueous chemistry and its microscopic hydration has been studied in numerous reactivity^12,13^ and spectroscopy^7,14–20^ experiments, mainly elucidating the structure of clusters. Infrared (IR) spectra exhibit broad absorption features that were assigned to rapid fluctuation of isomeric structures.^14^ These observations are in agreement with theoretical calculations,^21^ which found *e.g*. two almost isoenergetic isomers for (H_2_O)_3_OH^−^.

In contrast to the extensive literature on the IR spectroscopy of (H_2_O)_*n*_OH^−^, we have found only one IR spectrum of (H_2_O)_*n*_O^•−^ clusters in the literature, for *n* = 20, reported by Johnson and co-workers.^22^ There is, to the best of our knowledge, no comprehensive study on the (H_2_O)_*n*_O^•−^ cluster ions available in the literature. One of the reasons could be the instability of the O^•−^ ion in water clusters and formation of OH^•^OH^−^ in reaction with water. *Ab initio* calculations on small clusters^23^ have shown that O^•−^ and OH^•^OH^−^ have similar stability. In bulk water, O^•−^ is predicted to be the more stable isomer,^24,25^ in contrast to previous experiments that favored OH^•^OH^−^.^26^ *Ab initio* molecular dynamics of the reaction between N_2_O and sodium doped water clusters, Na(H_2_O)_15_, yielded O^•−^ formation mediated by a hydrated electron,^27^ with a subsequent reaction to produce the OH^•^OH^−^ pair on the picosecond timescale.

In this work, we combine Fourier transform ion cyclotron resonance (FT-ICR) mass spectrometry and IRMPD spectroscopy with DFT calculations to study the nature of the interaction between O^•^/OH^−^ anions and water molecules. First, we discuss details of the hydrated ions structure by interpretation of the IRMPD spectra; second, we investigate the conversion of O^•−^ into OH^•^ OH^−^, which is evidenced *via* the evaporation of OH^•^.

## Experimental and theoretical methods

The IRMPD experiments were performed using a 4.7 T Fourier-transform ion cyclotron resonance (FT-ICR) mass spectrometer^28,29^ equipped with a tunable optical parametric oscillator (OPO). The reactant ions, (H_2_O)_*n*_^−^, were generated in a home-built external source^28^ by laser vaporization of a solid zinc target and supersonic expansion of the hot plasma in a helium/water gas pulse.^30,31^ The anionic water clusters were carried from the ion source into the ICR cell, where they further reacted with N_2_O at room temperature to yield both (H_2_O)_*n*_O^•−^ and (H_2_O)_*n*_OH^−^ cluster ions.^27,32,33^ (H_2_O)_*n*_O^•−^ and (H_2_O)_*n*_OH^−^ clusters were then irradiated at specific frequencies with tunable IR laser radiation in the range of 2400–4000 cm^−1^, which is generated by a commercial OPO system (EKSPLA NT277). The laser radiation was introduced through a window at the rear end of the vacuum system into the ICR cell. The diode pumped Q-switched laser and OPO system, operated at 1000 Hz repetition rate, provides pulse energies of ~90–190 μJ. The irradiation induced dissociation of the precursor ions by the sequential loss of water molecules. The absolute photodissociation cross section was calculated from these data.

(H_2_O)_*n*_O^•−^ and (H_2_O)_*n*_OH^−^ clusters are initially produced in two broad size distributions with mean cluster sizes of $\bar{n}\approx 8$ and $\bar{n}\approx 47.$ The environment is at room temperature, while the clusters have an internal temperature of about 100 K, as established in calorimetric experiments by von Issendorff and co-workers.^34^ At each studied wavelength, a mass spectrum was taken after irradiating all clusters simultaneously, followed by recording a mass spectrum without laser irradiation. Following the approach described by Prell, O’Brien and Williams for size-selected water cluster,^35^ this allowed to quantify the contribution of blackbody infrared radiative dissociation (BIRD)^36–41^ on cluster dissociation. These measurements also minimize the influence of fluctuations of the cluster size distribution.

Since we work with an ensemble of clusters over an extended size range, we cannot employ directly the equations presented by Williams and co-workers.^35^ Building on the differential equations derived for nanocalorimetry of cluster ensembles,^29^ we can describe the change in the average cluster size d*N* induced by IR laser irradiation as well as BIRD, eqn (1):

(1) $$\text{d}N=-\left[k_{\text{f}}(N-N_{0})+\frac{\sigma Ep}{A\Delta E_{\text{vap}}}\right]\text{d}t$$

The first term describes BIRD of a water cluster ensemble^29^ with *k*_f_ describing the linear dependence of the unimolecular BIRD rate on the cluster size, which is measured independently. *N* is the average cluster size and *N*_0_ accounts for the contribution of the ionic core to the BIRD absorption cross sections. The second term describes evaporation of water molecules induced by IR laser irradiation with the photodissociation cross section *σ*, the pulse energy *E* per area *A*, the pulse repetition rate *p*, *i.e*. number of laser pulses per unit time, and the energy required to evaporate a single water molecule from the water cluster Δ*E*_vap_, which is 43.3 ± 3.1 kJ mol^−1^.^34,42^ It is assumed that the energy of the absorbed laser photons is fully available for water evaporation. Since radiative cooling cannot be completely ruled out, this constitutes a source of error in the derived cross section.

For the practical evaluation of the absolute photodissociation cross section from the IRPD spectra, we use eqn (2), which was derived by integration of eqn (1) with and without the laser irradiation term. Δ*N* is the difference in the average cluster size without and with IR laser irradiation, but in both cases subject to BIRD during the irradiation time *t* in the ICR cell. In this analysis, the cross section *σ* is assumed to be independent of cluster size, which is an approximation.

(2) $$\sigma =\frac{A\Delta N\Delta E_{\text{vap}}}{Ep}\frac{k_{\text{f}}}{1–{\text{e}}^{-k_{\text{f}}t}}$$

The parameter *k*_f_ is determined by fitting a BIRD kinetics of a cluster ensemble recorded at the same temperature. Since all clusters absorb laser photons, as evidenced by the significant shift in cluster size, we assume that complete overlap between the laser beam and the ion cloud in the ICR cell was achieved. However, the inhomogeneity in the laser beam profile and the only indirect measurement of the photon flux inside the ICR cell might affect the absolute values by up to a factor of two.

IR spectra and reaction energies were modelled at the B3LYP/TZVP level of theory, with Grimme’s D2 dispersion correction.^43^ For each cluster type, (H_2_O)_*n*_O^•−^, (H_2_O)_*n*–1_OH^•^OH^−^, (H_2_O)_*n*_OH^−^, and for *n* = 6–11, 15 different isomers were optimized from various starting structures (*i.e*. 270 isomers in total). All found structures represent local minima and their energies lie within 30 kJ mol^−1^ with respect to the most stable structure found. This sampling approach was picked as molecular dynamics had the tendency to remain in one part of the potential energy surface, not sampling efficiently the conformational space. Vibrations were calculated within the harmonic approximation and analyzed according to their type by calculating projection onto normal modes. For this purpose, an O–H bond is defined when *r*(O–H) < 1.3 Å, a hydrogen bond between O_don_H⋯O_acc_ when *r*(O_acc_–H) < 2.5 Å and α(O_don_–H–O_acc_) > 120°. Vibrations were scaled by 0.96 to reproduce the position of the free OH vibration (~3700 cm^−1^). The final spectra were modeled as an arithmetic average of IR spectra of individual isomers. The spectra were broadened by Lorentzian functions with the full width at half maximum of 40 cm^−1^; this value was selected so that the final spectrum has neither a too pronounced structure nor it becomes too blurred. At the same time, the free OH vibration is well distinguishable in the total spectrum. Zero point energy corrections are included in all reported reaction energies. All calculations were performed in the Gaussian 09 program package,^44^ the respective local minima are provided in the ESI.†

## Results and discussion

**Fig. 1:**
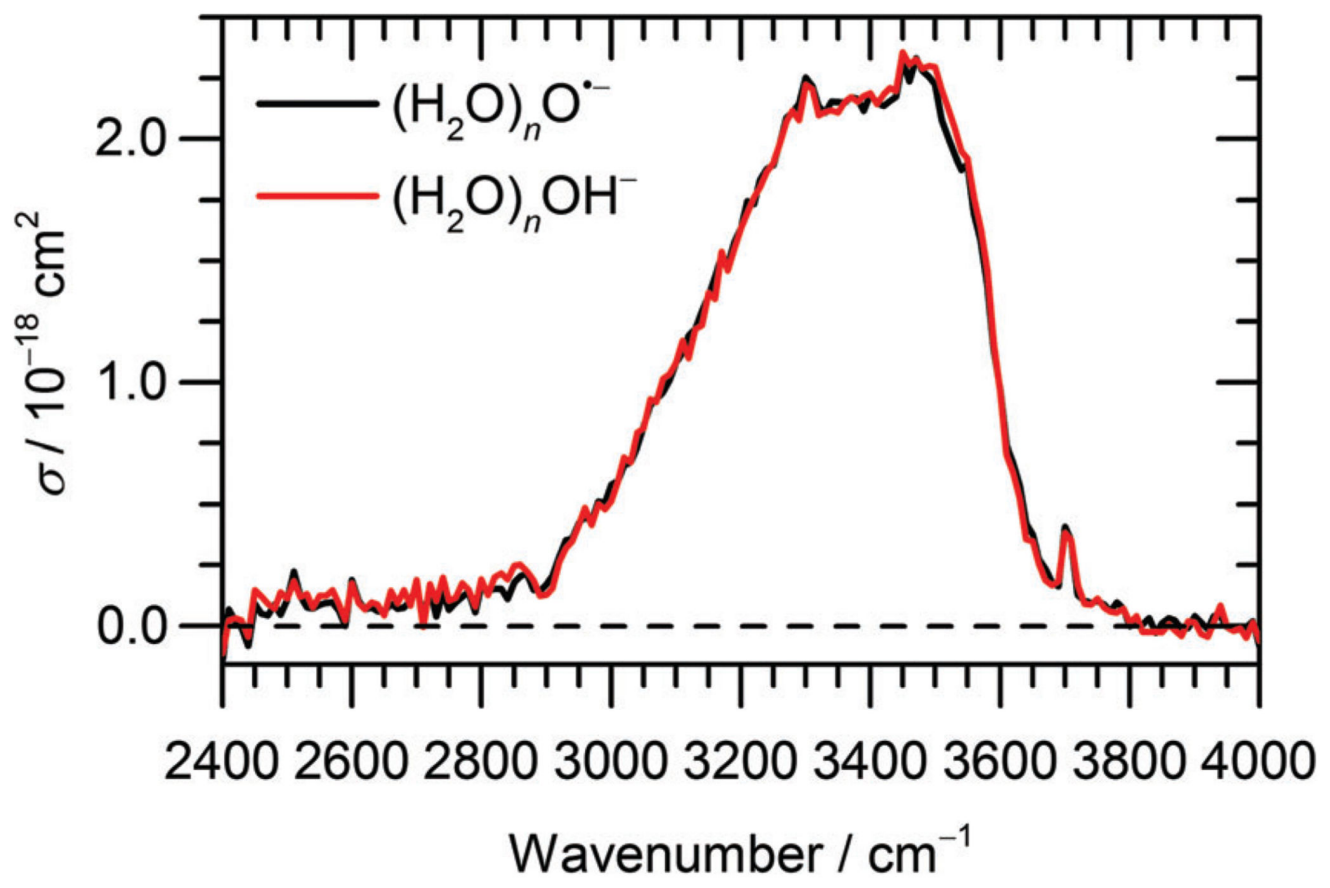
Experimental IRMPD spectrum of (H_2_O)_*n*_O^•−^ and (H_2_O)_*n*_OH^−^ cluster ions, $\bar{n}\approx 47,$ measured in the range of 2400–4000 cm^−1^.

**Fig. 2:**
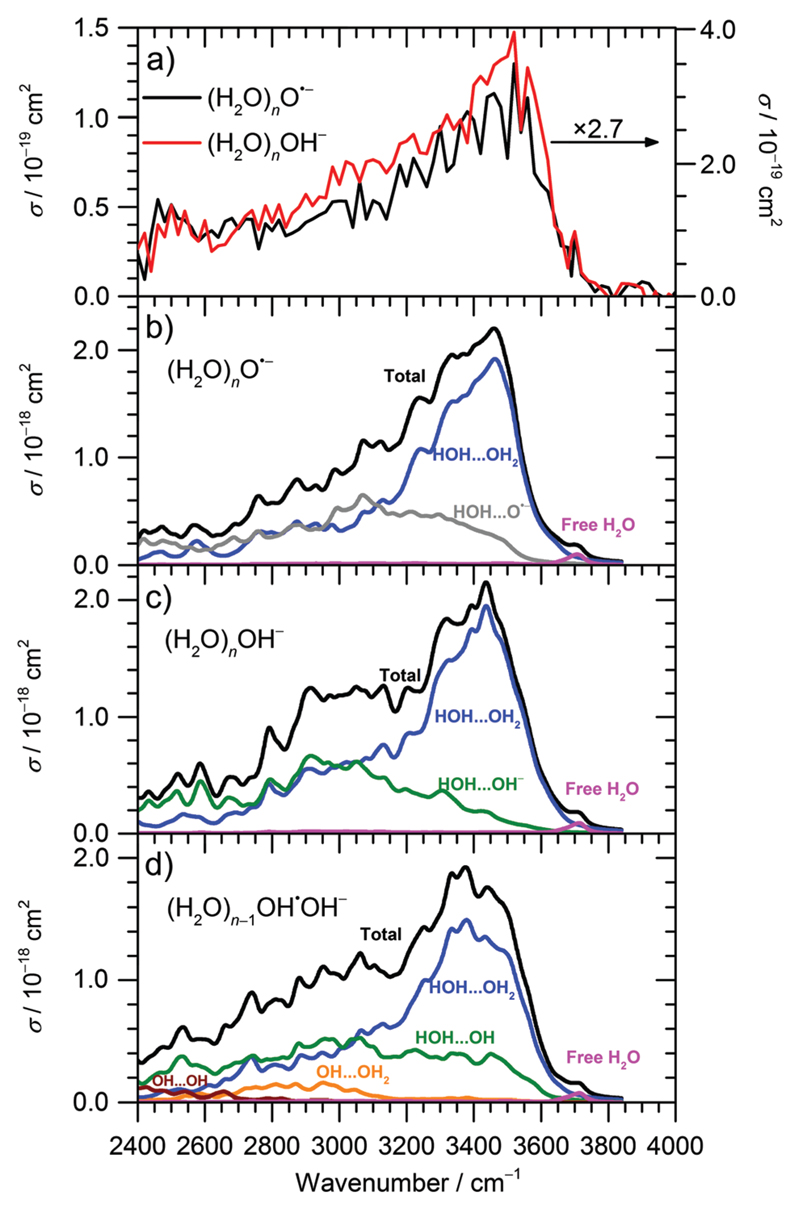
(a) Experimental IRMPD spectrum of (H_2_O)_*n*_O^•−^, (H_2_O)_*n*_OH^−^ clusters for $\bar{n}\approx 8;$ (b–d) calculated IR spectra of low-energy isomers of (H_2_O)_*n*_O^•−^, (H_2_O)_*n*_OH^−^, and (H_2_O)_*n*–1_OH^•^OH^−^ cluster ions for *n* = 6–11 (90 isomers are included per spectrum). Calculated at the B3LYP+D2/TZVP level of theory, spectra were broadened by Lorentzian functions with the full width at half maximum of 40 cm^−1^, vibrational frequencies were scaled by the factor of 0.96; each spectrum was decomposed into contributions originating from various interactions (see Methods). For comparison, the vibrational frequency of free OH^−^ in the gas phase was calculated to be 3492 cm^−1^ (after scaling).

The IRMPD spectra of (H_2_O)_*n*_O^•−^ and (H_2_O)_*n*_OH^−^ ions are shown in Fig. 1 and 2a for $\bar{n}\approx 47$ and $\bar{n}\approx 8,$ respectively. Clearly, the IRMPD spectra of both hydrated ions are almost identical, but change significantly with cluster size.

From previous studies of neutral water clusters,^45–47^ we can assign the broad absorption feature (3000–3600 cm^−1^) with a maximum around 3450 cm^−1^ to the O–H stretching vibrations of water molecules that are hydrogen bonded in the cluster. A sharp and, at least for the larger clusters, well-separated free O–H stretch near 3700 cm^−1^ is attributed to water molecules on the cluster surface. The measured absorption bands are very similar to those for amorphous water clusters^46,47^ that were described in detail by theoretical calculations of Buch and co-workers.^45^

For the current work, the 2450–2900 cm^−1^ region is of main interest. Here, the observed absorption signal behaves qualitatively similar for both larger and smaller clusters, a weak absorption without pronounced structure is observed. A previous study on small (H_2_O)_*n*_OH^−^ clusters^15^ suggested that this signal originates from the hydrogen bonds of water molecules to the anion. For larger clusters, water–water interaction in the vicinity of the ion might also contribute. If one assumes that the chromophore responsible for this feature, *i.e*. the hydrated ion, is the same regardless of cluster size, also the absolute cross sections should be the same. This is obviously not the case, the cross sections averaged over 2450–2900 cm^−1^ are a factor of 2.7 larger for the $\bar{n}\approx 47$ clusters. However, since the absolute cross sections depend sensitively on the overlap of the laser beam with the ion cloud, we cannot rule out that the $\bar{n}\approx 8$ clusters were irradiated with significantly fewer photons. A possible reason for this could be the storage period of 8 s before irradiation started to allow the clusters to shrink to the desired size due to BIRD. Since the signal was relatively intense, space charge effects over this period lead to spatial expansion of the ion cloud, which in turn would lead to reduced overlap with the laser beam. We therefore evaluated the total oscillator strength of the O–H stretching modes in the two cluster distributions, which is proportional to the integral of the absorption spectrum from 2400 to 4000 cm^−1^. We obtain a factor of 15.2 for the ratio of the two integrals, while the size ratio is 47/8 = 5.9. However, if we scale the $\bar{n}\approx 8$ spectrum by the factor of 2.7 obtained above, the ratio of the two integrals reduces to 5.6, close to the expected 47/8 ratio. We therefore conclude that the intensity of the $\bar{n}\approx 8$ spectrum in Fig. 2a is too low. If we multiply the intensities in Fig. 2a by 2.7, see scale bar on the right, the theoretical values in Fig. 2b–d are still a factor of 6.2 too high. Given the approximations that enter the calculation of IR absorption cross sections in quantum chemistry, however, this agreement seems acceptable. Another potential source of error in the experimental cross sections is the neglect of infrared emission following absorption of an IR laser photon. In eqn (1), we assume that the absorbed laser photons contribute fully to water evaporation, which is probably an over-simplification. IR emission will become more and more relevant with the decreasing cluster size, which could also explain the discrepancy between the $\bar{n}\approx 47$ and $\bar{n}\approx 8$ absorption cross sections. However, for a precise comparison between experiment and theory and for a fully reliable determination of experimental absorption cross sections, detailed master equation modeling of BIRD and IR laser irradiation will be required.

Theoretical calculations of IR spectra (Fig. 2b and c) provide further evidence that the IR intensity in the 2450–2900 cm^−1^ region is induced by the presence of O^•−^ or OH^−^ anions. Both calculated spectra have similar shape and reproduce well the experimental data. Spectra of O^•−^ clusters exhibit a slightly lower relative intensity in the 2400–2700 cm^−1^ region compared to OH^−^ clusters; this difference, however, might be due to the limitations of the modeling approach.

Decomposition of the calculated IR spectra of hydrated O^•−^ and OH^−^ ions into various interaction types shows that the water–water interaction fingerprint is located in the broad region starting already at about 2400–2600 cm^−1^, with a strong increase in intensity for higher wavenumbers. However, we can expect that the intensity in the 2400–2900 cm^−1^ region is induced mainly by the presence of the anion; neutral water clusters do not show any absorptions in this wavelength region, indicating that these contributions come from water–ion interactions as well as water–water interactions in the vicinity of the anion.^45,48^ For larger clusters, the intensity in the 2400–2900 cm^−1^ region will thus diminish relative to the intensity in the 3000–3600 cm^−1^ region, as discussed above. The region comprising interaction of water molecules with the solvated ions (OH^−^, O^•−^) spreads among the whole 2400–3600 cm^−1^ region, with about similar intensity. For (H_2_O)_*n*_OH^−^, *n* = 1–5, the broad weak continuum absorption over the whole spectrum, 2450–3800 cm^−1^, was interpreted by Niedner-Schatteburg and co-workers14 as a rapid interconversion of cluster isomers. This is in agreement with our calculations as single isomers exhibit sharp peaks in the 2400–3000 cm^−1^ region and spectral broadening is gained by averaging. Note also that the IR spectra in Fig. 2 were calculated using a local minima approach. Therefore, the spectrum broadening arises due to the presence of different isomers; the absorption below 3000 cm^−1^ can be explained without invoking a mobile proton as suggested by Johnson and co-workers for protonated water clusters,^49^ but this does not rule out its presence in the current experiments.

Thus, the absorption intensity in the 2400–2800 cm^−1^ region can be attributed mainly to the signal induced by the presence of O^•−^/OH^−^ ion in the cluster while both ion-water and water–water interactions are present for higher frequencies. Finally, the free O–H vibration is located at about 3700 cm^−1^; this characteristic peak was used to choose the scaling factor of the calculated vibrational spectra (see Methods section).

Besides the O^•−^ isomer considered above, the (H_2_O)_*n*_O^•−^ clusters can also undergo the proton transfer reaction (3), forming the (H_2_O)_*n*–1_OH^•^OH^−^ structure which might be formed in two forms, with OH moieties forming contact or solvent separated pairs. In our calculations, these two forms have almost the same occurrence.

(3) $${\left({\text{H}}_{2}\text{O}\right)}_{n}{\text{O}}^{•-}\to {\left({\text{H}}_{2}\text{O}\right)}_{n-1}\text{O}{\text{H}}^{•}\text{O}{\text{H}}^{-}$$

Interestingly, our calculations predict that isomers containing the OH^•^OH^−^ motif exhibit IR spectra that are very similar to both O^•−^ and OH^−^ core ions (Fig. 2d), with a broad H_2_O⋯OH peak and OH^•^⋯OH^−^ interaction located at 2400–2700 cm^−1^. Thus, we cannot distinguish hydrated O^•−^ from OH^•^OH^−^ *via* IRMPD spectroscopy.

In agreement with an *ab initio* study on small (H_2_O)_*n*_O^•−^ and (H_2_O)_*n*–1_OH^•^OH^−^ clusters, *n* = 1–5,^23^ our calculations at the B3LYP+D2/TZVP level show that for *n* = 6–11, both isomers lie close in energy (within 7 kJ mol^−1^, see Table 1). However, while calculations on small clusters found that (H_2_O)_*n*_O^•−^ are generally preferred, no clear trend is seen in our case.

The presence of the OH^•^OH^−^ isomer in the current experiment can be deduced directly from OH^•^ evaporation. If the OH^•^OH^−^ motif is present, loss of the OH^•^ radical, reaction (4), competes with loss of H_2_O, reaction (5).

(4) $${\left({\text{H}}_{2}\text{O}\right)}_{n}\text{O}{\text{H}}^{•}\text{O}{\text{H}}^{-}\to {\left({\text{H}}_{2}\text{O}\right)}_{n}\text{O}{\text{H}}^{-}+\text{O}{\text{H}}^{•}$$

(5) $${\left({\text{H}}_{2}\text{O}\right)}_{n}\text{O}{\text{H}}^{•}\text{O}{\text{H}}^{-}\to {\left({\text{H}}_{2}\text{O}\right)}_{n-1}\text{O}{\text{H}}^{•}\text{O}{\text{H}}^{-}+{\text{H}}_{2}\text{O}$$

**Fig. 3:**
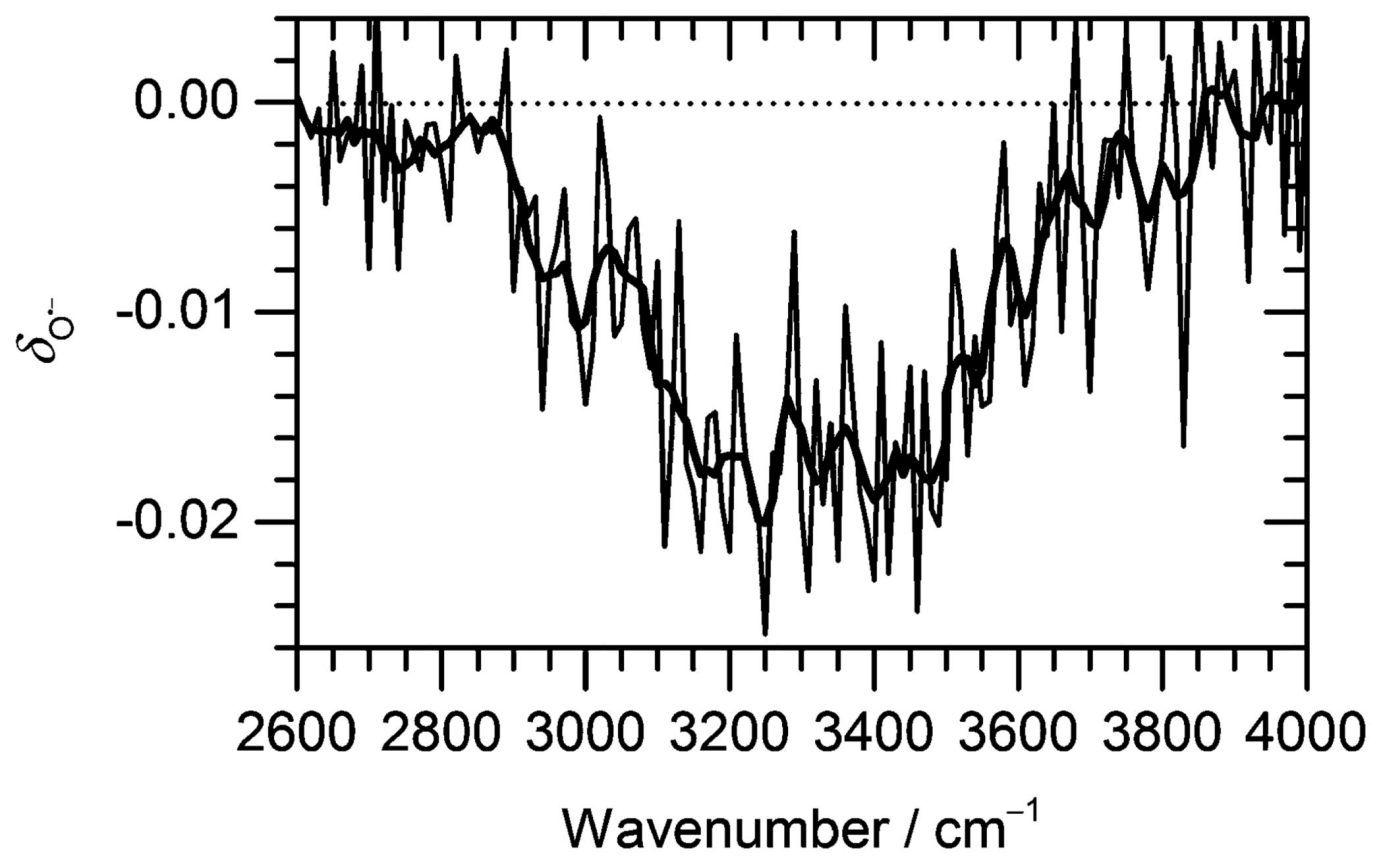
Change of the normalized intensity of (H_2_O)_*n*_O^•−^ cluster ions for the size of $\bar{n}\approx 47$ as a function of laser frequency.

Under the present experimental conditions, IR irradiation of H_2_O)_*n*_O^•−^ and (H_2_O)_*n*_OH^−^ cluster ions with $\bar{n}\approx 47$ at the absorption maximum leads to evaporation of about 10 water molecules per second (see Fig. S1 in the ESI†). If the OH^•^ radical is evaporated, the ratio of (H_2_O)_*n*_O^•−^ : (H_2_O)_*n*_OH^−^ abundance in the mass spectrum decreases. Fig. 3 documents the change of normalized intensities of (H_2_O)_*n*_O^•−^ ions after IR irradiation with respect to BIRD, calculated *via* eqn (6).

(6) $$\delta_{{\text{O}}^{•-}}={\left(\frac{I_{{\text{O}}^{•-}}}{I_{{\text{O}}^{•-}}+I_{\text{O}{\text{H}}^{-}}}\right)}_{hv+\text{BIRD}}-{\left(\frac{I_{{\text{O}}^{•-}}}{I_{{\text{O}}^{•-}}+I_{\text{O}{\text{H}}^{-}}}\right)}_{\text{BIRD}}$$

Here, *δ*_O^•−^_ describes how the ratio changes after irradiation, *I*_O^•−^_, *I*_OH^−^_ are the intensities of (H_2_O)_*n*_O^•−^ and (H_2_O)_*n*_OH^−^ cluster ions summed over all *n*.

The decrease in (H_2_O)_*n*_O^•−^ intensity starts to appear in the region of high absorption cross section of the OH stretches and matches well with the IRMPD spectrum of the corresponding clusters from 2900 cm^−1^ to 3700 cm^−1^. The OH^•^ radical evaporation induced by IRMPD, *i.e*. corrected for BIRD effects, takes place in the absorption region, with the average decrease of (H_2_O)_*n*_O^•−^ intensity reaching 2% at the absorption maximum.

By analyzing the relative probability of OH^•^ dissociation with respect to H_2_O dissociation, we can estimate the difference in activation energy for evaporation of OH^•^ and H_2_O. We assume that the (H_2_O)_*n*–1_OH^•^OH^−^ isomer is exclusively present and consider that there are on average 46 water molecules per OH^•^. Within dissociation of 10 H_2_O molecules, there is a probability of 2% to dissociate an OH^•^ radical. Then, we obtain the following ratio between the rates *k*_OH^•^_ and *k*_H_2_O_ (see ESI† for details):

(7) $$\frac{k_{\text{O}{\text{H}}^{•}}}{k_{{\text{H}}_{2}\text{O}}}=46\cdot \left(1-0.98^{1/10}\right)=0.093$$

The difference between the activation energies at the cluster temperature of 100 K can be estimated from the Arrhenius equation, assuming the respective Arrhenius prefactors to be equal, eqn (8).

(8) $$\Delta E_{\text{a}}=-RT\;\text{ln}\;\frac{k_{\text{O}{\text{H}}^{•}}}{k_{{\text{H}}_{2}\text{O}}}=-RT\;\text{ln}\;0.093=2.0\;\text{kJ}\;{\text{mol}}^{-1}$$

In reality, we can expect (H_2_O)_*n*_O^•−^ to be present in equilibrium with (H_2_O)_*n*–1_OH^•^OH^−^. Then Δ*E*_a_ would be even smaller due to the underestimation of the ratio between *k*_OH^•^_ and *k*_H_2_O_. If we consider the equilibrium constant of eqn (3) to be *K* = 1 (*i.e*. equal amounts of (H_2_O)_*n*_O^•−^ and (H_2_O)_*n*–1_OH^•^OH^−^), the difference between the activation energies is reduced to 1.4 kJ mol^−1^.

Table 1 summarizes reaction energies of H_2_O and OH^•^ dissociation calculated for *n* = 7–11. OH^•^ dissociation is consistently higher in energy than H_2_O dissociation, in agreement with experiment. OH^•^ dissociation requires about 61 kJ mol^−1^, dissociation of a water molecule from both (H_2_O)_*n*_O^•−^ and (H_2_O)_*n*_OH^−^ clusters has comparable energy of about 55 kJ mol^−1^. The difference between OH^•^ and H_2_O dissociation can be tentatively assigned to the electronic structure of the radical causing subtle differences in the strength of the hydrogen bond.

Fig. 3 shows that the OH^•^ OH^−^ isomer is formed prior to evaporation, but it does not reveal when it is formed. Reaction (3) might proceed quantitatively directly after O^•−^ formation, but there may as well be an equilibrium between the almost isoenergetic O^•−^ and OH^•^OH^−^ ions, with rapid interconversion in both directions. It is interesting to note that during the course of the geometry optimization, interconversion between O^•−^ and OH^•^ OH^−^ was occasionally observed. This indicates that the equilibrium between the isomers depends on the topology of the hydrogen bonded network, rather than on the presence of O^•−^ or OH^•^OH^−^ in the starting geometry. The ongoing making and breaking of hydrogen bonds in the clusters stored in the ICR cell and exposed to room temperature black-body radiation might thus induce the isomerization between O^•−^ and OH^•^OH^−^.

## Conclusion

We have shown that IRMPD spectra of hydrated O^•−^ and OH^−^ ions are almost identical, dominated by water–water interactions. The presence of the hydrated ions, however, introduces a broad, weak absorption in the 2400–2900 cm^−1^ region. DFT calculations reproduce the spectral shape and indicate that the spectral fingerprint of the water–ion interaction extends over the whole spectrum (2400–3700 cm^−1^). We show that to reproduce the experimental spectra, contributions from several local minimum structures with various bonding motifs have to be taken into account. Evaporation of OH^•^ is experimentally observed, indicating that hydrated O^•−^ can be interconverted into OH^•^OH^−^. These two isomers cannot be distinguished using either mass spectrometric or infrared spectroscopic techniques. From the measured mass spectra, we deduce that the difference between activation energies for OH^•^ and H_2_O evaporation in this system is 2 kJ mol^−1^, which agrees within error limits with calculations for clusters of smaller size.

## Supplementary Material

† Electronic supplementary information (ESI) available. See DOI: 10.1039/c7cp04577h

Supporting Information

## Figures and Tables

**Table 1:** Reaction energy (in kJ mol^−1^) of various reactions in O^•−^ and OH^−^ water clusters. Calculated at the B3LYP+D2/TZVP level of theory. Only the most stable structures were considered for each isomer

Reaction	*n*					
	6	7	8	9	10	11
(H_2_O)_*n*_O^•−^ → (H_2_O)_*n*–1_OH^•^OH^−^	−2.0	0.5	1.6	2.9	−1.8	−6.4
(H_2_O)_*n*_O^•−^ → (H_2_O)_*n*–1_O^•−^ + H_2_O		64.5	49.7	56.9	50.4	54.8
(H_2_O)_*n*_O^•−^ → (H_2_O)_*n*–1_OH^−^ + OH^•^		72.5	56.9	63.3	58.5	55.4
(H_2_O)_*n*_OH^−^ → (H_2_O)_*n*–1_OH^−^ + H_2_O		65.3	50.5	55.1	58.0	45.6
