# Supplementary material for: Infrared spectroscopy of O•− and OH− in water clusters: evidence for fast interconversion between O•− and OH•OH−†
Source: Phys Chem Chem Phys. Author manuscript; Available in PMC 2020 Mar 27. (PMC7100789; doi:10.1039/c7cp04577h)
Supplement: Supporting Information [file EMS85924-supplement-Supporting_Information.pdf]

This journal is © the Owner Societies 2017

Electronic Supplementary Information for article:

Infrared spectroscopy of $O^{\bullet-}$ and OH^- in water clusters: Evidence for fast interconversion between $O^{\bullet-}$ and $OH^{\bullet}OH^-$

Jozef Lengyel, Milan Ončák, Andreas Herburger, Christian van der Linde, Martin K. Beyer*

Institut für Ionenphysik und Angewandte Physik, Leopold-Franzens-Universität Innsbruck, Technikerstraße 25, 6020 Innsbruck, Austria.

* Corresponding author: martin.beyer@uibk.ac.at

Experimental methods

The IRMPD experiments are performed using a 4.7 T Fourier-transform ion cyclotron resonance (FT-ICR) mass spectrometer (Bruker/Spectrospin CMS47X) equipped with a tunable optical parametric oscillator (OPO; EKSPLA NT277). A detailed description of the FT-ICR can be found elsewhere.[1,2] The reactant ions, $(H_2O)_n^{\bullet-}$, are produced by laser vaporization of a solid zinc target and supersonic expansion of water vapor with He as a buffer gas through a nozzle into the vacuum.[3,4] The anionic water clusters are transferred from the ion source via an electrostatic lens system through differential pumping stages into the ICR cell with a background pressure below 8×10^{-10} mbar, in which the ions are stored. N_2O is introduced through a leak valve at constant pressures at around 1×10^{-8} mbar. The reactions of anionic water clusters with N_2O produce both $(H_2O)_nO^{\bullet-}$ and $(H_2O)_nOH^-$ product ions.[5–7] The reaction and the purity of product ions are monitored by recording mass spectra at different reaction delays.

$(H_2O)_nO^{\bullet-}$ and $(H_2O)_nOH^-$ clusters are irradiated at specific frequencies with tunable IR laser radiation. The laser radiation is introduced through a CaF_2 window at

the rear end of the vacuum system into the ICR cell. The diode pumped Q-switched laser, operated at 1000 Hz repetition rate with pulse duration of approximately 6-9 ns, provides a bandwidth of 8 cm^{-1} for the IR radiation. The system offers tuning from 2400 to 4000 cm^{-1} and produces pulses with energy of ~90-190 µJ, measured at the chamber entrance. To eliminate water absorption, the relative humidity is lowered by introducing dry nitrogen along the IR laser beam pathway.

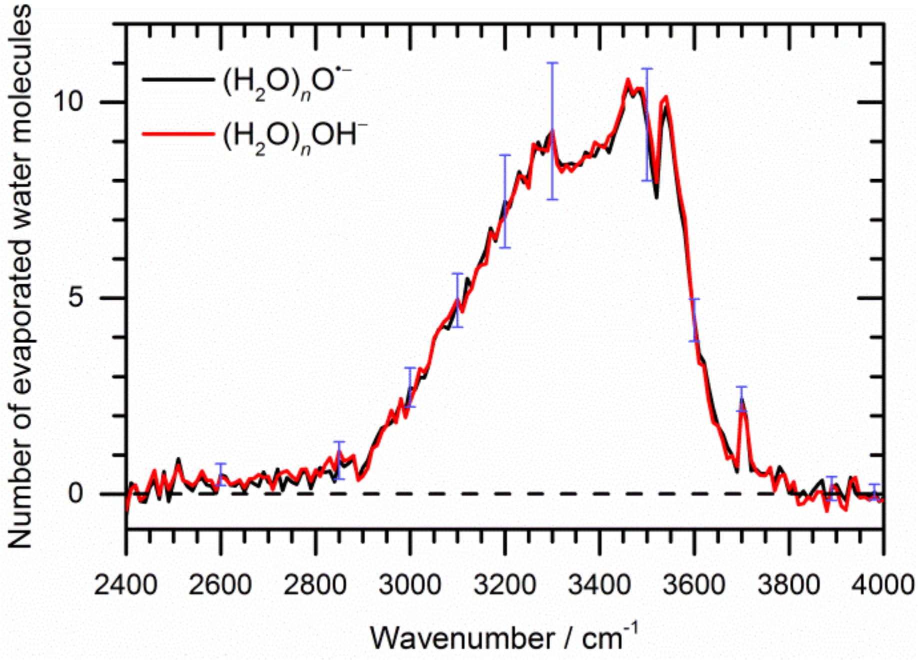

Figure S1. Raw IRMPD spectra of $(H_2O)_nO^{\bullet-}$ and $(H_2O)_nOH^-$ cluster ions, $\bar{n} \approx 47$, measured in the range of 2400-4000 cm^{-1}, with the number of evaporated water molecules per second as a function of the irradiation energy. Note that different shape of the spectrum with respect to Figure 1 is caused by the changes of laser pulse energy with wavelength. The quality of the correction can be judged by the removal of the pronounced dip around 3520 cm^{-1}.

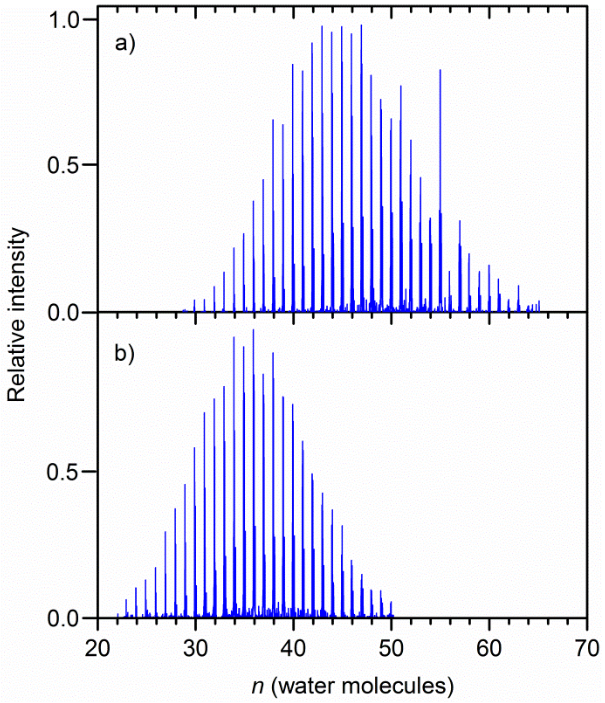

Figure S2. A typical mass spectrum as retrieved after 1 second of storage in the ICR cell, a) without irradiation and b) with irradiation at 3500 cm^{-1}.

Evaluating the relative probability of H_2O and $OH^\bullet$ evaporation

The relative activation energy of $OH^\bullet$ evaporation with respect to H_2O evaporation is derived based on the following observations and assumptions:

1) Within evaporation of 10 H_2O molecules, there is a probability $p_{\mathrm{OH}^\bullet}$ of 2% to evaporate an $OH^\bullet$ radical.

2) There are on average 46 water molecules per one $OH^\bullet$ in the cluster.

3) We assume that the $(H_2O)_{n-1}OH^\bullet OH^-$ isomer is exclusively present.

In the experiment, 10 water molecules are being dissociated in the 3200–3500 cm^{-1} region, see Figure S1. We can deduce the probability $p_{\mathrm{H_2O}}$ that in a specific evaporation event, H_2O is evaporated:

$$p_{\mathrm{H_2O}} = (1 - 0.02)^{1/10} = 0.98^{1/10} \quad \text{(S1)}$$

The probability of evaporating an $OH^\bullet$ radical, $p_{\mathrm{OH}^\bullet}$, is equal to the ratio of dissociation rates of $OH^\bullet$ and H_2O, $k_{\mathrm{OH}^\bullet}$ and $k_{\mathrm{H_2O}}$, with the factor of 46 to correct for the number of water molecules in the cluster:

$$p_{\mathrm{OH}^\bullet} = \frac{k_{\mathrm{OH}^\bullet}}{46 \cdot k_{\mathrm{H_2O}}} = 1 - 0.98^{1/10} \quad \text{(S2)}$$

Finally, the ratio of the rate constants is:

$$\frac{k_{\mathrm{OH}^\bullet}}{k_{\mathrm{H_2O}}} = 46 \cdot (1 - 0.98^{1/10}) = 0.093 \quad \text{(S3)}$$

Expressing $k_{\mathrm{OH}^\bullet}$ and $k_{\mathrm{H_2O}}$ via the Arrhenius equation assuming identical prefactors, the difference in activation energy is obtained:

$$\Delta E_a = -RT \ln \frac{k_{\mathrm{OH}^\bullet}}{k_{\mathrm{H_2O}}} = -RT \ln 0.093 = 2.0\ \mathrm{kJ/mol} \quad \text{(S4)}$$

With respect to assumption No. 3, we can also evaluate the energy difference when $(H_2O)_{n-1}OH^\bullet OH^-$ and $(H_2O)_nO^{\bullet -}$ clusters are present in equal amount, i.e. 50%:50%. Then the ratio between the number of $OH^\bullet$ and H_2O is two times smaller, i.e.

there is on average one $OH^\bullet$ per 92 H_2O water molecules. Equations (S3) and (S4) then change to:

$$\frac{k_{\mathrm{OH}^\bullet}}{k_{\mathrm{H_2O}}} = 2 \cdot 46 \cdot (1 - 0.98^{1/10}) = 0.186 \qquad \text{(S5)}$$

$$\Delta E_a = -RT \ln \frac{k_{\mathrm{OH}^\bullet}}{k_{\mathrm{H_2O}}} = -RT \ln 0.186 = 1.4\ \mathrm{kJ/mol} \qquad \text{(S6)}$$

Very small energy differences are sufficient to rationalize the preferential evaporation of H_2O, which is observed experimentally. This is due to the low internal temperature of the clusters, which is maintained by evaporative cooling.

References

1. C. Berg, T. Schindler, G. Niedner-Schatteburg and V. E. Bondybey, *J. Chem. Phys.*, 1995, **102**, 4870.
2. R. F. Höckendorf, O. P. Balaj, C. van der Linde and M. K. Beyer, *Phys. Chem. Chem. Phys.*, 2010, **12**, 3772.
3. M. Beyer, C. Berg, H. W. Görlitzer, T. Schindler, U. Achatz, G. Albert, G. Niedner-Schatteburg and V. E. Bondybey, *J. Am. Chem. Soc.*, 1996, **118**, 7386.
4. M. K. Beyer, B. S. Fox, B. M. Reinhard and V. E. Bondybey, *J. Chem. Phys.*, 2001, **115**, 9288.
5. S. T. Arnold, R. A. Morris, A. A. Viggiano and M. A. Johnson, *J. Phys. Chem.*, 1996, **100**, 2900.
6. O. P. Balaj, C.-K. Siu, I. Balteanu, M. K. Beyer and V. E. Bondybey, *Int. J. Mass Spectrom.*, 2004, **238**, 65.
7. D. Šmídová, J. Lengyel, A. Pysanenko, J. Med, P. Slavíček and M. Fárník, *J. Phys. Chem. Lett.*, 2015, **6**, 2865.

Structures optimized at the B3LYP+D2/TZVP level (coordinates in Å, energies in Hartree)

```
19
o.6h2o, E= -533.966610
O -1.585537 1.814750 -0.286301
O 0.139328 1.079446 1.814839
O 2.199729 1.090065 -0.153891
O 0.218711 0.284595 -1.604561
O -2.178985 -0.928318 -0.708348
O -0.499398 -1.643707 1.440652
O 1.543613 -1.815005 -0.528956
H -0.908484 1.512117 -0.938972
H -2.151946 1.020796 -0.262092
H -0.530827 1.439858 1.194504
H 0.958051 1.090590 1.282677
H 1.462699 0.845414 -0.822831
H 2.435577 2.002290 -0.344526
H -1.424097 -0.671254 -1.275656
H -1.735681 -1.284422 0.091157
H 0.238784 -1.866830 0.839178
H -0.302126 -0.722509 1.714769
H 2.236569 -1.242049 -0.177444
H 1.021785 -1.178604 -1.088233

19
o.6h2o, E= -533.965053
O -0.139912 -0.625682 -1.371936
O 1.995121 -1.745147 -0.169263
O -2.302464 -1.551390 -0.190271
O 1.269301 0.196499 1.821844
O -1.498873 0.527242 1.569524
O 1.828037 1.305134 -0.722337
O -1.105174 1.836950 -0.905701
H 1.245838 0.646357 -1.155753
H 1.714473 1.095997 0.228478
H -0.881760 0.933722 -1.259102
H -0.220380 2.219378 -0.809527
H -1.470460 -1.317528 -0.712835
H -2.997376 -1.050828 -0.629663
H 1.443032 -0.651889 1.374006
H 0.290681 0.282736 1.844958
H -1.821224 -0.300521 1.160673
H -1.442056 1.134164 0.802474
H 1.172707 -1.506452 -0.679092
H 2.598232 -1.033979 -0.419489

19
o.6h2o, E= -533.964016
O 0.488919 1.123361 -1.019141
O -0.157591 -1.490676 -1.213623
O -0.051471 -1.144664 1.654857
O 1.576378 1.175483 1.427331
O -2.551155 -0.917668 0.261441
O -1.995232 1.734435 -0.444056
O 2.705008 -0.480968 -0.747847
H 2.025105 0.188197 -1.007274
H 2.197941 -1.301507 -0.784668
H 0.145448 -1.400814 0.734905
H 0.431407 -0.300449 1.759800
H 0.037115 -0.536825 -1.389009
H -1.094581 -1.483953 -0.938274
H 2.293945 0.599487 1.121258
H 1.164666 1.426252 0.559783
H -1.883180 -0.980503 0.969403
H -2.500350 0.022502 -0.014491
H -1.044298 1.553022 -0.743229
H -1.892072 2.220156 0.380090

19
o.6h2o, E= -533.963191
O -0.811247 -0.196089 1.484544
O -2.705049 1.249077 -0.026515
O -1.965023 -1.478817 -0.643255
O 1.576916 -1.374585 1.102748
O 2.891222 0.983443 -0.249284
O 0.188144 1.582837 -0.060018
O 0.749984 -0.833047 -1.500199
H -2.232823 0.892244 0.756116
H -2.726102 0.448891 -0.578731
H -0.111537 0.986542 0.710416
H -0.660026 1.926305 -0.379216
H 0.698331 -1.000333 1.399932
H 2.163709 -0.606060 1.035882
H 2.696538 0.342941 -0.946643
H 2.037307 1.449093 -0.158592
H -1.700313 -1.273682 0.280720
H -1.121821 -1.427472 -1.127215
H 1.069828 -1.255019 -0.672130
H 0.487335 0.054005 -1.184704

19
o.6h2o, E= -533.963166
O 2.175795 1.164754 0.365847
O 0.343625 1.169653 -1.810688
O -0.095182 -1.650588 -1.383924
O 1.492623 -1.553437 0.718810
O -0.108227 0.242240 1.724574
O -1.580018 1.800134 0.108431
O -2.240653 -0.994623 0.469917
H 1.451501 1.062179 1.023071
H 1.685437 1.271711 -0.474493
H -0.370391 1.508638 -1.224661
H 0.187228 0.209826 -1.833610
H 0.089357 -2.424086 -1.923030
H 0.540836 -1.689584 -0.604680
H 2.108218 -0.834582 0.479727
H 0.859260 -1.029984 1.306861
H -2.177037 1.042323 -0.008493
H -1.018258 1.458492 0.854212
H -1.691308 -1.340396 -0.252074
H -1.568534 -0.659598 1.113447

19
o.6h2o, E= -533.962475
O 0.198428 1.630335 0.093647
```

O 2.029562 -1.392491 -0.447704
O 2.845368 1.263566 0.430213
O -0.390744 -0.455717 -1.440833
O -2.645030 0.872632 -0.871272
O -0.530938 -2.209139 0.563404
H 0.441410 -2.165094 0.587968
H -0.697086 -1.811614 -0.325265
H -1.901601 0.293953 -1.214034
H -2.191545 1.714848 -0.744616
H 0.060239 0.959913 -0.635547
H -0.319046 1.241524 0.830640
H 1.271682 -1.052906 -0.981150
H 2.405032 -0.597300 -0.031711
H 3.172588 1.545344 -0.429672
H 1.873721 1.473577 0.397830
H -2.205187 0.314051 0.947162
O -1.593451 0.143279 1.688600
H -1.215774 -0.736019 1.469947

19
o.6h2o, E= -533.962351
O -0.810516 0.024200 1.346625
O -1.166549 2.039456 -0.247559
O 1.519097 1.521065 -1.056020
O -2.858522 -0.435771 -0.462477
O -0.526743 -2.243120 -0.052829
O 1.990386 -1.350937 -0.931867
H -1.051063 1.335107 0.480685
H -1.068224 2.886802 0.196318
H 1.781365 1.323121 -0.133810
H 0.566453 1.715597 -0.973280
H -2.305085 -0.338816 0.347976
H -2.679837 0.401047 -0.910106
H -1.277115 -1.969645 -0.601126
H -0.585969 -1.593419 0.690898
H 1.799581 -0.450243 -1.250457
H 1.114088 -1.739794 -0.730081
H 0.952031 0.196035 1.588330
O 1.928841 0.281693 1.458162
H 2.145819 -0.458475 0.862378

19
o.6h2o, E= -533.962346
O 0.243964 -0.207696 2.068917
O -0.835928 1.755870 0.618710
O -2.566091 0.416955 -0.523534
O -1.291766 -1.754607 0.170269
O 1.162919 1.561974 -1.124040
O 0.852658 -1.197055 -1.656668
O 2.585111 -0.343086 0.541280
H -0.271784 -0.891860 1.610619
H 1.844747 -0.295977 1.186920
H 2.210577 -0.867815 -0.187768
H 0.822456 -0.216838 -1.642007
H 0.124704 -1.481226 -1.076514
H -1.884382 -2.509260 0.213501
H -1.853909 -0.960555 -0.103064
H -1.901552 1.092622 0.003295
H -2.397367 0.593523 -1.454833
H 1.833534 1.197997 -0.518001
H 0.425769 1.844676 -0.519175
H -0.159728 0.635865 1.727560

19
o.6h2o, E= -533.962209
O -2.926511 -0.167140 -0.628045
O -0.559467 0.764911 -1.297933
O -0.496157 1.550025 1.255557
O 1.898759 1.666241 -0.284191
O 2.883185 -0.996350 0.199082
O -1.244699 -1.184288 1.398256
O 0.477014 -1.645801 -0.794675
H -2.053301 0.182561 -1.007664
H -3.317836 0.604629 -0.206697
H -0.744240 1.476460 0.300193
H 0.460224 1.704302 1.153046
H 1.117877 1.424903 -0.833953
H 2.392644 0.831416 -0.191307
H -0.904872 -0.278251 1.551301
H -1.979797 -1.019399 0.773304
H 2.036944 -1.308568 -0.226652
H 2.680322 -1.007970 1.139910
H -0.089552 -1.688123 0.004011
H 0.144600 -0.822744 -1.239904

19
o.6h2o, E= -533.961098
O -0.562524 1.472003 -0.159037
O -3.238085 1.230020 0.114041
O -2.921910 -1.693875 -0.224892
O 2.264141 -0.473972 1.625538
O 2.461373 -1.042604 -1.226767
O 1.947285 1.564704 -0.353078
O -0.127433 -1.137660 0.190891
H -2.267519 1.409108 -0.027185
H -3.269073 0.818710 0.984080
H -0.337686 -0.160733 0.076488
H 0.526105 -1.320892 -0.504928
H 0.901300 1.608296 -0.293834
H 2.258167 2.451631 -0.549772
H 2.334983 -0.073787 -1.205295
H 2.705322 -1.211685 -0.302309
H 2.289131 0.375730 1.149287
H 1.367807 -0.800315 1.417889
H -3.133292 -0.762889 -0.398539
H -1.958027 -1.682095 -0.079446

19
o.6h2o, E= -533.960069
O 2.231968 -0.868091 1.315971
O 0.056118 0.927100 0.798133
O 2.289931 1.636759 -0.454420
O -0.584584 -1.502661 0.109816
O -3.438120 -0.928428 -0.143672
O -2.370085 1.746453 -0.039724
O 1.717399 -0.973254 -1.542125
H 0.816748 -1.154668 -1.214821
H 1.846430 -0.013815 -1.417506
H 1.491687 -0.225166 1.331130
H 2.268817 -1.120308 0.376842
H -0.030563 -2.045868 0.682524
H -0.467106 -0.577065 0.493379
H -1.468859 1.502223 0.319217
H -2.210151 1.927189 -0.971034

H -3.220743 0.017538 -0.051810
H -2.568558 -1.353878 -0.080910
H 1.438678 1.606741 0.061570
H 2.882602 1.134045 0.119591

19
o.6h2o, E= -533.959844
O 0.023809 -1.482322 0.538162
O 1.905242 0.009724 -0.058217
O 0.098805 1.991029 0.216722
O 4.605203 -0.226675 -0.278569
O -2.266006 1.272133 -1.066068
O -2.194640 -1.609261 -1.019990
O -2.045234 0.128320 1.584731
H 0.366371 -2.285083 0.939794
H 0.876926 -0.868962 0.263911
H -1.307412 -1.667654 -0.592248
H -2.765717 -1.412649 -0.266784
H -1.293775 -0.445125 1.332006
H -2.283695 0.597334 0.763474
H -1.369166 1.600735 -0.840877
H -2.146906 0.353091 -1.362769
H -0.340571 1.663318 1.013102
H 0.831871 1.332555 0.054482
H 4.799777 0.701495 -0.438306
H 3.614868 -0.232631 -0.199947

19
o.6h2o, E= -533.958906
O -0.370664 0.720600 0.830170
H -2.031811 1.139983 0.362180
O -2.932446 1.143947 -0.060383
H -2.719360 1.001484 -0.990460
O -2.066961 -1.506263 0.643976
H -2.749976 -0.838166 0.464426
H -1.382958 -0.949132 1.067728
O 2.127201 1.650663 0.140287
H 1.169657 1.471819 0.325740
H 2.542923 1.087029 0.809231
O 1.842119 -0.915400 1.534011
H 1.015331 -0.389252 1.485549
H 2.004048 -1.147105 0.602957
H -0.496734 0.351249 -0.941921
O -0.697427 -0.335848 -1.613679
H -1.154370 -1.003864 -1.068655
H 1.200039 -0.794644 -1.648535
O 2.140939 -0.767846 -1.396557
H 2.261130 0.151772 -1.090844

19
o.6h2o, E= -533.957026
O -0.000924 0.398101 -0.010581
O -2.184140 1.960818 0.165191
H -1.351090 1.409754 0.178147
H -2.156399 2.405163 -0.687506
O -3.948889 -0.316260 -0.138705
H -3.258867 -0.986932 -0.002779
H -3.473724 0.520319 0.013697
H -1.016338 -0.982170 0.253867
O -1.499183 -1.851507 0.274882
H -1.122232 -2.318827 -0.478318
O 1.504981 -1.850736 -0.283075
H 1.022278 -0.981744 -0.271087
H 1.107258 -2.320086 0.458201
O 3.947514 -0.313967 0.163192
H 3.259859 -0.985868 0.021466
H 3.472687 0.521679 0.004863
O 2.183576 1.960558 -0.168465
H 1.350816 1.409505 -0.189401
H 2.142288 2.413154 0.679328

19
o.6h2o, E= -533.956156
O 0.136053 -0.771737 0.320884
O -2.495580 0.904205 -1.067258
H -2.337746 -0.052500 -1.181508
H 0.126334 0.855760 0.338310
H -1.614649 1.290269 -0.908354
O -0.038932 1.831345 0.150345
H -0.554028 2.116070 0.913621
O 2.263059 0.148573 -1.075874
H 1.524283 -0.349837 -0.629530
O 4.500334 -0.392295 0.467151
H 4.027979 -0.669049 1.258201
H 3.773552 -0.191746 -0.165326
O -2.075219 -1.913387 -0.606863
H -2.584133 -1.728705 0.193686
H -1.147881 -1.728505 -0.298898
H -1.341742 -0.274473 1.409644
O -2.204866 0.089162 1.703955
H -2.544521 0.498290 0.888842
H 1.993756 1.067510 -0.957405

22
o.7h2o, E= -610.430675
O 1.571294 -1.751992 0.569579
O 0.428221 0.025210 2.377889
O -0.916714 -2.041541 -0.745222
O -0.353193 0.032018 -2.103294
O -1.541575 1.701444 -0.606383
O 2.154470 0.330595 -1.203482
O 0.989804 2.122839 0.607453
O -2.122967 -0.387110 1.186958
H 0.741661 -2.027698 0.136054
H 1.260367 -1.190433 1.316482
H -0.502807 -0.128858 2.117046
H 0.679733 0.835847 1.883027
H 0.093691 2.143288 0.222119
H 1.506428 1.591671 -0.039085
H 2.077700 -0.452079 -0.611622
H 1.392905 0.240636 -1.817224
H -2.097882 0.415410 0.631248
H -1.840179 -1.094695 0.576353
H -2.002758 2.380078 -1.105885
H -1.105656 1.054898 -1.298360
H -0.713488 -1.236453 -1.375232
H -1.164437 -2.783322 -1.302916

22
o.7h2o, E= -610.430068
O -0.003466 -1.902719 -1.240946
O 0.001513 0.632834 -1.600463
O -2.070407 -1.420336 0.642834
O 2.065050 -1.426672 0.641991

O 2.481426 1.063921 -0.588249
O -2.477411 1.070216 -0.589661
O -0.000544 -0.164301 2.090387
O 0.003975 2.245560 0.730533
H -0.003252 -2.410489 -2.056663
H -1.445459 -1.773716 -0.017299
H -2.383985 -0.584525 0.236892
H -1.662921 0.923728 -1.139036
H -2.151440 1.684676 0.083042
H -0.001635 -0.907339 -1.509969
H 1.439288 -1.777211 -0.018933
H 2.381893 -0.591587 0.237171
H 0.772522 -0.646615 1.728557
H -0.774890 -0.644741 1.728783
H 0.002378 1.424463 1.278303
H 0.002985 1.916676 -0.187572
H 1.667341 0.919310 -1.138609
H 2.156081 1.679340 0.083915

22
o.7h2o, E= -610.428228
O 0.003589 0.869813 -1.556455
O -0.518027 2.105346 0.777660
O 2.209144 -1.292620 0.481868
O 0.129676 -0.342016 2.128584
O -1.775938 -1.688385 0.601182
O 2.368580 1.365506 -0.394312
O -2.655889 0.741648 -0.497490
H -1.891428 0.814814 -1.103154
H -2.523537 -0.139166 -0.085687
H -1.454400 1.838413 0.717064
H -0.242384 1.926916 -0.150835
H 1.606757 -1.611082 -0.216686
H 2.457054 -0.394661 0.178282
H 1.558110 1.215163 -0.961711
H 2.020967 1.857310 0.358794
H -0.002487 0.542935 1.734672
H 0.915897 -0.712801 1.674366
H -1.179968 -1.284919 1.271178
H -1.174666 -1.873729 -0.143213
H 0.114526 -0.670333 -1.618026
O 0.191354 -1.684839 -1.457696
H 0.175644 -2.104486 -2.321779

22
o.7h2o, E= -610.427784
O 0.634097 -0.202151 -1.579731
O -1.283584 -1.684363 -0.842511
O 2.760010 0.918625 -0.276677
O -1.095104 1.868668 -1.012004
O -2.997375 0.131235 0.330758
O 1.931109 -1.837559 0.311320
O 0.527270 1.752190 1.243766
O -0.403635 -0.779624 1.769342
H 2.102148 0.661172 -0.965624
H 2.919874 0.063207 0.152167
H 1.534132 -1.420170 -0.484266
H 1.303903 -1.609002 1.019055
H -0.517059 -1.116956 -1.255388
H -0.964614 -2.590494 -0.804518
H -0.515317 1.177694 -1.413503
H -1.861498 1.382490 -0.657552
H -0.059634 1.986466 0.491147
H 1.396432 1.594231 0.821054
H -0.067195 0.139245 1.624096
H -0.725758 -1.087240 0.903898
H -2.614635 -0.627490 -0.152479
H -2.513081 0.110681 1.167810

22
o.7h2o, E= -610.427053
O 0.155922 1.454971 -1.482771
O -0.049223 -1.278395 -1.765124
O -0.239320 -1.359824 1.891148
O -2.265789 -1.204124 -0.074619
O 2.767442 0.897398 0.094497
O 0.294542 1.284737 1.416591
O 1.660868 -1.510223 0.026000
O -2.267140 1.676107 0.106230
H 2.343960 1.247951 -0.701174
H 2.540366 -0.076650 0.061743
H 0.730064 -1.460878 -1.039810
H 0.512740 -1.659073 1.319199
H 0.202426 1.397786 -0.510106
H -1.027622 -1.440732 1.314425
H 1.252788 1.285500 1.217579
H 0.100102 0.355251 1.698232
H 0.086330 0.515045 -1.758647
H 0.132757 -1.817994 -2.538619
H -1.788240 1.791289 -0.730823
H -1.539930 1.718800 0.752370
H -2.417649 -0.242277 -0.005978
H -1.586499 -1.299196 -0.773999

22
o.7h2o, E= -610.426833
O -0.540961 -0.009907 -1.570704
O -0.226260 1.692187 1.442101
O -2.203322 -1.622028 -0.337159
O 1.142834 -1.788581 -0.374248
O -0.413720 -1.046273 1.820866
O 3.319261 -0.243906 0.040494
O 1.407854 1.737556 -0.814217
H 0.612867 -1.223762 -0.991195
H 0.689497 -1.643077 0.485796
H -1.192750 -1.203979 1.255325
H -0.293037 -0.067642 1.795638
H 0.436441 1.805514 0.723428
H -1.078382 1.760931 0.967704
H 2.600216 -0.902407 -0.153522
H 3.203113 -0.047640 0.975968
H -1.599036 -1.059895 -0.922962
H -1.872862 -2.519616 -0.444165
H 0.750614 1.165173 -1.281056
H 2.170710 1.155008 -0.645981
H -1.783046 1.035562 -0.938981
O -2.449509 1.419621 -0.307890
H -2.933762 0.636482 -0.019948

22
o.7h2o, E= -610.426573
O -0.440922 0.136420 -1.710821
O 1.024428 -1.672506 -0.530984
O 1.189672 1.830733 -0.446606

O -1.926180 1.910898 -0.411716
O 3.290510 -0.168958 0.240355
O -1.787190 -2.038434 -0.484440
O 0.689502 0.075379 1.685398
O -2.159143 -0.101601 1.558796
H -1.507040 1.278094 -1.060892
H -1.173967 2.444865 -0.129087
H 0.600777 -0.997996 -1.147320
H 0.262782 -2.253989 -0.358864
H 0.644299 1.261446 -1.063410
H 2.069101 1.419657 -0.451621
H -1.536174 -1.355347 -1.138952
H -2.039756 -1.501694 0.295252
H -2.240840 0.645453 0.930076
H -1.220266 -0.060458 1.821826
H 0.761209 -0.653283 1.034674
H 0.751009 0.860895 1.101713
H 2.712412 -0.862026 -0.129166
H 2.871042 -0.001065 1.095911

22
o.7h2o, E= -610.425770
O -0.653648 1.406843 0.806057
O -3.081330 0.708572 -0.096431
O 2.002255 1.497011 1.069655
O -0.505169 0.665633 -1.740627
O 0.096441 -0.942180 1.991575
O -1.577769 -1.657997 -0.237381
O 2.279576 0.353439 -1.467755
O 1.457046 -1.980619 -0.256800
H 1.009311 1.595317 0.976183
H 2.085255 0.778771 1.707376
H -0.537441 1.190430 -0.897828
H -0.773926 -0.218932 -1.435538
H -2.271109 1.044456 0.385588
H -2.899039 0.978693 -1.004528
H -0.186417 -0.028810 1.748421
H 0.743840 -1.222471 1.313718
H -1.152527 -1.579038 0.639436
H -2.299950 -0.997211 -0.193209
H 0.580479 -2.103783 -0.645049
H 1.852875 -1.227049 -0.755293
H 1.369419 0.548892 -1.771221
H 2.340010 0.835132 -0.614399

22
o.7h2o, E= -610.425498
O 2.080531 -1.332809 -0.157540
O 0.472952 -1.046945 1.929618
O 0.265472 1.636155 1.674018
O 1.848460 1.548391 -0.625401
O -0.456491 1.137268 -1.827685
O -2.098529 1.357314 0.214909
O -1.821562 -1.547918 0.403355
O -0.340296 -1.397239 -1.723096
H 2.849354 -1.852429 0.090784
H 1.511785 -1.260674 0.666017
H 0.891046 1.739824 0.920645
H 2.112555 0.616772 -0.557667
H 1.045130 1.522821 -1.226673
H -1.617906 1.409857 -0.663641
H -2.464978 -2.255240 0.312712
H -1.276001 -1.539636 -0.455302
H -0.402807 -0.376847 -1.912983
H 0.568885 -1.528768 -1.407493
H -2.219424 0.402706 0.344834
H -0.620660 1.667862 1.245951
H -0.390503 -1.325960 1.567870
H 0.409231 -0.054018 1.969526

22
o.7h2o, E= -610.425230
O 0.433908 -0.542559 -1.441796
H 2.164296 -0.563735 1.237457
O 1.851538 0.151844 1.823541
H 0.890335 0.002770 1.924501
H 1.552249 0.967420 -1.066590
O 1.980724 1.693565 -0.569180
H 1.976400 1.353610 0.348736
H 1.938876 -1.198019 -0.905644
O 2.808656 -1.308271 -0.433501
H 3.254714 -0.475025 -0.629604
H -0.861018 -1.555807 -0.867614
O -1.606953 -1.923353 -0.322724
H -2.365931 -1.353728 -0.524602
H -0.513261 0.705281 -1.145211
O -0.991943 1.490790 -0.712536
H -0.287233 2.146666 -0.622250
O -3.466596 0.396642 -0.050574
H -3.153267 0.227771 0.848222
H -2.760238 0.966589 -0.409738
O -0.978343 -0.075012 1.668605
H -0.949837 0.603508 0.965325
H -1.134015 -0.896464 1.152331

22
o.7h2o, E= -610.424940
O -0.802456 0.140298 1.693656
H -0.873088 0.753396 0.935979
H 0.162405 0.108271 1.879006
O 1.981082 0.197666 1.705885
H 1.975502 0.923673 1.047491
H 2.224109 -0.572802 1.154019
O 2.636891 -1.547804 -0.426506
H 1.833421 -1.208051 -0.927072
H 3.357227 -1.000605 -0.755823
O 0.538593 -0.353655 -1.526089
H -0.713023 0.841904 -1.235664
O -1.253923 1.534756 -0.776422
H -2.147228 1.154529 -0.693726
O -3.450297 -0.091672 0.063859
H -2.853833 -0.830985 -0.204979
H -3.154424 0.079950 0.967083
O -1.363924 -1.848056 -0.242798
H -1.080311 -1.412161 0.585011
H -0.719968 -1.478467 -0.893923
H 1.380257 1.067791 -1.047663
O 1.683073 1.870239 -0.538547
H 0.856634 2.359389 -0.434047

22
o.7h2o, E= -610.424623
O 0.444600 0.598954 -1.449353
H 1.495380 1.416561 -0.080271

O 1.857689 1.569679 0.813972
H 1.722778 0.703215 1.253780
H -0.541523 1.782326 -0.579953
O -0.968912 2.304839 0.145843
H -0.247469 2.360711 0.789426
H -0.743394 -0.379051 -1.622060
O -1.527842 -1.042229 -1.581318
H -1.489833 -1.559331 -2.390023
H 1.916983 -1.310949 0.673163
O 1.526125 -1.125494 1.546117
H 0.580920 -1.381708 1.465301
O -1.139601 -1.807112 1.093269
H -1.171993 -1.807059 0.117441
H -1.752312 -1.072039 1.287528
O -2.896300 0.348636 0.568679
H -2.290825 1.121907 0.536184
H -2.752022 -0.078902 -0.291117
H 1.896947 -0.327114 -1.356970
O 2.729874 -0.773155 -1.042295
H 3.171308 -0.061514 -0.561734

22
o.7h2o, E= -610.424393
O -0.289859 -0.102012 -1.644731
H 3.857264 -0.105806 -0.827167
O 3.393257 0.004011 0.008305
H 2.682716 -0.703966 -0.010828
H 0.686051 1.106815 -1.063619
O 1.187721 1.729474 -0.451285
H 2.028064 1.266849 -0.264743
H 0.817049 -1.207524 -0.905371
O 1.380787 -1.697032 -0.245384
H 0.807376 -1.722895 0.550957
O -0.584229 -1.387098 1.727699
H -0.541435 -0.416562 1.804709
H -1.278592 -1.538480 1.048493
H -1.570112 -1.230323 -1.074978
O -2.304385 -1.508329 -0.472459
H -2.762757 -0.656876 -0.367079
O -2.419587 1.352902 -0.604026
H -1.706581 0.984046 -1.177220
H -1.947531 1.545108 0.222765
O -0.486230 1.475765 1.554856
H 0.208605 1.617638 0.837979
H -0.299927 2.120531 2.242298

22
o.7h2o, E= -610.424345
O 2.659849 -1.212422 -0.227115
O -0.038924 -1.110295 -1.667418
O 1.996404 1.421636 0.289557
O 0.013346 0.895490 1.922747
O 0.121423 -1.542007 1.137737
O -0.254097 1.692668 -1.532389
O -1.986706 1.331075 0.388526
H 2.100346 -1.348243 -1.004485
H -0.558355 2.366074 -2.146011
H -0.965957 1.611674 -0.815744
H 1.384433 1.333110 1.076771
H 2.530347 -0.253995 -0.023894
H 1.388070 1.668372 -0.426691
H -1.309723 1.250831 1.148453
H -2.334414 0.425125 0.283299
H 0.015636 -1.214793 -0.693969
H -0.069267 -0.144117 -1.799287
H 0.081547 -0.624163 1.591468
H 1.066887 -1.692879 0.964268
H -1.703396 -1.711337 0.543421
O -2.460364 -1.498713 -0.035577
H -2.033592 -1.485116 -0.906143

22
o.7h2o, E= -610.423829
O -0.036055 0.693919 -1.011003
H 1.095345 1.320449 0.395455
O 1.629325 1.367476 1.213641
H 1.927863 0.440331 1.317690
H -0.957161 1.276030 0.379228
O -1.346366 1.403869 1.280487
H -0.547392 1.506851 1.817679
H -1.703142 0.465262 -1.438082
O -2.697450 0.411793 -1.482728
H -2.981054 1.201458 -1.009142
H 1.693022 0.448282 -1.476158
O 2.681407 0.402616 -1.485741
H 2.918419 1.058700 -0.817876
O 2.397475 -1.329399 0.808705
H 2.708741 -0.981157 -0.045945
H 1.511102 -1.685042 0.580191
O -0.087121 -1.939651 -0.274693
H -0.916859 -1.821627 0.233982
H -0.059753 -1.147544 -0.848261
O -2.509690 -1.130579 0.900310
H -2.796073 -0.809260 0.024207
H -2.145257 -0.313074 1.295213

25
o.8h2o, E= -686.889086
H 0.406933 1.002683 -1.616147
O 1.317949 1.444389 -1.521417
H 1.416445 2.035611 -2.272299
O 0.747740 2.232253 1.147032
H -0.223813 2.246685 1.035081
H 1.073134 2.100653 0.237372
O -1.940654 2.051219 0.312682
H -2.527995 1.424462 0.751879
H -1.632712 1.537082 -0.476931
O -1.007092 0.269226 -1.519487
O 0.304220 -2.157666 -1.197879
H -0.175630 -1.359519 -1.510637
H 1.215524 -1.829927 -1.025458
O -0.650603 -1.939087 1.394948
H -0.294276 -2.163611 0.503859
H -1.443063 -1.418505 1.182627
O 1.570642 -0.321802 1.995926
H 1.268668 0.596606 1.834320
H 0.765347 -0.875662 1.919948
O 2.660749 -0.845236 -0.493615
H 2.401306 -0.016832 -0.933148
H 2.397975 -0.700145 0.446483
H -2.270736 -0.442492 -0.693884
O -2.913285 -0.788219 -0.008062
H -3.094433 -1.697711 -0.264093

25
o.8h2o, E= -686.886008
H 1.134423 0.840035 -1.467845
O 1.859352 1.469448 -1.119884
H 2.217216 1.925228 -1.886368
O -0.003690 2.479150 0.798089
H -0.826161 2.254254 0.310906
H 0.704921 2.317378 0.148733
O -2.086006 1.461853 -0.763319
H -2.649991 0.835982 -0.277443
H -1.415043 0.892248 -1.218173
O -0.063434 -0.157627 -1.637080
O 1.582367 -2.397399 -0.728360
H 1.144412 -1.728421 -1.287992
H 2.140975 -1.840803 -0.142597
O -0.953550 -1.682750 0.283211
H -0.217572 -2.320172 0.260504
H -0.753826 -1.140144 -0.536466
O 0.413065 0.070878 2.139333
H 0.228279 0.973601 1.796379
H -0.153915 -0.515716 1.605934
O 2.806122 -0.412700 0.817217
H 2.702406 0.282669 0.143803
H 2.039406 -0.259706 1.415731
O -3.532256 -0.839515 0.353014
H -3.803256 -1.167376 -0.510399
H -2.668043 -1.279755 0.507527

25
o.8h2o, E= -686.885944
O 0.060561 0.038988 -1.627856
O -1.680942 -1.686704 -0.921658
O 0.091210 1.709703 1.312179
O -1.828891 1.852308 -0.700913
O 1.489096 -1.795291 -0.185500
O 3.773739 -0.454093 0.397579
O -0.534855 -0.928905 1.641091
O 2.070860 1.637970 -0.650453
H 1.080249 -1.182511 -0.849755
H 0.942526 -1.644846 0.610068
H -1.011319 -1.135258 0.818743
H -0.286674 0.029109 1.572056
H 0.887960 1.766543 0.739801
H -0.636201 1.950600 0.698002
H 3.013606 -1.049124 0.172629
H 3.611374 -0.201764 1.312333
H -1.016314 -1.007988 -1.325323
H -1.223880 -2.533307 -0.932815
H 1.423989 1.120971 -1.186326
H 2.774501 1.005710 -0.420746
H -1.222353 1.279463 -1.225195
H -2.510831 1.252599 -0.349681
O -3.432028 -0.194555 0.604140
H -3.017269 -0.861338 0.021156
H -2.879374 -0.224232 1.396198

25
o.8h2o, E= -686.885926
O 0.092371 1.386787 -0.744942
O -2.640402 1.764453 -0.586768
O 0.411539 -1.238434 -0.613670
O 2.945261 -0.868793 -1.400767
O 2.711657 1.487174 0.107823
O -1.674238 -1.863945 1.081122
O -1.064497 0.750709 1.811341
O -2.426495 -0.952594 -1.491121
O 1.581307 -0.466441 1.790484
H 0.180308 0.436126 -0.999021
H 1.010103 1.653986 -0.508447
H 2.436530 0.926962 0.863824
H 2.991421 0.812475 -0.543070
H 1.983222 -1.030485 -1.166895
H 3.430680 -1.470591 -0.827793
H -0.835207 -1.858977 0.562345
H -2.318877 -1.719601 0.365975
H -2.612676 -0.015773 -1.288638
H -1.456553 -1.032429 -1.425530
H -2.616199 1.598523 0.366761
H -1.694365 1.889513 -0.796541
H 1.264366 -0.967228 1.007332
H 0.768222 -0.057925 2.136398
H -0.638645 1.010657 0.971334
H -1.384343 -0.166565 1.653944

25
o.8h2o, E= -686.885632
O 0.265623 -0.591861 -1.337128
H -1.321307 -1.698563 -0.930861
O -2.236122 -1.898007 -0.657213
H -2.249712 -1.612745 0.281912
O -2.120028 0.652423 -1.891161
H -2.561200 -0.150693 -1.554957
H -1.200243 0.318150 -1.970227
O 0.897167 1.874288 1.123096
H 0.873710 0.937185 1.400667
H 1.418219 1.824441 0.301881
O 2.259758 0.927152 -1.184476
H 1.416633 0.311522 -1.336484
H 2.444730 1.380690 -2.010949
H -1.995636 1.506303 -0.235556
O -1.883054 1.766040 0.702509
H -0.930836 1.968996 0.803886
H -1.976528 0.176976 1.554827
O -1.913441 -0.750990 1.881054
H -0.959698 -0.915340 1.951838
H 3.350884 -1.767272 -0.368584
O 3.578953 -1.026949 0.203338
H 3.260740 -0.241145 -0.298591
H 0.637555 -0.958867 0.305055
O 0.893070 -0.952807 1.267379
H 1.857280 -1.073955 1.246956

25
o.8h2o, E= -686.884893
O 0.320501 -0.049511 -1.814327
O -0.197331 -1.926062 -0.218974
O -2.164627 0.917949 -1.324813
O -2.856856 -1.399996 0.364646
O 2.541940 -1.625693 -0.731509
O 2.424121 0.349229 1.276957
O 1.416777 2.095139 -0.588124
O -1.030236 2.211052 0.828854
H -0.082171 -1.202659 -0.967922
H 0.699191 -2.308348 -0.220249

H 1.560739 0.152191 1.672358
H 2.206186 1.054294 0.619870
H -0.833555 1.427866 1.365609
H -1.547362 1.853091 0.069533
H 1.117961 1.439060 -1.260333
H 0.594024 2.327312 -0.109562
H -1.278314 0.591340 -1.640930
H -2.598354 0.131119 -0.957223
H 2.014768 -1.079239 -1.346005
H 2.666739 -1.018145 0.027904
H -2.055726 -1.872805 0.070201
H -2.507347 -0.811643 1.046563
O -0.362795 -0.408620 1.930795
H -0.425836 -0.973696 2.706079
H -0.262893 -1.017620 1.136057

25
o.8h2o, E= -686.884818
O -0.233613 0.479046 -1.558195
O -1.463056 2.523390 -0.264844
O 2.123625 1.301112 -0.431936
O 0.486655 1.918502 1.722991
O -0.074683 -0.792666 2.060980
O -2.715101 -0.114768 -0.993395
O -2.022801 -2.322785 0.602369
O 3.339867 -1.069766 -0.493083
H -0.929558 1.982651 -0.897120
H -2.243698 1.964723 -0.151434
H -1.763107 0.075960 -1.298502
H -3.213591 -0.299833 -1.794372
H 3.721352 -1.053956 -1.375872
H 2.961011 -0.152279 -0.374391
H -1.143253 -2.469564 0.216235
H -2.383279 -1.585907 0.069547
H 1.341731 1.084923 -0.994664
H 1.708634 1.589213 0.413491
H -0.959014 -1.179411 1.947537
H 0.370512 -1.122989 1.254215
H -0.241657 2.244036 1.158732
H 0.229875 0.999533 1.951912
H 0.339887 -1.038062 -1.002073
O 0.633386 -1.828328 -0.474129
H 1.609907 -1.788944 -0.489312

25
o.8h2o, E= -686.884648
O 1.340337 0.848628 1.413679
O -0.438650 -1.036265 1.492596
O -0.573892 2.023383 -0.072354
O 2.441112 1.674175 -0.849013
O -2.769367 0.632581 1.171756
O 2.250692 -1.812446 1.047433
O -0.707046 -0.473781 -1.192355
O 2.068221 -0.998969 -1.668938
H 2.203711 1.453299 0.095911
H 1.655736 2.142003 -1.157569
H 0.161848 -0.233908 1.637335
H 0.227520 -1.746329 1.483916
H 0.121725 1.736255 0.581755
H -1.416687 1.814836 0.374865
H 2.205427 -0.886603 1.362120
H 2.282505 -1.699986 0.074876
H 2.343068 -0.074798 -1.492124
H 1.096273 -0.932520 -1.694851
H -0.615860 -0.815357 -0.270072
H -0.616571 0.496866 -1.032074
H -2.128571 -0.034186 1.471950
H -3.170980 0.229694 0.379717
O -3.407231 -0.745295 -1.261972
H -2.429593 -0.670329 -1.400544
H -3.552962 -1.675018 -1.061868

25
o.8h2o, E= -686.884267
O -0.081820 -0.277835 -1.469415
O 0.171314 0.847366 0.945993
O -2.603413 0.089884 -1.125136
O -0.257009 -2.683595 -0.277797
O -1.935986 -1.085681 1.322453
O 1.855267 -1.573109 1.191669
O 2.653317 -0.129357 -1.073407
H -0.180062 -2.036315 -1.009528
H -0.914530 -2.271822 0.323090
H -2.390745 -0.673716 0.560920
H -1.270732 -0.422721 1.567010
H 0.088763 0.541131 -0.003383
H 0.947351 1.443304 0.928284
H 1.218924 -2.148657 0.715687
H 1.320162 -0.784592 1.386272
H -1.611359 -0.059986 -1.347609
H -3.111976 -0.378607 -1.792032
H 1.719166 -0.203775 -1.387067
H 2.647693 -0.731163 -0.300191
H -1.390991 2.072643 0.741940
O -2.183744 2.504682 0.383079
H -2.531801 1.834569 -0.226458
H 2.083333 2.844845 -0.470448
O 2.471855 2.252946 0.181663
H 2.658559 1.412459 -0.319307

25
o.8h2o, E= -686.883808
O 1.862383 0.216477 0.713531
O 0.292376 -1.771652 1.341299
O -0.100170 1.798091 1.372570
O -0.319535 -1.432543 -1.421877
O -2.062307 -0.229446 1.628414
O -3.019598 -0.381636 -0.994711
O -0.830520 1.362348 -1.325623
O 2.461415 -2.225599 -0.622543
O 1.832081 2.675150 -0.694085
H 2.509204 -1.353950 -0.176675
H 1.702419 -2.116085 -1.215176
H 0.931829 -0.992472 1.230323
H 0.841613 -2.528910 1.099734
H 0.692841 1.178091 1.235367
H 0.256617 2.659142 1.119127
H 2.061210 1.829304 -0.251101
H 1.120162 2.428587 -1.299008
H -0.680054 1.513349 -0.370387
H -1.740989 1.008616 -1.370801
H -2.354072 -1.012978 -1.315085
H -2.834236 -0.340712 -0.030632
H -0.258929 -0.461300 -1.517541

H -0.246540 -1.572451 -0.457012
H -1.549277 0.597005 1.677755
H -1.380800 -0.924755 1.665322

25
o.8h2o, E= -686.883046
O 1.711178 0.180457 -1.341879
O 1.849909 1.916531 0.820655
O -0.975363 1.432888 0.860379
O -0.905956 -1.439918 0.928550
O -3.473436 0.148657 1.204200
O 1.510422 -2.210297 -0.093386
O -2.575552 -0.827951 -1.221687
O -0.600855 1.062248 -1.834917
H 1.856844 1.513795 -0.075052
H 0.921749 1.851086 1.097934
H 0.371991 0.674645 -1.702474
H -0.514912 1.847859 -2.381513
H -1.966050 -1.274496 -0.595680
H -1.995805 -0.166604 -1.645948
H 1.531381 -1.494513 -0.777211
H 2.234872 -1.925936 0.488696
H -3.382259 -0.100838 0.255990
H -2.998952 -0.571767 1.640436
H -0.705929 -0.489520 1.015008
H -0.052828 -1.846610 0.644930
H -1.906073 1.248444 1.090410
H -0.933077 1.408708 -0.121956
H 3.239408 -0.206644 -0.205342
O 3.600434 -0.365909 0.689953
H 3.173395 0.358741 1.176835

25
o.8h2o, E= -686.882468
O -0.675291 1.347486 -0.059226
O 0.780644 0.423546 2.304480
O 1.765386 -1.679865 0.765777
O -0.097915 -0.240487 -2.181545
O 2.570515 -0.116936 -1.364289
O 1.931248 2.211628 0.080982
O -3.510360 1.100105 -0.665455
O -1.865219 -0.507377 1.334643
H 0.293428 0.890505 1.601049
H 1.182148 -0.344816 1.837730
H -1.496483 0.284284 0.814501
H -1.302705 -0.503970 2.123191
H -2.556181 1.296182 -0.714611
H -3.561492 0.540288 0.118256
H 0.964599 2.045434 -0.060863
H 2.063983 1.904272 0.987725
H 2.522155 0.757551 -0.925451
H 1.703782 -0.181293 -1.820262
H -0.401804 -1.025887 -1.693468
H -0.413903 0.509681 -1.629426
H 2.167351 -1.189147 0.009802
H 0.967125 -2.088135 0.392901
O -0.826097 -2.307070 -0.322920
H -1.293439 -1.703896 0.321016
H -1.421867 -3.039294 -0.501661

25
o.8h2o, E= -686.882410
O -0.086026 2.378511 -0.738638
O 1.762138 0.483704 -1.352214
O 2.274374 1.449006 1.014129
O 0.245142 -1.575986 -1.248535
O 2.988009 -1.501903 0.227645
O -2.680510 1.378791 -0.323002
O -2.438096 -0.464302 1.796368
O -2.582785 -1.434318 -0.906823
O 0.389852 -0.656937 1.433820
H 0.545343 1.728714 -1.142289
H 0.326652 2.593746 0.105613
H 0.795277 -0.772678 -1.526596
H 0.902293 -2.263862 -1.090208
H 2.742879 -0.835683 -0.450664
H 2.248480 -1.462573 0.852986
H 2.247505 1.236307 0.035772
H 2.976643 0.875628 1.343287
H 0.860257 0.196883 1.365675
H 0.188922 -0.916009 0.512094
H -1.798195 1.757915 -0.515252
H -2.713360 0.536268 -0.812298
H -1.469985 -0.517488 1.892307
H -2.578288 0.344250 1.261659
H -1.645098 -1.581268 -1.121625
H -2.606108 -1.372690 0.067546

25
o.8h2o, E= -686.882340
O -2.509269 -0.617549 -1.646535
O -2.287251 -1.968126 0.867924
O 0.305723 -1.459478 0.201350
O 0.204036 0.692940 -1.315409
O 1.198075 2.339764 0.846262
O -0.864877 0.554302 1.895079
O -2.057008 1.827481 -0.286117
O 3.072236 -1.874314 0.147726
H -1.575071 -0.345668 -1.703831
H -2.551980 -1.171887 -0.843678
H -2.287860 -1.223722 1.486309
H -1.334468 -2.053715 0.655084
H 0.297340 -0.799303 -0.545857
H 1.237932 -1.738846 0.302419
H 0.800184 2.129713 -0.017997
H 0.614302 1.869993 1.468199
H -1.379859 1.028802 1.203773
H -0.363169 -0.129131 1.411596
H -2.643066 1.158184 -0.679483
H -1.225018 1.645928 -0.787621
H 3.104316 -0.921580 -0.147984
H 3.287656 -2.383264 -0.639580
O 2.857634 0.637462 -0.720023
H 1.940476 0.629020 -1.110485
H 2.723888 1.245630 0.027076

25
o.8h2o, E= -686.881813
O 0.679983 0.995481 1.298743
O -1.122665 -0.731764 1.817549
O 2.967982 0.083148 0.345206
O 2.228341 -2.324026 -0.597982
O -0.938986 1.863052 -0.800456
O -0.570951 -2.452970 -0.334018

O -1.974874 -0.553295 -1.801042
O -3.235956 0.636389 0.524831
O 1.931069 2.638286 -0.608723
H 1.564120 2.296862 0.233289
H 1.142225 2.611757 -1.170513
H 2.211595 0.359156 0.931411
H 2.975713 0.807003 -0.302098
H -0.376962 -0.026639 1.666796
H -1.052974 -1.015703 2.732925
H -0.439544 1.590982 0.002706
H -1.126078 1.033797 -1.290036
H -2.658172 -0.366258 -1.133761
H -1.462468 -1.299148 -1.417265
H 0.396106 -2.413787 -0.478826
H -0.734065 -1.954606 0.490941
H 2.627474 -2.911108 0.050564
H 2.511350 -1.413406 -0.318612
H -2.668057 1.336706 0.158346
H -2.621804 0.129972 1.091278

28
o.9h2o, E= -763.350251
O 1.516738 1.445422 1.200213
O 0.837012 0.903008 -1.408930
O -1.417401 2.016546 -0.885403
O 2.364058 -1.188773 1.205329
O 0.003332 -2.373232 0.471112
O -1.965846 -1.057690 1.520868
O -2.930495 -0.323472 -1.047454
O -0.495768 -1.404753 -2.047255
O -1.191354 1.624556 1.885259
H -0.093693 -2.141117 -0.484870
H 0.883635 -2.021034 0.747295
H -1.408227 -1.107630 -1.855461
H 0.038857 -0.576598 -2.018451
H -2.561286 0.580787 -1.064890
H -2.765315 -0.621761 -0.133558
H 2.040952 -0.279885 1.401324
H 2.873442 -1.033023 0.389159
H -1.381324 1.904208 0.968408
H -1.472539 0.689198 1.901301
H 1.308949 1.412024 0.237318
H 0.637663 1.564729 1.618540
H -1.473188 2.858897 -1.344076
H -0.504803 1.598819 -1.133995
H -2.400658 -1.626126 2.161960
H -1.172669 -1.582571 1.162826
H 2.513912 0.431442 -1.292045
O 3.446256 0.238619 -1.009639
H 3.604039 0.907791 -0.333583

28
o.9h2o, E= -763.350202
O 0.889170 -1.300129 -1.056261
O -0.136001 -1.906720 1.352175
O -1.700424 -0.850680 -1.759986
O -2.805199 -1.365831 0.530526
O -2.440257 1.357061 1.259479
O 0.299791 0.794379 1.647002
O 1.075145 1.348634 -1.079781
O 3.113484 1.277757 0.871179
O 3.449398 -1.193702 -0.355899

H 3.874184 -0.980135 -1.192435
H 2.479474 -1.319863 -0.597106
H 3.337220 0.370388 0.568115
H 2.364199 1.156386 1.477094
H 1.904888 1.526711 -0.593928
H 1.082823 0.372949 -1.278565
H 0.200097 -0.188488 1.666110
H 0.400808 1.004773 0.699278
H -1.530206 1.264040 1.606523
H -2.728250 0.436650 1.117809
H -2.440603 -1.207221 -0.402110
H -3.579049 -1.927276 0.436657
H -1.095656 -1.886489 1.187144
H -0.742193 -1.066811 -1.602339
H 0.271458 -1.950696 0.451891
H -1.737568 0.132756 -1.802079
O -1.665820 1.897729 -1.401771
H -0.695685 1.945080 -1.314669
H -2.000240 1.849276 -0.480704

28
o.9h2o, E= -763.347357
O -1.392655 2.602488 -0.461473
O -1.600900 0.559346 1.487554
O 0.861206 0.946423 1.869883
O -3.156193 0.359570 -0.750260
O -1.641368 -1.869717 -1.314452
O 0.745074 -0.889100 -1.640692
O 1.817425 -1.449686 0.878210
O -1.024496 -2.119122 1.355121
O 1.340355 1.673610 -0.752341
H -1.507950 -2.134195 -0.372536
H -2.263004 -1.106253 -1.252110
H -0.052007 -2.103965 1.351066
H -1.289668 -1.184678 1.549186
H 1.597012 -0.626692 1.359635
H 1.481954 -1.299619 -0.028915
H -2.708779 1.160190 -1.075653
H -2.858229 0.356966 0.188698
H 1.251723 1.509042 0.211040
H 1.056704 0.834493 -1.167477
H -1.477952 2.085817 0.366733
H -0.462275 2.476858 -0.719745
H 1.052745 1.526607 2.610900
H -0.178374 0.819449 1.809736
H 1.212662 -1.254845 -2.395918
H -0.190157 -1.272657 -1.619551
H 3.200245 0.844996 -0.785830
O 3.885093 0.166418 -0.666415
H 3.467024 -0.473342 -0.070344

28
o.9h2o, E= -763.345693
O 0.270343 -0.157429 -1.544753
O 2.517663 1.233159 -0.769646
O -1.011266 1.947503 -0.624433
O 1.064882 2.527705 1.130443
O 3.862471 -1.177995 0.046334
O 1.214063 -1.638643 0.385502
O -0.773697 0.128354 1.679275
O -2.255065 -1.272928 -1.374073
O -3.451424 0.944931 0.021750

H 1.755695 0.747886 -1.175728
H 3.128808 0.534641 -0.475269
H 0.896004 -1.213567 -0.474472
H 0.873723 -1.017883 1.051556
H -1.369362 -0.878096 -1.571238
H -2.800843 -0.534596 -1.045839
H -0.940821 0.612396 0.851705
H -1.163712 -0.766027 1.547940
H -0.527539 1.286359 -1.190865
H -0.325818 2.397360 -0.086547
H 4.067923 -1.637546 -0.773604
H 2.934254 -1.449493 0.258367
H 0.765206 1.812508 1.707077
H 1.714838 2.105254 0.515542
H -3.248314 0.652562 0.917987
H -2.662983 1.478743 -0.226965
O -1.517145 -2.453324 0.963515
H -1.883372 -2.164754 0.090514
H -0.580286 -2.616398 0.768525

28
o.9h2o, E= -763.345229
O -0.098615 -0.060024 -1.524990
O 1.706605 1.528604 -0.430912
O 3.470353 -0.245044 0.655931
O -1.518918 1.273629 0.253957
O -3.786301 -0.224318 0.508923
O 1.913537 -1.935941 -1.102778
O -1.967418 -1.981871 -0.920848
O 0.490445 0.204057 1.834475
O 0.080362 -2.346388 0.943715
H -1.081942 0.801899 -0.527275
H -0.970385 0.959443 1.009090
H 1.016351 0.622048 1.128131
H 0.348524 -0.736668 1.553174
H -0.737549 -2.336822 0.400517
H 0.789348 -2.404979 0.265938
H -3.048414 0.427665 0.431277
H -3.641286 -0.644748 1.362714
H 1.079823 0.968681 -0.983099
H 1.262686 2.390811 -0.338074
H -1.358764 -1.322862 -1.332980
H -2.709759 -1.457491 -0.574164
H 1.272137 -1.281967 -1.462675
H 2.578737 -1.412356 -0.620325
H 3.033528 0.539455 0.262598
H 2.946847 -0.415683 1.448854
H -0.813955 2.870424 -0.040110
O -0.230478 3.620988 -0.310608
H -0.442505 3.763604 -1.238520

28
o.9h2o, E= -763.345158
O -0.270316 0.027776 -1.162311
O -2.024739 -1.696075 -0.429735
O 1.526835 -2.153088 -0.977555
O 0.956652 0.045496 1.411446
O 1.897783 1.856148 -1.151258
O 0.524777 2.728166 1.157865
O -4.019667 0.138066 0.086891
O -0.004017 -2.591114 1.397306
O 3.426788 -0.294377 -0.024815

H 0.924860 -1.413096 -1.203870
H 1.127807 -2.512419 -0.160346
H -0.826667 -2.369235 0.923775
H 0.303781 -1.729035 1.731978
H 0.755006 -0.038474 0.463134
H 0.779764 1.002815 1.577908
H 2.958066 -1.043836 -0.448175
H 2.881376 -0.144671 0.765319
H -1.332237 -1.024661 -0.793189
H -2.077808 -2.405195 -1.077634
H 1.131428 1.278202 -1.343183
H 2.587704 1.224392 -0.867576
H -4.143075 0.499581 -0.796952
H -3.390839 -0.605888 -0.043796
H -0.379495 2.666018 0.787261
H 1.101672 2.648751 0.368751
O -1.833009 2.066773 -0.227949
H -1.310086 1.388834 -0.719816
H -2.539949 1.555749 0.197314

28
o.9h2o, E= -763.344512
O 1.193179 1.463676 1.793254
O 2.076954 -0.899651 1.348655
O -0.040700 -2.568429 0.913389
O -1.523952 1.595823 1.394978
O -2.408033 -0.910197 1.196001
O 0.239451 -1.851045 -1.608484
O 2.525383 -0.523661 -1.307128
O 1.465093 2.125405 -0.976170
O -1.323450 1.859242 -1.349208
O -1.952903 -0.600467 -1.647967
H 1.533110 0.504134 1.663298
H 1.696804 1.839231 2.519941
H 2.375126 -0.825642 0.403396
H 1.333402 -1.553501 1.312026
H 0.523178 2.058660 -1.223342
H 1.459429 2.030720 -0.006836
H -0.574028 1.578954 1.643601
H -1.524708 1.855296 0.449399
H 0.058219 -2.476874 -0.083975
H -0.864902 -2.093865 1.110569
H -1.569191 0.875433 -1.524604
H -1.816077 2.387183 -1.982639
H -3.135517 -1.033061 1.811216
H -2.079309 0.022963 1.331159
H 1.742523 -1.073969 -1.592873
H 2.219752 0.402075 -1.363018
H -2.315681 -0.864835 -0.786880
H -1.070313 -1.158467 -1.738995

28
o.9h2o, E= -763.344175
O 2.431505 -0.367582 1.861956
O 3.358000 0.680179 -0.724561
O 1.081958 1.979435 -0.530060
O -0.145812 0.535326 1.376302
O -2.973269 -0.505344 1.546568
O -2.824681 -1.695240 -1.057435
O -0.193851 -1.433060 -0.387511
O 2.500422 -2.003083 -0.389991
O -2.284319 1.958214 0.353341

H 1.500792 -0.042286 1.766998
H 2.941266 0.195101 1.259763
H 4.022223 1.261210 -1.104021
H 2.497164 1.194698 -0.730222
H 0.693412 1.623524 0.311873
H 0.410634 1.751787 -1.202227
H -2.019479 -0.335756 1.655948
H -3.040152 -1.006581 0.711489
H -1.880915 -1.877062 -0.867631
H -2.772904 -0.913272 -1.623712
H 0.731198 -1.745290 -0.514655
H -0.182019 -0.910706 0.453257
H -1.471894 1.682906 0.839460
H -2.921397 1.311927 0.705827
H 2.893274 -1.223849 -0.820159
H 2.518724 -1.718836 0.548736
H -0.754670 -0.000338 -1.463624
O -1.138271 0.790828 -1.891548
H -1.658701 1.235439 -1.183594

28
o.9h2o, E= -763.343567
O -0.663304 2.011563 -1.246955
O 1.878352 2.130457 0.042022
O 2.640317 0.030504 -1.286246
O 0.065576 -0.501169 -1.772742
O -2.243686 -2.026330 -0.797996
O -3.843221 -0.080177 0.476217
O -1.825655 1.538306 1.196326
O 0.676254 0.317143 1.889865
O 2.904211 -1.600197 1.071200
H -0.481326 1.113887 -1.635474
H 0.205840 2.298459 -0.919807
H 2.583283 2.748838 0.250197
H 2.281907 1.385829 -0.507682
H 1.717091 -0.221021 -1.596272
H 2.871722 -0.614992 -0.590309
H -1.636018 -1.459203 -1.312417
H -2.848340 -1.407458 -0.341993
H -3.114373 0.492494 0.837822
H -4.216647 0.441797 -0.240677
H -1.072345 1.118517 1.652552
H -1.462835 1.762667 0.305085
H 2.066095 -2.056099 0.852810
H 2.595036 -0.912634 1.678610
H 1.083711 0.971227 1.290810
H 0.464642 -0.469209 1.340147
O 0.315283 -1.986645 0.325663
H 0.327162 -1.533980 -0.578315
H -0.597606 -2.326779 0.336071

28
o.9h2o, E= -763.343182
O 1.934787 -1.090700 1.832357
O 3.475582 0.316067 -0.235131
O 1.492804 2.001876 0.036234
O -0.293098 0.463432 1.328894
O -3.112223 -0.218437 1.124588
O -2.750072 -2.372748 -0.757376
O -0.444084 -0.969328 -0.923537
O 2.067323 -2.076297 -0.759993
O -0.467759 1.726909 -1.888020

H 1.119211 -0.538296 1.716696
H 2.648596 -0.532995 1.487833
H 4.282388 0.808264 -0.407065
H 2.735075 0.996763 -0.190268
H 0.920620 1.584489 0.727285
H 0.898486 2.032843 -0.749878
H -2.157236 -0.115946 1.310328
H -3.160316 -0.942607 0.474994
H -1.894697 -1.919444 -0.959199
H -2.499306 -3.107378 -0.188672
H 0.433576 -1.408368 -1.019920
H -0.435180 -0.593035 -0.005229
H -1.170347 2.042679 -1.279039
H -0.492808 0.757466 -1.777145
H 2.671791 -1.329000 -0.915506
H 1.984487 -2.036378 0.217398
H -1.407804 1.885814 0.824510
O -2.110466 2.314507 0.287529
H -2.818899 1.652888 0.368534

28
o.9h2o, E= -763.341708
O -1.973577 1.750861 -1.464547
O 0.717777 1.870189 -0.440335
O 0.904227 -0.462911 -1.588189
O -1.372930 2.248004 1.235086
O -1.195458 -0.239640 2.292770
O -2.216957 -2.074628 0.680416
O 0.670550 -1.792748 0.806939
O 3.025539 1.262420 0.660869
O -1.818274 -1.037926 -1.951258
H -0.321028 -0.544249 1.989744
H -1.281006 0.696301 1.971868
H 2.853327 1.282210 1.606816
H 2.158831 1.566512 0.232139
H 0.638268 -1.291154 -0.042545
H -0.113858 -2.359045 0.776484
H -1.964063 2.116635 0.468889
H -0.506537 2.327336 0.762835
H -0.845498 -0.979377 -1.995920
H -2.005656 -1.429960 -1.078985
H -1.010431 1.867703 -1.337554
H -2.065136 0.805877 -1.700819
H 1.841547 -0.730497 -1.550386
H 0.877077 0.501414 -1.289664
H -3.065826 -2.430626 0.955469
H -1.994144 -1.332732 1.312960
O 3.349488 -1.265564 -0.389373
H 3.348891 -0.366637 0.014803
H 2.732168 -1.764166 0.164848

28
o.9h2o, E= -763.341138
O 0.190622 1.394529 0.502586
O 1.193117 -0.659724 1.727413
O 0.592242 -0.027779 -1.717581
O 0.540600 -2.437815 -0.189292
O -2.389582 2.094655 -0.004535
O -2.300364 0.165852 -2.002725
O -2.226229 -1.710836 0.047083
O -1.714469 0.032613 2.190072
O 3.111825 -0.813607 -0.508294

O 2.974261 2.187397 -0.078464
H 2.389236 -0.499759 -1.083932
H 2.736528 -0.796695 0.384848
H 2.024988 2.025157 0.057603
H 3.332578 1.305698 -0.251929
H -1.084724 0.635711 1.755121
H -1.938434 -0.622314 1.493276
H -1.413044 2.037681 0.152235
H -2.749508 1.616199 0.754674
H 0.520783 0.673512 -1.020276
H 0.342608 -0.854545 -1.264737
H 0.900024 0.217383 1.305152
H 0.546030 -0.784127 2.435007
H 1.393162 -2.688677 -0.563456
H 0.771253 -1.865478 0.586848
H -1.413742 -2.206845 -0.132987
H -2.343071 -1.121122 -0.732291
H -2.441159 0.929780 -1.400059
H -1.349690 0.196164 -2.205195

28
o.9h2o, E= -763.341019
O -0.569450 -1.138362 0.500313
O 0.893394 0.513801 1.787394
O -2.845050 -0.135642 1.573590
O -2.342279 2.044731 -0.077574
O -1.099815 0.256483 -1.816857
O 0.675200 2.124830 -0.490196
O 3.080799 0.676945 -1.272581
O -3.026813 -1.898140 -0.769228
O 1.890101 -1.908112 -0.801129
O 3.416516 -0.645198 1.241165
H -2.156480 -1.898406 -0.322382
H -2.855823 -1.355930 -1.550044
H -1.994338 -0.601177 1.376213
H -3.443313 -0.580034 0.953708
H 0.328866 -0.221881 1.347693
H 0.436632 0.759159 2.597360
H 0.993772 -1.728085 -0.457489
H 2.165792 -1.081067 -1.239759
H 3.408273 0.422316 -0.388716
H 2.401012 1.351711 -1.101809
H 0.214431 1.515805 -1.098059
H 0.777116 1.627800 0.347634
H -0.812308 -0.427731 -1.180601
H -1.722206 0.823299 -1.312501
H -2.520070 1.403933 0.645940
H -1.452377 2.398980 0.067660
H 3.008330 -1.334551 0.681017
H 2.641864 -0.204827 1.634959

28
o.9h2o, E= -763.340353
O 2.861277 -1.230030 0.239100
O 0.244563 -2.004623 -0.444286
O 0.825384 -1.545390 2.166231
O 0.228768 -0.248277 -2.247234
O -0.520565 0.797078 2.287045
O 0.674410 2.444627 0.651408
O -1.288352 1.759144 -1.215495
O -2.763294 0.538331 0.609091
O 2.423072 1.176625 -1.212482

O -2.557862 -2.045563 -0.814499
H -3.609557 0.984938 0.697999
H -2.271996 1.008439 -0.126776
H -1.379062 0.626329 1.853164
H -0.046105 -0.075673 2.312689
H -2.762488 -1.256499 -0.291301
H -1.592264 -2.157854 -0.678637
H -0.789335 1.028241 -1.658666
H -0.617149 2.206766 -0.666876
H 1.716974 -1.309655 1.844522
H 0.455914 -1.960505 1.351181
H 1.760125 0.716756 -1.762044
H 1.892442 1.657031 -0.551596
H 2.045305 -1.628501 -0.138267
H 2.907348 -0.364382 -0.211121
H -0.158288 -0.615947 -3.046848
H 0.233167 -1.031684 -1.538268
H 0.928532 3.231636 1.140032
H 0.267232 1.805202 1.319790

28
o.9h2o, E= -763.339953
O 0.253734 1.151145 1.553017
O 2.938300 1.038138 0.515195
O 3.092193 -1.525077 -0.796792
O 0.449011 -1.994906 -0.415039
O -0.330306 -1.260566 2.097033
O -0.209726 0.309291 -2.051774
O -2.801287 0.073782 -1.086755
O -2.103369 2.172191 0.659683
O -2.233171 -2.336665 0.085367
H 2.106121 0.986626 1.028485
H 3.014574 0.190786 0.040038
H -0.129001 -0.251500 1.986964
H 0.121977 -1.545362 2.895576
H -2.559828 -1.503698 -0.336125
H -1.881652 -2.043401 0.940833
H -1.309899 1.778076 1.110611
H -1.712835 2.762881 0.005174
H -2.737173 0.778064 -0.410297
H -1.952875 0.170339 -1.574073
H 0.145477 1.133258 -1.655643
H 0.151245 -0.414321 -1.509828
H 3.465125 -2.150053 -0.167914
H 2.140716 -1.764606 -0.841784
H 0.356658 -1.727864 0.527532
H -0.451941 -2.341717 -0.588212
H 0.549669 2.226962 0.213854
O 0.810567 2.546920 -0.683954
H 1.756065 2.321510 -0.673035

31
o.10h2o, E= -839.808945
O 1.782146 2.412538 -0.510784
O -0.622549 1.346296 -0.704146
O -0.060639 -0.859297 -1.827858
O 2.587497 -0.095623 -1.715382
O -3.233746 2.060653 -0.382178
O 0.576023 -2.275215 0.377646
O -0.588322 -0.117578 1.593060
O -2.177389 -2.211253 -0.766064
O -3.260714 -0.425957 1.050770

O 3.077201 -1.226987 0.783265
O 1.946881 1.069794 2.027976
H -2.301692 1.854633 -0.639573
H -3.149823 2.741633 0.292448
H 0.836889 2.115837 -0.650967
H -0.602795 0.883083 0.172510
H 2.484741 0.833647 -1.455879
H 1.662077 -0.410580 -1.875056
H 1.970502 1.641049 1.240441
H 1.026909 0.751742 2.077096
H -1.566889 -0.237757 1.622134
H -0.219357 -0.960973 1.227447
H -1.623292 -2.764281 -0.200857
H -1.502763 -1.698433 -1.287765
H -0.480639 0.564352 -1.351320
H 1.516278 -2.078750 0.606326
H 0.484596 -1.972707 -0.555398
H 3.064258 -0.868332 -0.132269
H 2.812084 -0.463100 1.334143
H -3.429431 0.396902 0.555276
H -3.016822 -1.088224 0.363980
H 1.824067 3.341305 -0.753162

31
o.10h2o, E= -839.808218
O -3.920412 -0.002832 0.063720
O -1.402516 0.542530 1.694463
O -1.726522 1.540768 -1.052123
O 1.121618 1.734282 2.018324
O -1.654369 -1.054796 -1.309114
O 2.833973 -0.093152 0.993831
O 1.668656 -2.416299 0.664673
O 0.883736 -1.495390 -1.896085
O 2.300464 0.671517 -1.656514
O -1.116166 -2.116890 1.067602
O 0.745448 2.753248 -0.544928
H -3.467191 0.109194 0.911292
H 2.823777 0.779661 -2.454955
H 1.717644 -0.144493 -1.800889
H 2.854799 0.244237 0.078092
H 2.250501 0.550934 1.476268
H 1.302311 2.105394 -1.016288
H -0.168633 2.475173 -0.749495
H -0.105232 -1.348465 -1.758113
H 1.169183 -2.029075 -1.133687
H 0.250482 1.298871 2.123901
H 1.011471 2.246325 1.185517
H -1.418879 -1.979504 0.134722
H -0.161546 -2.301347 0.999559
H -1.406064 0.910619 0.792134
H -1.273992 -0.428683 1.569785
H -3.278290 -0.530255 -0.460106
H -1.651186 0.582844 -1.353950
H 2.216453 -3.106487 1.046962
H 2.123151 -1.538623 0.860239
H -2.660030 1.599792 -0.801797

31
o.10h2o, E= -839.807687
O -0.243023 0.146446 -1.996288
O 0.138858 2.437555 -0.309374
O -3.057597 0.732620 -1.108091
O 2.463745 1.767492 0.667335
O 2.673362 0.168964 -1.643157
O -0.243676 -1.978886 -0.526270
O -3.113058 -1.659709 0.276046
O 2.442214 -2.203030 -0.092075
O 1.732992 -0.632697 2.060255
O -1.000087 -0.683327 1.645517
O -1.931020 1.797617 1.273046
H -2.155948 0.650609 -1.455721
H -0.432588 -0.096305 -2.907490
H -2.574936 -2.271902 -0.241520
H 0.704483 -2.247045 -0.438133
H 2.793071 0.764009 -0.878884
H 1.724900 0.235289 -1.846848
H 1.563754 2.088190 0.349952
H 2.946088 2.544554 0.961690
H -3.226731 -0.143005 -0.692499
H -0.485608 -1.616314 0.379829
H 2.691355 -1.482783 -0.703739
H -2.530655 1.559541 0.538409
H -0.605581 2.295171 0.337747
H -0.022013 1.780255 -1.007092
H 2.015491 0.242319 1.744685
H 2.298955 -1.732693 0.764106
H 0.743998 -0.623317 2.007900
H -1.644104 0.918883 1.636466
H -2.444053 -1.295594 0.921649
H -0.261553 -0.714228 -1.446061

31
o.10h2o, E= -839.807674
O -0.773323 -2.295966 -0.469638
O -3.575527 -1.693416 -0.391859
O -2.334578 0.666667 0.134214
O -0.388997 0.141103 -1.572097
O 0.381054 2.666453 -0.954262
O 2.240157 -0.434175 -1.678994
O 2.013225 -2.548035 0.091815
O 2.013725 -0.533337 2.033826
O -0.732169 -0.799909 1.929743
O -1.581407 3.284638 0.765218
H -0.626538 -1.455697 -0.985193
H -1.743102 -2.377730 -0.422715
H -3.779904 -1.542122 -1.320185
H -3.291532 -0.807317 -0.070364
H -1.646074 0.530128 -0.585672
H -2.312123 1.620205 0.350988
H 1.274907 -0.256441 -1.832986
H 2.253686 -1.274699 -1.164932
H 2.071614 -1.940674 0.863343
H 1.062231 -2.713429 -0.028812
H 1.035178 -0.488269 2.108585
H 2.269888 0.224828 1.479427
H -0.827430 3.149536 0.127004
H -1.191549 3.128576 1.631365
H -1.315216 -0.150514 1.489766
H -0.671995 -1.492456 1.242687
H 0.070964 1.804863 -1.338304
H 1.236800 2.437844 -0.547243
O 2.723621 1.437040 0.067964
H 2.616412 0.719196 -0.638693
H 3.627535 1.755672 -0.005492

31
o.10h2o, E= -839.806645
O 2.601005 1.860324 -0.940786
O -0.007454 1.611066 -0.565638
O -2.398435 0.253341 -0.471010
O 0.908060 -0.411548 -2.061138
O 0.246364 1.148139 2.094969
O -1.486236 -1.984273 -1.871700
O 2.761212 0.180319 1.374164
O -0.600200 -2.903693 0.603033
O -1.632588 -0.940309 1.958580
O 1.919547 -2.073685 0.012268
O -2.350773 3.259953 -0.156609
H 2.908705 2.757765 -0.784136
H -0.655216 -1.605873 -2.203087
H -1.952519 -1.209258 -1.488084
H -0.961090 -2.746764 -0.299186
H 0.364976 -2.707678 0.503607
H -1.547332 0.748649 -0.564196
H -2.347860 -0.115988 0.430432
H 2.285388 -1.374694 0.601089
H 1.618238 -1.569813 -0.768189
H 0.034106 1.606240 1.257282
H -0.400295 0.416837 2.142526
H 2.890308 0.741420 0.587028
H 1.914684 0.505563 1.751585
H 1.607729 1.883732 -0.792565
H 0.484638 0.325275 -1.533021
H -2.118872 -1.276070 2.716012
H -1.222046 -1.739003 1.489179
H 1.711174 -0.004842 -2.406391
H -2.860335 2.445450 -0.265596
H -1.438394 2.921983 -0.183356

31
o.10h2o, E= -839.806570
O 2.405456 -1.221218 -1.187760
O 0.964084 0.705599 -2.194339
O 1.677781 2.490953 -0.258855
O -0.972241 2.578962 0.571239
O -1.550670 0.456790 1.837770
O 0.550825 -0.854322 2.372047
O -1.758820 0.985004 -1.604715
O 0.664188 -2.770889 0.360940
O -1.525970 -2.193485 -1.190276
O -3.382541 -0.539042 0.100336
O 2.751574 0.348752 1.167728
H 1.809208 -0.531109 -1.619473
H 3.031530 -1.500413 -1.860870
H 2.815662 -0.240976 0.395458
H 2.019028 -0.029659 1.697238
H -2.886683 -1.327766 -0.195485
H -2.891803 -0.217610 0.892844
H 0.790851 2.650284 0.121622
H -1.554586 3.296201 0.834806
H -0.775709 -2.479729 -0.617074
H -1.242233 -1.346986 -1.558890
H 1.241814 1.442358 -1.592143
H -0.011209 0.674036 -2.111585
H -2.449514 0.474868 -1.125419
H -1.504539 1.669254 -0.953166
H 0.597369 -2.165306 1.129520
H 1.350913 -2.366621 -0.202149
H -0.323888 -0.309360 2.192886
H 0.497689 -1.182885 3.273502
H 2.103676 1.837108 0.338556
H -1.206904 1.757469 1.166888

31
o.10h2o, E= -839.805960
O 0.078773 -0.035241 -1.259555
O 1.775438 1.722688 -0.496537
O 1.757087 -1.925664 -0.120321
O -1.887933 1.972454 -1.140375
O -3.602007 0.130676 0.003753
O -2.053003 -1.894320 -1.248340
O -0.729512 -2.717571 1.027219
O -0.898275 -0.054838 1.382454
O -0.332809 2.784944 1.093481
O 3.950929 -0.232660 -1.137200
H -1.212648 1.293734 -1.359781
H -1.476912 2.464407 -0.403409
H 0.493182 2.521025 0.643352
H -0.582881 2.001646 1.604632
H -0.874558 0.099449 0.421829
H -0.877674 -1.049636 1.441236
H -3.149785 0.876729 -0.452330
H -3.102353 0.049689 0.827484
H 1.117840 1.033932 -0.899531
H 1.980938 2.360349 -1.187048
H -1.306411 -1.293860 -1.453327
H -2.748945 -1.293703 -0.914135
H 3.442300 -1.034725 -0.938114
H 3.343685 0.476285 -0.878577
H 0.185558 -2.718979 0.682996
H -1.284081 -2.646396 0.216387
H 1.207174 -1.347344 -0.698332
H 1.953867 -1.353213 0.649835
O 1.912460 0.081126 1.847537
H 2.185103 0.777609 1.231711
H 0.937418 0.130260 1.848203

31
o.10h2o, E= -839.805426
O -3.609603 0.118531 0.404811
O -2.063165 -1.540590 -1.273554
O -0.086978 -0.817374 0.535935
O 1.759482 -2.669204 -0.326367
O -1.335749 1.589198 -0.037323
O 3.598869 -0.567720 0.341742
O 1.596790 0.297143 -1.375732
O 1.205124 2.788761 -0.065387
O 1.754307 0.882972 1.921702
O -0.909328 0.612574 -2.649037
O -1.944435 -0.745499 2.523983
H -3.266956 -0.527321 -0.248618
H -3.252631 -0.211794 1.252358
H 1.011047 0.331102 1.619274
H 1.658061 1.689406 1.370887
H 3.118568 -0.063503 1.031536
H 3.147548 -1.431330 0.310834
H -1.640974 0.011443 3.035578
H -1.231512 -0.882996 1.847449

H 0.234921 2.757031 -0.017301
H 1.415005 2.080445 -0.705697
H 1.814458 -2.364299 -1.239743
H 0.979056 -2.176282 0.017524
H -1.107911 1.146662 -1.852919
H 0.053755 0.501906 -2.604160
H -1.350498 -1.685234 -0.626501
H -1.684135 -0.885540 -1.899018
H -2.266493 1.300808 0.111686
H -0.817809 0.828668 0.304251
H 0.983735 -0.101590 -0.712121
H 2.480259 0.092085 -1.001475

31
o.10h2o, E= -839.804602
O 0.571021 -2.252996 -0.672196
O 3.010296 -1.357485 -0.603672
O -1.243126 -0.625727 -1.698300
O -2.940732 -1.665061 0.050644
O -0.932489 -1.858799 1.709623
O 0.144492 1.496912 -0.977750
O 0.243529 0.714200 1.658702
O 3.049115 0.592927 1.522069
O -3.362103 0.982774 -0.642314
O 2.913635 1.379912 -1.211481
H -0.074421 -1.680948 -1.193867
H 0.181356 -2.293147 0.218614
H -1.136709 -2.401321 2.475538
H -1.772752 -1.797664 1.154198
H -2.383004 -1.516754 -0.763798
H -3.369320 -0.787120 0.110794
H -0.313072 0.731591 -1.434436
H 1.092814 1.507145 -1.229542
H 3.028521 0.416806 -1.308005
H 3.050144 1.479001 -0.249461
H 3.568672 -2.096192 -0.859293
H 2.059571 -1.697410 -0.642623
H -2.671754 0.611200 -1.229663
H -2.901375 1.678262 -0.133097
H 3.124848 -0.221282 0.995730
H 2.093930 0.652358 1.733900
H -0.160617 -0.168029 1.761893
H 0.217707 0.908562 0.691778
O -1.628090 2.812238 0.680324
H -1.251366 2.299755 1.411412
H -0.987559 2.624029 -0.035262

31
o.10h2o, E= -839.804298
O 0.513315 1.680784 -0.889628
O 3.884196 -0.740678 -0.818391
O 1.056666 -0.932927 -1.304729
O 2.105945 -1.089426 1.417387
O 2.617058 1.464619 0.554375
O -1.291153 3.220339 0.423115
O -2.117354 0.946662 -1.467045
O -0.590227 -1.341017 2.264899
O -2.497546 -2.268076 0.469886
O -1.547055 -1.726897 -2.056032
H 3.044521 -0.895029 -1.288432
H 3.749984 0.142880 -0.436216
H -0.528841 2.798216 -0.051179
H -2.006283 3.122804 -0.218166
H 0.343715 -1.412436 2.004291
H -1.106993 -1.907122 1.654832
H -1.181422 1.244004 -1.474711
H -2.264738 0.744658 -0.519629
H 0.862384 0.043702 -1.303471
H 1.120006 -1.146563 -0.358274
H 1.794671 1.631501 -0.041988
H 2.741260 2.255370 1.086102
H 2.854228 -1.426779 0.898560
H 2.219357 -0.118267 1.323799
H -2.905735 -1.410411 0.647478
H -2.178518 -2.193679 -0.461781
H -1.870717 -0.801418 -2.050954
H -0.587673 -1.614098 -1.904449
O -2.220571 0.690460 1.333557
H -1.865577 1.599107 1.330357
H -1.539821 0.112818 1.744677

31
o.10h2o, E= -839.802962
O -0.224741 -0.569848 1.410888
O -2.970436 -1.139118 1.036621
O -0.037588 -2.335469 -0.734160
O -2.335542 -0.902897 -1.690328
O -1.165949 1.293233 -0.357690
O 1.374533 1.449441 2.075263
O 2.440906 -1.266229 1.818488
O 1.235068 2.560855 -0.521352
O 2.865391 0.902449 -2.008393
O 2.587106 -1.533417 -0.971597
H -2.026971 -0.979864 1.257456
H -3.372929 -0.256134 0.960492
H 0.709893 0.721022 1.868732
H 0.951532 2.013700 2.729082
H 1.444419 2.276978 0.388906
H 1.820291 2.021761 -1.098485
H 1.457934 -1.242805 1.757800
H 2.638208 -0.354764 2.073351
H -0.184832 -1.927936 0.143865
H -0.761864 -1.979015 -1.281598
H -0.890626 0.691646 0.373600
H -0.365468 1.839834 -0.552407
H -2.680338 -1.179179 -0.815676
H -1.845318 -0.085744 -1.474231
H 3.811569 1.057314 -1.940467
H 2.728392 -0.021034 -1.651897
H 2.705311 -1.477749 -0.000739
H 1.666410 -1.868392 -1.062460
O -3.879613 1.340659 -0.131149
H -3.990747 0.775523 -0.906492
H -2.927934 1.577565 -0.181545

31
o.10h2o, E= -839.802859
O -3.139621 0.000091 -0.956645
O -0.934228 0.000055 -2.129445
O 3.997663 0.000096 -0.856247
O -2.216225 1.964141 0.758646
O -0.163863 2.322006 -1.112287
O 2.125753 2.019637 0.067053
O 1.617936 -0.000113 1.860808

O -0.945801 -0.000155 2.367977
O -2.216329 -1.964178 0.758477
O -0.163843 -2.321904 -1.112371
O 2.125862 -2.019638 0.066850
H -2.235828 0.000082 -1.538337
H -3.883035 -0.000195 -1.564907
H -2.709665 -1.358081 0.165716
H -1.788648 -1.363050 1.396496
H 1.242900 2.132170 -0.422046
H 2.436627 2.905519 0.270824
H 1.243029 -2.132058 -0.422315
H 2.436656 -2.905579 0.270488
H -0.875037 2.341690 -0.434610
H -0.390710 1.519193 -1.664359
H -0.390718 -1.519106 -1.664451
H 3.426489 0.761264 -0.662805
H 3.426630 -0.761176 -0.662799
H -1.788366 1.362894 1.396439
H -2.709699 1.358149 0.165902
H 0.040756 -0.000276 2.159342
H -1.014285 0.000005 3.326603
H -0.874936 -2.341524 -0.434602
H 1.854626 0.777027 1.308421
H 1.854772 -0.777249 1.308476

31
o.10h2o, E= -839.800695
O -3.768485 -0.810996 -0.116907
O -1.138170 -1.978775 0.122714
O -1.527689 -0.246611 -1.752260
O 1.432762 -2.070774 -0.744242
O 1.177089 0.056599 -2.465896
O -0.090542 -0.626558 2.109797
O -0.835312 1.370215 0.443902
O 1.575385 2.137840 -0.753991
O 3.718157 1.215091 0.444020
O 2.697645 -0.964134 1.589274
H -3.750545 -0.022063 0.453432
H 0.206531 0.048786 -2.532833
H 1.365085 -0.732561 -1.901079
H 4.236108 0.953144 -0.324027
H 2.896159 1.645545 0.048487
H 0.489593 -2.146050 -0.429830
H 1.942227 -1.843166 0.049488
H 0.754290 2.056168 -0.225420
H 1.460346 1.445323 -1.453754
H -0.514882 -1.331474 1.546666
H 0.868344 -0.753155 2.000325
H -1.042231 0.839815 -0.359119
H -0.541536 0.700945 1.118898
H -2.976276 -1.333204 0.133463
H -1.326356 -1.069392 -1.202544
H 3.270083 -1.259896 2.301766
H 3.107806 -0.140394 1.207689
H -2.491513 -0.195532 -1.634094
O -3.454356 1.799949 1.106360
H -2.478281 1.739986 0.964664
H -3.766810 2.342408 0.375660

31
o.10h2o, E= -839.800567
O -0.105054 -1.917195 -1.798243
O 1.697611 -0.036657 -2.123644
O 1.019065 2.430879 -1.260473
O -1.517556 2.356613 -0.052716
O -0.283094 1.115299 1.864642
O -0.904893 -1.312030 2.084574
O 1.062255 -2.512364 0.660301
O 2.992808 -0.762126 0.280946
O 2.201310 1.848173 1.195567
O -3.863278 0.734489 -0.665710
O -2.493057 -1.741386 -0.203444
H 0.516790 -1.145372 -1.970812
H -0.195537 -2.388153 -2.630785
H 0.627572 -2.514566 -0.215666
H 0.377430 -2.145355 1.270618
H -3.095515 1.313907 -0.527040
H -1.764759 3.227797 0.271280
H 2.560773 0.969368 0.983754
H 1.333771 1.659806 1.638185
H -3.499829 -0.159811 -0.525840
H 2.314903 -0.250849 -1.399642
H 1.400712 0.890069 -1.922440
H 0.078941 2.442354 -0.987065
H 1.507905 2.366736 -0.408371
H -2.071839 -1.609154 0.667378
H -1.748003 -1.723779 -0.828248
H -0.670521 -0.283509 2.043240
H -1.124031 -1.521997 2.995970
H 3.833911 -1.194794 0.449601
H 2.277671 -1.435516 0.493024
H -1.109294 1.873262 0.748464

31
o.10h2o, E= -839.800541
O -0.050072 -0.599210 1.010553
O -2.788731 1.038956 -0.705216
O -2.623260 -0.110521 1.788959
O -3.980116 -1.784796 -0.152180
O -1.425568 -1.378613 -1.103740
O 1.192767 1.665693 -0.091456
O 2.299011 0.298974 2.238202
O 1.510135 -1.738048 -1.149712
O 3.140594 0.485003 -1.716082
O 3.952636 -1.134707 0.481747
H -2.782527 0.767224 0.244707
H -2.287877 0.317762 -1.133252
H 0.776953 0.825447 0.186872
H 0.447231 2.264339 -0.313896
H 1.085696 -1.323867 -0.372933
H 2.008767 -1.005061 -1.568901
H 1.424754 -0.091072 2.007989
H 2.272777 1.133901 1.749348
H -1.668510 -0.358686 1.710365
H -3.113705 -0.881812 1.456091
H -0.947595 -1.141744 -0.249809
H -0.707590 -1.711239 -1.660604
H -4.245526 -0.915399 -0.478492
H -3.103096 -1.905873 -0.569967
H 2.513378 1.079360 -1.258626
H 3.676961 0.103009 -0.994956
H 3.456300 -0.682883 1.198345
H 3.293684 -1.711632 0.065299
O -1.102071 3.164490 -0.553357

H -1.343248 3.543395 0.297802
H -1.759429 2.437057 -0.697150

34
o.11h2o, E= -916.269324
O -3.091626 0.160483 1.739652
O -0.490257 -0.553423 1.558858
O -1.753988 0.131463 -1.787559
O -3.486919 -1.503382 -0.477831
O 0.563879 1.500446 -1.937912
O 2.929850 0.350665 -1.502149
O 3.112273 1.276107 0.936159
O 2.140031 -1.166954 1.899113
O 2.248888 -2.203246 -0.545591
O -2.396034 2.151718 -0.082300
O 0.366049 1.965580 0.841697
O -0.588192 -1.987302 -0.766182
H -2.952940 0.972601 1.201843
H -3.400855 -0.487394 1.070806
H 2.667021 -3.068417 -0.543842
H 2.229248 -1.889057 0.405175
H 2.772015 -0.601235 -1.365565
H 2.055839 0.727634 -1.803536
H 0.363271 -2.102185 -0.926337
H -0.929896 -1.254510 -1.358196
H 1.181868 -0.980620 2.015662
H 2.553245 -0.293905 1.752255
H 0.467858 1.827759 -1.022232
H -0.273331 0.976629 -2.064129
H 0.061137 1.065705 1.106679
H 1.324714 1.961693 1.004273
H -2.828325 -2.202963 -0.380995
H -3.003913 -0.851959 -1.054638
H -2.257684 1.526099 -0.838548
H -1.503032 2.413278 0.186249
H -1.423149 -0.336561 1.818936
H -0.575710 -1.100165 0.739016
H 3.930524 1.760369 1.073724
H 3.110459 0.959958 -0.024232

34
o.11h2o, E= -916.268753
O 1.007401 1.442266 -1.901752
O -1.443799 2.331097 -1.278923
O -2.936313 -0.097212 -1.382307
O 2.765278 1.556898 -0.141411
O -0.988086 -2.147182 -1.549046
O 1.282242 -1.158283 -2.196437
O 3.097773 -1.189527 -0.069745
O 1.256237 -1.500745 2.073052
O -1.101556 -2.252572 1.193809
O 1.220706 1.340957 2.127160
O -1.342347 2.020115 1.406929
O -2.783537 -0.095450 1.459285
H -0.540607 2.121081 -1.632973
H 3.347076 2.312385 -0.257522
H 2.037159 1.576965 -0.931984
H -0.410568 1.836983 1.676589
H -1.744377 -1.531928 1.371736
H -1.067193 -2.323334 0.214240
H -1.324856 2.245849 0.438555
H -1.986554 1.541891 -1.476211
H 0.357636 -1.795519 1.732300
H 1.451334 -2.053656 2.834331
H -2.275771 -0.795596 -1.552385
H -3.052353 -0.114182 -0.415105
H 3.167036 -0.211386 -0.086064
H 2.518943 -1.374037 0.692453
H 1.180271 -0.155191 -2.220357
H 1.968686 -1.307149 -1.509608
H -1.148934 -2.883654 -2.144884
H -0.074453 -1.758593 -1.807121
H -2.204609 0.750247 1.489317
H -3.497975 0.032909 2.088396
H 1.801920 1.511913 1.352332
H 1.226199 0.371097 2.219041

34
o.11h2o, E= -916.268367
O -0.961972 0.370448 -2.694637
O -2.820680 -1.004304 -1.019842
O -1.468150 -2.263660 0.832335
O 1.052641 -2.674528 -0.044727
O 2.498859 -1.037433 1.146585
O -2.859614 1.291496 0.633153
O -0.922156 2.479637 -0.979544
O 1.365583 2.564148 0.248246
O 1.137415 0.800706 2.408694
O -1.283403 -0.057045 2.526718
O 3.132555 0.601375 -0.856788
O 1.375462 -0.997991 -2.311470
H -0.106920 -0.119516 -2.612250
H -3.528128 -1.576904 -1.327604
H -1.616503 -0.177074 -2.226173
H 1.481764 -3.530779 -0.123314
H -0.552058 -2.485916 0.532079
H 1.715467 0.055360 2.045601
H 1.229993 1.520428 1.753261
H 1.213317 -1.654727 -1.606375
H 2.041233 -0.390865 -1.916183
H -0.325418 0.294711 2.474239
H -1.503633 -0.093264 3.461429
H 2.584475 1.341190 -0.537259
H 3.158869 -0.025279 -0.090628
H -3.026272 0.545485 0.027381
H -2.352738 0.889968 1.366437
H -0.898789 1.780816 -1.682603
H -1.635462 2.184120 -0.371691
H 1.621509 3.490159 0.264986
H 0.476395 2.525914 -0.235731
H 1.707638 -2.042485 0.521705
H -2.291654 -1.538567 -0.348716
H -1.365403 -1.575556 1.521623

34
o.11h2o, E= -916.268314
O -0.739806 0.042883 -1.454214
O -4.045203 -1.355256 -0.655239
O 0.642144 2.029623 -0.374224
O -1.722149 3.435635 0.254884
O -2.609661 0.859477 0.247033
O -1.354436 -2.251980 -0.199517
O 1.750045 -0.979188 -2.107430
O 1.166251 0.296034 1.693418

O 1.411333 -2.209756 0.416120
O 3.806107 -0.328405 1.172288
O 3.233947 1.090858 -1.091486
H -3.905850 -1.230017 -1.599908
H -3.711567 -0.517849 -0.259909
H -1.124080 -1.484871 -0.793008
H -2.324756 -2.294533 -0.260009
H 1.001092 0.976960 1.000138
H 2.140753 0.217979 1.769110
H 0.841509 -0.601198 -2.056765
H 1.769928 -1.560482 -1.322641
H 0.144390 1.380893 -0.946025
H -0.030347 2.687214 -0.108520
H -1.936652 0.593931 -0.453075
H -2.258899 0.434352 1.067549
H -2.091806 3.813982 -0.548660
H -2.156948 2.549879 0.338435
H 3.531225 -1.216315 0.909927
H 3.728959 0.209669 0.344030
H 2.785869 0.366903 -1.592050
H 2.493649 1.672762 -0.846715
H 0.535714 -2.601582 0.265086
H 1.216463 -1.360424 0.863734
O -1.393405 -0.672445 2.162228
H -0.483746 -0.325749 2.236769
H -1.326230 -1.371347 1.481609

34
o.11h2o, E= -916.266390
O -0.193190 -0.245720 1.518625
O -2.351990 1.478182 1.148559
O 2.264853 -0.700660 2.542075
O 3.559913 0.430708 0.325323
O 1.057837 1.779007 0.134827
O 2.587925 -1.178649 -1.739556
O 0.324478 -2.236810 -0.373786
O 0.732609 0.754268 -2.345994
O -3.387969 -0.526148 -0.354194
O -1.499124 -0.946087 -2.102815
H -2.264591 -0.737592 -1.475464
H -1.877018 -1.393404 -2.864805
H -3.139380 -1.220771 0.294918
H -3.154483 0.310092 0.120475
H -1.570187 0.930948 1.391230
H -1.985412 2.299243 0.768517
H -0.310775 -1.847339 -1.006903
H 0.297340 -1.635555 0.399175
H 1.937851 -1.713862 -1.243874
H 3.072426 -0.675159 -1.048362
H -0.095680 0.239800 -2.349363
H 1.447755 0.074765 -2.294610
H 0.922981 1.427551 -0.784606
H 0.637946 1.109991 0.729481
H 2.849690 1.095722 0.239826
H 3.294637 -0.054055 1.132032
H 2.265511 -0.043480 3.244950
H 1.371491 -0.604062 2.115535
H -1.474179 -1.490534 1.625722
O -2.105585 -2.227096 1.429199
H -1.557761 -2.822170 0.900522
O -0.924114 3.629077 -0.052942
H -0.108508 3.083117 0.072309
H -1.084804 3.586184 -1.001283

34
o.11h2o, E= -916.266202
O -0.317379 0.178316 -1.533202
O -1.090979 -2.444588 -1.704862
O 1.715754 2.061111 -1.547556
O 3.719065 0.259310 -0.708835
O 1.714436 -1.716673 -1.000065
O 2.504231 0.804308 1.741565
O -0.194640 1.385312 0.841114
O 1.436003 -1.763429 1.739475
O -2.803235 -1.720522 0.347942
O -1.191029 -0.821316 2.178112
H -1.836815 -1.209807 1.500040
H -1.644991 -0.812569 3.025075
H -3.077816 -0.890040 -0.108771
H -2.247294 -2.173633 -0.325355
H -0.924552 -1.487922 -1.875450
H -0.201425 -2.765879 -1.497192
H -0.549315 0.677498 1.423739
H -0.268040 1.001091 -0.078158
H 1.652841 1.149964 1.419424
H 3.049311 0.658307 0.935552
H 0.511805 -1.572839 1.980540
H 1.907315 -0.917690 1.906065
H 1.579593 -1.802745 -0.025928
H 1.102100 -1.010689 -1.292742
H 3.212279 -0.559706 -0.885775
H 3.202947 0.946002 -1.173278
H 1.526947 2.426231 -0.675259
H 1.033710 1.354283 -1.646589
H -2.081273 0.518199 -1.312891
O -3.016523 0.638520 -1.024914
H -3.002803 1.454810 -0.493749
O -2.384776 2.996451 0.499625
H -1.554761 2.515941 0.730791
H -2.117186 3.646792 -0.157281

34
o.11h2o, E= -916.265120
O -0.781409 -0.313183 1.243564
O 1.479695 -0.835145 2.131645
O 3.150900 0.849270 1.084651
O -0.632770 -1.985799 -0.990535
O 3.110484 -0.555434 -1.106419
O 0.646388 0.077142 -2.311394
O 1.770765 3.124888 0.062454
O -0.522364 1.782991 -0.412245
O -3.342357 -1.223835 -0.858199
O -3.259934 1.595529 -0.711939
O -3.307292 0.100055 1.720334
H -3.576109 -0.054073 2.629505
H -2.314571 -0.044093 1.683528
H -3.451518 1.423988 0.225172
H -3.293494 0.688666 -1.079434
H -2.458724 -1.633363 -0.939509
H -3.464993 -1.068926 0.092275
H -1.471268 1.922568 -0.629102
H -0.552338 1.068854 0.277940
H -0.800984 -0.968203 0.512281
H 0.115516 -0.516106 1.767344

H 2.564284 0.272078 1.670615
H 2.662707 1.678235 0.915518
H 0.147381 -2.436958 -0.583632
H -0.233285 -1.315334 -1.592327
H 2.156965 3.081492 -0.818205
H 0.858836 2.768893 -0.062869
H 0.264325 0.769156 -1.732961
H 1.547968 -0.086423 -1.972980
H 3.178597 0.005895 -0.265108
H 3.987650 -0.572261 -1.498107
O 1.710235 -2.697934 0.227240
H 1.651426 -2.185600 1.079799
H 2.302915 -2.146860 -0.313005

34
o.11h2o, E= -916.264626
O -2.411144 -1.952728 -0.844355
O -4.075672 0.317528 -1.348155
O -2.068003 1.117061 0.279152
O -0.134069 -0.226121 -1.068073
O 1.091480 2.179790 -1.084059
O 3.641833 1.134166 -0.664582
O 2.465908 -1.188975 -1.474995
O 2.170455 -3.269442 0.471645
O 0.443323 -1.336753 1.291404
O -2.220423 -1.237973 1.852404
O -0.456701 3.264163 0.967566
H -1.578938 -1.454272 -0.976816
H -3.105224 -1.320999 -1.112346
H -3.834216 0.702148 -2.196595
H -3.477830 0.760943 -0.700503
H -1.380012 0.669316 -0.280973
H -1.659718 1.964162 0.564018
H 1.528485 -0.893100 -1.439672
H 2.513727 -1.950052 -0.866707
H 1.564619 -2.659856 0.953313
H 1.585370 -3.921768 0.073409
H -0.452914 -1.391448 1.704132
H 0.234952 -1.102377 0.353053
H 0.099121 3.128042 0.172280
H 0.059853 2.820645 1.657518
H -2.279082 -0.304528 1.571369
H -2.436682 -1.703854 1.017187
H 0.631911 1.328637 -1.295406
H 2.051068 1.986806 -1.149208
H 3.354047 0.241463 -0.975468
H 3.469100 1.117798 0.287246
H 1.412655 1.483874 0.731540
O 1.608586 1.222490 1.647025
H 1.255109 0.312770 1.708811

34
o.11h2o, E= -916.264567
O -1.031465 -1.435789 0.766015
O 1.175906 -1.102797 2.321117
O -3.599174 -0.293477 0.890721
O -2.854055 1.177497 -1.387631
O -1.350268 -1.150142 -2.077263
O -0.626775 2.010468 -0.022485
O -1.206813 0.698337 2.390632
O 0.801326 0.580680 -2.019483
O 1.995464 2.822502 -0.351728
O 3.046848 0.640434 1.120654
O 2.822666 -1.008785 -1.098979
H 3.041523 -0.508430 -0.282426
H 2.240196 -1.748798 -0.834985
H 2.073941 2.415573 -1.225305
H 1.032370 2.769497 -0.184200
H 2.701989 1.462328 0.715107
H 2.307905 0.226833 1.604122
H 0.407651 -1.323771 1.731171
H 1.759599 -1.867003 2.281325
H -0.450355 0.623637 2.984073
H -1.191501 -0.158633 1.886682
H -0.816394 1.656945 0.869632
H -1.450734 1.867906 -0.549754
H 1.573037 0.032976 -1.734657
H 0.418206 0.973022 -1.212270
H -0.549572 -0.589623 -2.196175
H -1.368093 -1.348573 -1.121264
H -2.450333 0.418795 -1.854731
H -3.314534 0.751017 -0.637198
H -3.376825 0.256208 1.652587
H -2.789703 -0.840257 0.773612
H 0.124771 -2.458857 -0.085270
O 0.788143 -2.910074 -0.662072
H 0.382431 -2.841626 -1.536055

34
o.11h2o, E= -916.264322
O 0.312384 -0.093476 0.947673
O 2.194920 -1.436836 -0.416076
O -1.314356 -2.416792 1.340716
O -0.024217 -3.127376 -1.097922
O 2.164214 1.723210 1.035123
O 2.226331 1.107823 -1.747512
O -1.224032 -0.590499 -1.335044
O -0.925433 2.351533 0.646391
O -0.438084 1.893966 -2.095561
O -3.636325 1.661377 0.955126
O -3.459205 -0.959408 0.265257
H 1.501697 -1.050675 0.180733
H 3.024052 -1.254904 0.059376
H 2.352067 1.492003 -0.860917
H 2.210621 0.150209 -1.559350
H -0.574461 1.464129 0.881916
H -1.889595 2.259325 0.792647
H -0.814107 -0.463788 -0.454695
H -1.022631 0.258907 -1.800073
H -0.757292 -1.615671 1.368136
H -0.944261 -2.892561 0.569425
H 1.435757 1.019114 1.078805
H 1.713466 2.555713 1.214109
H -0.455337 -2.336363 -1.473123
H 0.886194 -2.827295 -0.935245
H -4.165277 1.972323 0.214106
H -3.582943 0.679455 0.819355
H -2.887932 -0.826929 -0.517758
H -2.897098 -1.555861 0.804535
H 0.522652 1.694713 -2.104963
H -0.615460 2.225469 -1.189484
O 4.078426 -0.232696 1.420847
H 3.739749 -0.599010 2.244322
H 3.583164 0.605092 1.315996

34
o.11h2o, E= -916.263807
O -1.476179 1.971545 -1.787319
O -2.880220 -0.352042 -1.068257
O -1.151113 -2.436245 -0.768129
O 1.067872 -1.796986 -1.820310
O 2.907673 -1.306350 0.035274
O -2.590127 0.103468 1.362121
O -1.327052 2.387682 0.932700
O 1.048726 1.923326 1.770363
O 1.351800 -0.792926 2.383615
O -1.108747 -2.061893 1.893376
O 2.824577 1.526278 -0.328303
O 1.064882 1.007357 -2.446204
H -0.585636 1.674781 -2.075392
H -3.801431 -0.492693 -1.303460
H -2.001766 1.153030 -1.680496
H 1.188615 -2.375882 -2.577686
H 0.165537 -2.055740 -1.388021
H 1.912796 -1.028482 1.624824
H 1.190684 0.165751 2.271867
H 1.011033 0.046580 -2.301973
H 1.701256 1.314341 -1.765281
H -0.255835 -1.649815 2.147051
H -1.763107 -1.314705 1.883023
H 2.225756 1.760065 0.411826
H 3.053087 0.595389 -0.169292
H -2.846326 -0.144978 -0.007267
H -1.363811 2.380461 -0.053958
H -1.902235 1.612930 1.201950
H 1.218315 2.585060 2.446349
H 0.101553 2.108638 1.422703
H 2.256324 -1.555990 -0.673228
H 3.563534 -2.007229 0.074189
H -1.825354 -1.748946 -0.987413
H -1.099725 -2.408276 0.228280

34
o.11h2o, E= -916.263335
O -1.025169 -1.457622 -2.156941
O 1.208559 -2.193290 -1.194128
O 1.101411 -2.136424 1.519666
O 2.578816 -0.168071 1.427280
O -1.382581 -1.195985 2.259158
O -2.882440 -1.328603 -0.214472
O 2.996345 -0.159677 -1.193111
O -1.268910 1.606976 2.123673
O -2.781022 1.553266 -0.106523
O -1.032622 1.375770 -2.162560
O 1.372338 2.158367 -1.442559
O 1.336479 2.215510 1.434594
H -0.116681 -1.752499 -1.765949
H -1.127396 -1.917461 -2.994497
H -3.470328 -2.087883 -0.245773
H -2.233100 -1.438560 -0.953084
H 1.483229 -2.843411 2.047214
H -0.488854 -1.550019 2.049888
H 1.873618 1.403392 1.615711
H 1.359280 2.299614 0.465428
H 1.144549 -2.305110 -0.212951
H 1.894002 -1.481909 -1.307653
H -0.337672 1.858100 1.919945
H 2.519268 0.667457 -1.389922
H 3.062529 -0.162839 -0.197426
H -3.063749 0.626027 -0.074271
H -2.242313 1.670342 0.723080
H -1.010279 0.405426 -2.258109
H -1.686188 1.537417 -1.431912
H 1.563882 2.955555 -1.943658
H 0.447937 1.873058 -1.712257
H 1.795782 -1.277571 1.562958
H -1.889538 -1.286467 1.435437
H -1.247614 0.645602 2.315178

34
o.11h2o, E= -916.261877
O 1.712667 -2.581924 1.337960
O 3.897907 -1.143784 0.268830
O 2.021005 0.938001 0.968539
O -0.262657 -0.470049 1.286234
O -1.134403 1.910368 0.239751
O -3.880997 1.518533 0.542254
O -2.996658 -0.984438 0.839477
O -2.654228 -2.643115 -1.422688
O -0.089912 -2.034188 -0.886487
O 1.893050 -0.204192 -1.513521
O 1.123443 3.508975 0.402016
H 1.125117 -1.830973 1.564249
H 1.302066 -2.899878 0.520521
H 3.270336 -1.769343 0.685072
H 3.591540 -0.293851 0.625891
H 1.208700 0.491523 1.314936
H 1.815648 1.896682 0.901920
H -2.042623 -0.839628 1.037709
H -3.012481 -1.553589 0.044199
H -1.693714 -2.409036 -1.423094
H -2.675195 -3.565774 -1.150908
H 0.569342 -1.434313 -1.302242
H -0.282674 -1.583674 -0.026494
H 1.194770 3.347934 -0.557535
H 0.210614 3.200298 0.563908
H 1.856539 0.297957 -0.662543
H 2.725442 -0.691501 -1.380571
H -0.875715 1.082818 0.715859
H -2.113083 1.930946 0.267338
H -3.659957 0.549967 0.605749
H -4.150149 1.766583 1.431777
O 0.477896 2.285235 -2.077474
H -0.217167 2.033577 -1.437868
H 0.995759 1.477895 -2.216995

34
o.11h2o, E= -916.261304
O 1.461358 -0.222412 -2.620273
O 3.299602 1.079452 -0.889756
O 1.200931 1.590897 0.899983
O -0.369496 0.078383 -0.610773
O 0.380495 -1.926483 1.000324
O -1.909865 -3.487596 0.751937
O -2.621567 -1.477694 -0.848756
O -4.040050 0.736856 0.125288
O -1.990528 2.251027 -0.768021
O -0.450031 3.701821 1.100468

O 2.304232 -0.541768 2.311874
H 0.596393 -0.050328 -2.203546
H 2.056724 0.447077 -2.229110
H 3.364914 0.147876 -0.618582
H 2.642126 1.448541 -0.265809
H 0.597573 1.015166 0.338209
H 1.568840 0.984117 1.577350
H -1.774681 -0.981959 -0.941337
H -3.262377 -0.797012 -0.556137
H -3.360843 1.375336 -0.217194
H -3.875633 0.700504 1.072850
H -1.563184 2.831161 -0.105986
H -1.328794 1.542188 -0.938860
H 1.545389 -1.122665 2.079615
H 2.952082 -0.767128 1.631292
H 0.200800 2.959109 1.177014
H 0.023736 4.389950 0.623348
H 0.028285 -1.188496 0.427773
H -0.343288 -2.583723 1.059842
H -2.284649 -2.752976 0.191412
H -1.763682 -4.210456 0.134280
H 1.941068 -2.038113 -0.009347
O 2.704199 -1.767486 -0.557135
H 2.284965 -1.468144 -1.388347

34
o.11h2o, E= -916.260402
O 0.399931 -0.282551 -0.790548
O 2.289487 0.754911 0.872753
O -0.347289 2.509089 -1.095637
O 0.399042 2.721985 1.624365
O 2.706712 -1.538008 -1.546659
O 1.356392 -1.991688 1.149251
O -1.261760 0.492050 1.332414
O -1.755027 -2.134569 -0.725169
O -1.432855 -2.175988 2.017996
O -3.892289 -0.513506 -1.542461
O -2.963717 1.767524 -0.449797
H 1.661825 0.617491 0.129746
H 3.166122 0.750027 0.437468
H 0.830795 -1.726301 0.371886
H 1.790786 -1.153058 1.390348
H -1.007374 -1.520800 -0.874647
H -2.542151 -1.645676 -1.045110
H -0.757809 0.240552 0.525740
H -1.421698 -0.367665 1.781688
H -0.032679 1.586012 -1.129090
H -0.077802 2.798647 -0.198581
H 1.838904 -1.066056 -1.439990
H 2.694472 -2.165297 -0.811523
H -0.252357 2.014112 1.793975
H 1.234397 2.228266 1.543877
H -4.686495 -0.656235 -1.018993
H -3.553112 0.371562 -1.242966
H -2.580723 1.358442 0.352428
H -2.166282 2.169783 -0.856979
H -0.470936 -2.310490 2.032168
H -1.668607 -2.312454 1.070810
O 4.459985 0.400158 -0.855653
H 4.421305 1.067697 -1.547537
H 3.910537 -0.353818 -1.191555

19
oh2.6h2o, E= -533.967377
O 0.285834 -0.443151 -1.587703
O 2.028998 1.237601 -0.475929
O -1.882484 -1.502281 -0.081664
O -0.159188 -0.733878 2.046989
O 1.674403 -1.815175 0.038469
O -1.777207 1.231784 -0.957751
O -0.141408 1.936260 1.198000
H 0.405310 -0.654263 -2.517857
H -1.168388 -1.313370 -0.724689
H -1.442491 -1.325663 0.774171
H -0.168937 0.231340 1.858089
H 0.583238 -1.079924 1.521039
H 1.075652 -1.353279 -0.684979
H -0.997470 0.751105 -1.334628
H -2.259605 0.483123 -0.568307
H 1.385844 0.684816 -1.010041
H 2.491798 0.578869 0.054035
H 0.690537 1.873464 0.687752
H -0.827067 1.834501 0.502125

19
oh2.6h2o, E= -533.966040
O 0.249376 0.785340 -1.403067
O -1.358657 2.183304 0.026048
O 2.331372 1.166696 0.182482
O -1.680394 -0.200620 1.624283
O 1.053164 -0.854774 1.729865
O -1.817639 -1.165450 -1.037599
O 0.967179 -1.784249 -0.918195
H -1.240927 -0.429003 -1.325946
H -1.931368 -0.975068 -0.083799
H 0.846076 -0.837751 -1.191917
H 0.041267 -2.079535 -0.956659
H 1.547337 1.109712 -0.446974
H 2.929415 0.483120 -0.137052
H -1.629291 0.696893 1.245359
H -0.743448 -0.447392 1.782168
H 1.484390 -0.021404 1.461017
H 1.056906 -1.357089 0.886665
H -0.695279 1.661466 -0.581822
H 0.379717 1.154069 -2.281566

19
oh2.6h2o, E= -533.964251
O -0.050762 1.406585 0.905680
O 2.527160 1.438951 -0.110267
O 2.107757 -1.497269 -0.048949
O 0.240348 -0.152598 -1.597189
O -0.300047 -1.248264 1.552830
O -1.957957 -1.057335 -0.523924
H 1.649712 1.583088 0.306987
H -0.113686 0.497245 1.292305
H 0.535809 -1.540522 1.147335
H -0.979437 -1.328589 0.824458
H -2.585851 -1.715648 -0.829892
H -2.467760 0.256697 -0.291478
H 1.473847 -1.178123 -0.731262
H 2.527549 -0.667779 0.227416
H -0.618450 -0.631638 -1.385687
H 0.196129 0.551644 -0.929816

H 2.304878 1.178911 -1.011902
H -0.964494 1.587582 0.595305
O -2.686281 1.285821 -0.079902

19
oh2.6h2o, E= -533.963373
O -0.682912 -1.518390 -1.060884
O 1.953792 -1.770954 0.035739
O 2.920787 0.855968 -0.341262
O 0.701320 1.673783 0.480992
O -0.315817 -0.471240 1.535791
O -2.997707 -0.066490 0.469669
O -1.310509 1.291289 -1.212958
H -0.631643 -1.188695 -0.137747
H 0.231754 -1.823849 -1.176892
H 1.322246 -1.551713 0.737968
H 2.373671 -0.907908 -0.171287
H 1.995960 1.270689 -0.009334
H 0.085542 0.407901 1.228078
H -1.273096 -0.309327 1.565559
H -2.497674 0.568402 -0.103571
H -2.888224 -0.899564 -0.004242
H -1.025779 0.419800 -1.537342
H -0.553131 1.558834 -0.613000
H 0.708751 2.503701 0.965122

19
oh2.6h2o, E= -533.962901
O 0.648718 -0.847015 -0.964572
O -1.447946 1.038290 -1.249956
O -0.290680 1.846746 1.254236
O -0.598032 -0.984520 1.283242
O 2.907776 -1.435791 0.143964
O -3.144340 -0.970089 -0.108639
O 2.017310 1.405720 -0.352316
H 0.383821 -1.449417 -1.665284
H -0.079897 -1.566656 1.845845
H -0.138781 -1.004230 0.369691
H 0.571142 1.845388 0.792794
H -0.439528 0.905030 1.463358
H -1.250080 1.542606 -0.439006
H -0.709289 0.392738 -1.274960
H 2.006988 -1.278107 -0.341948
H -2.443918 -1.150734 0.537779
H -2.753226 -0.232352 -0.614528
H 1.476732 0.741768 -0.839245
H 2.633585 0.827251 0.117819

19
oh2.6h2o, E= -533.962728
O -1.263786 0.366952 -1.291750
O -2.054633 -1.939783 0.119188
O -1.834463 0.621696 1.436108
O 0.141972 2.444354 -0.842095
O 3.421225 -0.787231 0.009982
O 0.668663 -1.294073 -0.638805
O 1.086692 0.773881 1.261051
H -1.935962 -1.225938 -0.545486
H -2.104974 -1.401724 0.926343
H 0.032460 -0.658156 -1.081889
H 0.052716 -1.924625 -0.233615
H -0.466324 1.624837 -1.080279
H 3.106925 -0.097861 0.610005
H 2.588816 -1.113330 -0.373465
H -1.817257 0.681476 0.456816
H -0.895931 0.723843 1.669644
H 0.906709 1.491339 0.620228
H 0.951013 -0.032224 0.723037
H -1.743553 0.445977 -2.120759

19
oh2.6h2o, E= -533.962717
O 0.911053 -0.682064 -1.230725
O 0.932663 0.291005 1.458617
O -0.504601 -2.209790 0.771850
O -2.722504 -0.771258 -0.120339
O -1.146685 0.958314 -1.230587
O -0.830813 2.428517 0.783930
O 3.511529 0.074361 -0.324402
H 1.023173 0.063208 0.513417
H 0.335617 1.065590 1.438119
H 2.718819 -0.220674 -0.810875
H 3.146398 0.323674 0.533541
H -0.087730 -1.507887 1.299048
H -1.353188 -1.811278 0.478771
H 0.147514 -0.042502 -1.369881
H -1.453303 1.360797 -2.047390
H -3.058311 -0.277354 0.633948
H -2.155835 -0.093806 -0.627801
H -0.973762 1.880742 -0.112907
H 0.505476 -1.453186 -0.794748

19
oh2.6h2o, E= -533.962661
O -0.940746 -0.264723 -1.130549
O -2.631472 -1.896489 0.048280
O -1.453696 2.188197 -0.241957
O 2.893133 -1.230737 -0.394391
O 1.414824 1.239373 -0.837286
O 0.329607 1.245906 1.811489
O 0.273716 -1.431404 0.869743
H -0.936431 -0.487276 -2.065546
H 2.155092 -1.552373 0.145914
H 2.587046 -0.343931 -0.655640
H -1.415983 1.273341 -0.641618
H -0.647468 2.588526 -0.590282
H 0.625629 0.691999 -1.055777
H 1.309508 1.377387 0.123168
H -2.067564 -1.196844 -0.443395
H -0.070400 -1.049623 0.006771
H -0.477473 -1.958968 1.167720
H -0.449083 1.579214 1.326378
H 0.304199 0.277557 1.679675

19
oh2.6h2o, E= -533.961780
O 0.218238 0.438825 1.341638
O 1.466895 1.609745 -0.713008
O -1.652164 1.673360 -0.190720
O 1.220997 -1.164862 -1.571351
O 2.810842 -0.563528 0.790212
O -0.821929 -1.643778 0.395421
O -3.415554 -0.573628 -0.152317
H -1.031669 1.263371 0.467196

H -1.077460 1.858825 -0.941936
H -2.918660 0.259264 -0.260446
H -2.741394 -1.194095 0.165662
H 0.997715 1.336099 0.118266
H 2.363520 1.299439 -0.525743
H 1.961689 -0.250256 1.169515
H 2.510990 -1.011042 -0.019701
H -0.411129 -0.805610 0.892078
H 0.484046 -1.479124 -1.013517
H 1.166620 -0.194545 -1.503240
H 0.077144 0.708599 2.252862

19
oh2.6h2o, E= -533.961474
O -1.785704 -0.038295 -0.258433
O 0.255972 1.135351 0.713049
O 0.711412 0.385318 -1.933167
O 2.930322 1.082046 0.010040
O 1.578150 -1.003465 1.610641
O -4.341599 0.442870 0.462975
O 0.211691 -2.099001 -0.569834
H -1.597473 0.143636 -1.188063
H -3.387038 0.253557 0.214914
H -1.015607 0.459756 0.229454
H 0.030695 1.934183 1.197124
H -0.632469 -1.657770 -0.369995
H 0.761677 -1.885288 0.215754
H 2.400974 -0.671858 1.221222
H 0.986387 -0.211897 1.480859
H 0.548755 0.870519 -1.096192
H 0.667851 -0.546688 -1.642192
H 2.733585 0.811150 -0.897204
H 2.020714 1.262109 0.352149

19
oh2.6h2o, E= -533.960430
O 2.387289 -0.111670 -0.114109
O 0.500421 -0.943681 1.824262
O -0.906725 0.861468 0.152899
O -3.568400 1.144888 -0.028683
O -0.701329 -2.595570 -0.164242
O 0.380711 -0.677564 -1.511486
O 1.459094 2.601227 0.019843
H 3.137473 -0.668117 -0.337628
H 1.998694 1.793901 -0.020284
H 0.558327 2.239434 0.073729
H -0.567198 0.390365 0.940565
H 0.125987 -1.659744 1.272045
H 1.288453 -0.658213 1.320333
H -1.599414 -2.263593 -0.063476
H -0.277268 -1.935302 -0.808190
H 0.350420 -0.526772 -2.458413
H -0.482767 0.341686 -0.605051
H -2.567395 1.060816 0.048388
H 1.626187 -0.347245 -0.789896

19
oh2.6h2o, E= -533.960021
O -1.858601 -1.405908 -0.143919
O 0.205402 -0.797750 1.307977
O -0.337121 1.706475 0.733312
O 1.701142 2.543393 -0.691178
O 0.828843 -2.567094 -0.730685
O 2.519654 -0.266183 -0.093423
O -2.781044 1.045816 -0.614751
H -2.459730 0.101997 -0.560936
H -3.451938 1.100444 0.072521
H -1.169217 1.633704 0.229732
H -0.123026 0.760823 1.023519
H 0.254665 -1.001832 2.245128
H -1.148962 -1.176327 0.544555
H 0.613759 -2.157986 0.137769
H 1.525563 -1.960552 -1.025190
H 1.760619 -0.421199 0.520168
H 2.411019 0.653787 -0.379472
H 0.886655 2.266729 -0.140697
H -1.325604 -1.869591 -0.805753

19
oh2.6h2o, E= -533.959824
O -1.895180 -0.775908 -0.117485
O 0.193468 -0.636130 1.813511
O 1.136403 1.717271 0.566052
O 3.739335 0.268574 -0.205940
O 1.662155 -1.361776 -0.507011
O -0.097764 0.376649 -1.364081
O -4.355323 0.437081 -0.063936
H -1.862530 -1.690782 -0.412345
H -3.501210 -0.053261 -0.124164
H -4.122386 1.217107 0.448323
H -1.178844 -0.252164 -0.724815
H -0.270445 0.718362 -2.244010
H -0.641807 -0.706668 1.309859
H 0.521365 0.258493 1.578969
H 2.059656 1.478460 0.397998
H 0.646959 1.350183 -0.226317
H 1.032818 -0.759513 -1.010347
H 1.256506 -1.353039 0.384368
H 2.995163 -0.413278 -0.346391

19
oh2.6h2o, E= -533.959099
O 0.467712 0.542493 -1.350872
O -0.479026 1.214087 1.028331
O 2.093938 -1.206161 -0.369680
O -3.277572 0.771728 0.142304
O -1.550412 -1.223511 -0.922732
O 2.398169 1.629766 0.271350
O 0.255201 -1.924483 1.395237
H -0.832421 -0.591287 -1.196761
H -1.173660 -1.652192 -0.140703
H -2.840523 0.016030 -0.298204
H -2.518477 1.252681 0.504556
H 1.485848 -0.642767 -0.948736
H 2.621018 -0.510905 0.055531
H 1.796066 1.390535 -0.475947
H 1.755807 1.707882 0.990494
H -0.217210 1.056334 0.073824
H -0.683266 0.330546 1.357577
H 1.008136 -1.729923 0.740276
H 0.334599 0.941720 -2.213410

19
oh2.6h2o, E= -533.958721

O 2.968331 1.627495 -0.001732
O 2.439256 -0.969143 -0.245037
O 0.021151 -1.351806 1.529399
O -2.497285 -1.114257 -0.259714
O -2.820272 1.742343 0.006680
O 0.000686 1.249336 0.266241
O 0.043742 -0.913223 -1.212625
H 2.831673 0.627203 -0.121060
H 2.173146 -1.273427 0.633392
H 1.543416 -0.981902 -0.741928
H 0.001365 -0.380117 1.446226
H -2.239734 -1.276024 0.656298
H -1.614691 -1.056406 -0.716361
H -1.862771 1.838071 0.143470
H -2.904265 0.783841 -0.147722
H 0.881391 1.647269 0.204939
H -0.013759 0.543686 -0.449054
H -0.017081 -1.028905 -2.163845
H -0.023556 -1.609237 0.589943

22
oh2.7h2o, E= -610.430495
O -0.000296 -1.111203 -1.407808
O 0.000663 1.389979 -1.630650
O 2.451522 -1.155156 -0.244019
O -2.452286 -1.154084 -0.244296
O 2.139222 1.582279 0.163326
O -2.138386 1.583261 0.163255
O -0.000576 -1.802946 1.359986
O 0.000183 0.923026 1.904827
H -0.000255 -1.649486 -2.204461
H 1.623853 -1.165098 -0.795874
H 2.129702 -1.526584 0.589871
H 0.000265 0.317967 -1.613481
H -1.624536 -1.164370 -0.796020
H -2.130773 -1.525666 0.589642
H -0.000483 -1.738973 0.385875
H -0.000267 -0.861057 1.649051
H -2.416501 0.647488 0.049307
H -1.573108 1.739516 -0.615049
H -0.776246 1.249928 1.405083
H 0.776677 1.249652 1.405015
H 2.416672 0.646291 0.049404
H 1.574637 1.739157 -0.615343

22
oh2.7h2o, E= -610.429141
O 2.113024 1.610768 -0.088473
O 0.006673 1.121333 -1.807083
O 0.190549 -1.675276 -1.375045
O 2.533492 -1.164210 0.231209
O 0.050001 1.170383 1.757206
O -2.107599 1.627625 -0.018651
O -2.551766 -1.139734 0.029222
O -0.188231 -1.278528 1.262953
H 1.627484 1.684716 0.750265
H 1.401715 1.528122 -0.767995
H -0.782703 1.397615 -1.295159
H 0.005042 0.142815 -1.758738
H -0.003743 -1.651295 -0.403231
H 1.161033 -1.677991 -1.350699
H 2.583661 -0.199030 0.075708
H 1.699959 -1.252943 0.732718
H -2.440644 0.705337 -0.018591
H -1.487039 1.641265 0.734814
H -2.234922 -1.433619 -0.833104
H -1.743396 -1.243028 0.608921
H -0.121308 -1.934903 1.961381
H -0.034276 0.114056 1.633005

22
oh2.7h2o, E= -610.428299
O 0.423098 -1.396250 -1.049593
O 0.402068 1.031568 -1.792890
O 2.728769 -1.178033 0.087138
O -2.313153 -1.211825 -0.539177
O 1.967350 1.559750 0.479310
O -2.011417 1.566398 -0.431069
O -0.592913 -1.574443 1.673014
O -0.455960 1.206106 1.911259
H 0.466945 -2.122486 -1.678813
H 1.798918 -1.323475 -0.345803
H 0.409669 0.018177 -1.582497
H 0.647016 1.131310 -2.716070
H -1.392915 -1.285744 -0.884273
H -2.146524 -1.420236 0.398257
H -0.108687 -1.659391 0.831517
H -0.528514 -0.615510 1.881701
H -2.309851 0.632370 -0.476193
H -1.222971 1.561503 -1.005006
H -1.071901 1.435232 1.184149
H 0.429831 1.404631 1.545143
H 2.357027 0.665842 0.516429
H 1.489223 1.551606 -0.372474

22
oh2.7h2o, E= -610.427913
O 0.229056 -1.410441 -1.441044
O -1.685253 -1.460227 0.199564
O 0.703834 1.356041 -1.658056
O 2.356050 -1.511414 0.392421
O -3.391305 0.385935 -0.051959
O 0.124633 -0.294670 1.798817
O -1.016690 1.923954 0.429620
O 2.520471 1.303083 0.399032
H -0.001037 -1.901751 -2.233639
H -0.588758 -1.476694 -0.794604
H -1.990687 -2.329548 0.472576
H 0.534469 0.392808 -1.698374
H 0.008669 1.684612 -1.044486
H 1.707617 -1.609053 -0.331943
H 2.657101 -0.584627 0.315808
H -0.599842 -0.783488 1.319508
H 0.928594 -0.766614 1.512335
H -0.575796 1.258555 0.995647
H -1.894456 1.535614 0.244670
H 1.988220 1.414438 -0.425874
H 1.841604 1.262878 1.085590
H -2.742075 -0.435215 0.035632

22
oh2.7h2o, E= -610.427423
O 0.155527 -0.268291 -1.607748
O -1.361413 -1.495210 -0.099480

O 2.441435 1.001330 -0.807151
O -1.263634 1.815619 -0.554178
O -3.558610 -0.067344 0.121664
O 2.048511 -1.821741 -0.153661
O 0.825437 1.850819 1.336638
O 0.484913 -0.836503 1.991072
H 0.012404 -0.392367 -2.550114
H 1.607386 0.678089 -1.228329
H 2.799312 0.166919 -0.465178
H 1.426561 -1.425995 -0.796671
H 1.647591 -1.571589 0.704523
H -0.710419 -1.006546 -0.811355
H -1.212107 -2.442159 -0.165921
H -0.756080 1.113098 -1.034445
H -2.090100 1.384128 -0.291064
H 0.016971 1.977129 0.798023
H 1.515716 1.692968 0.658956
H 0.622883 0.132911 1.888471
H -0.277164 -1.027432 1.414860
H -2.780277 -0.708579 0.060994

22
oh2.7h2o, E= -610.426552
O 0.209398 -1.240666 1.709642
O -1.990171 -1.389450 0.075591
O -0.268015 -0.809425 -1.993472
O 1.960151 -1.574035 -0.479527
O 2.617105 1.168538 -0.384405
O 0.775563 1.147250 1.470352
O -0.456912 1.719255 -0.803893
O -3.179606 0.983234 0.153053
H -1.299526 -1.424544 0.774853
H -1.457127 -1.315851 -0.752636
H 0.437201 -0.189810 1.704380
H 0.666959 1.774077 2.188912
H 1.414702 -1.593547 0.332903
H 2.353668 -0.675993 -0.474952
H 2.033533 1.499813 -1.077647
H 2.034314 1.236543 0.431432
H 0.553250 -1.181317 -1.604718
H -0.269605 0.126052 -1.692325
H -0.052264 1.555623 0.099631
H -1.413884 1.686224 -0.648266
H -2.766887 0.052583 0.161265
H 0.425552 -1.587458 2.578437

22
oh2.7h2o, E= -610.425636
O -0.459318 -1.306593 -1.619835
O 0.454017 -2.239913 0.675187
O 2.399418 -0.052366 0.541831
O 1.345952 0.429333 -1.777703
O -0.521955 2.337546 -0.663145
O 0.359059 1.488707 1.673455
O -1.390691 -0.557178 1.887464
O -2.407719 0.106150 -0.614095
H -0.630114 -1.880266 -2.370559
H 0.095384 -2.007214 -0.235335
H 3.355243 0.029144 0.495463
H 2.045561 0.140800 -0.392339
H 0.600842 -0.313727 -1.809202
H 0.861010 1.264052 -1.689340
H -0.186672 2.093346 0.267688
H -1.901051 -0.338654 1.074004
H -2.098270 1.013870 -0.743598
H -1.713801 -0.470850 -1.077024
H -0.300280 0.772577 1.885539
H 1.175229 1.004661 1.438751
H -0.779528 -1.267349 1.580777
H 1.246347 -1.685876 0.749896

22
oh2.7h2o, E= -610.425035
O 0.157363 -0.154279 -1.704503
O -1.193921 1.558121 -0.364097
O -1.198669 -1.877996 -0.194719
O 1.917823 -1.766458 -0.551087
O -3.393196 0.192344 0.314480
O 1.605894 2.030494 -0.588991
O -0.444139 -0.108471 1.871787
O 2.334427 0.185106 1.439516
H 0.047524 -0.210049 -2.657016
H 1.350024 -1.141089 -1.089280
H 1.260364 -2.345736 -0.148432
H -0.739463 0.877697 -0.980205
H -0.476248 2.198135 -0.227391
H -0.739763 -1.285511 -0.859442
H -2.089975 -1.506914 -0.120649
H 1.273749 1.298431 -1.148667
H 1.983197 1.550947 0.176961
H 2.378713 -0.536291 0.778072
H 1.418463 0.103845 1.773973
H -0.691236 0.602197 1.252452
H -0.620945 -0.902878 1.328560
H -2.639060 0.826316 0.141975

22
oh2.7h2o, E= -610.423110
O 0.822569 1.959700 0.078505
O -1.978465 1.739805 -0.000476
O -2.371779 -0.564862 -1.594348
O -2.280331 -0.591044 1.674494
O -0.519481 -1.514484 0.057885
O 1.344674 -0.307688 1.677615
O 3.580755 -0.352650 -0.177102
O 1.278262 -0.242918 -1.589217
H 0.988810 1.299165 0.784066
H -0.154964 2.029836 0.042554
H -2.101190 1.147019 0.764150
H -2.100479 1.121889 -0.748660
H -1.626021 -0.987482 -1.073457
H -3.164756 -0.855036 -1.132042
H 0.684603 -0.814750 1.143399
H 2.194128 -0.480663 1.236307
H 2.807237 -0.421759 -0.797758
H 3.694723 0.598926 -0.079152
H 1.090752 0.649751 -1.228238
H 0.633277 -0.803072 -1.092341
H -1.530068 -1.019225 1.096378
H -0.425690 -2.471479 0.065952

22
oh2.7h2o, E= -610.423057
O 1.840638 -0.223236 0.104745

O 4.498119 0.086953 -0.123016
O 0.031362 0.509322 2.097233
O -0.770958 2.317818 0.156973
O -2.794662 0.514055 -0.875879
O -0.128516 0.397880 -1.614619
O -1.881298 -1.406792 1.125865
O -0.218456 -2.061331 -0.810398
H 1.235029 0.108283 -0.616837
H 1.361111 0.009406 0.930258
H -0.651832 -0.165750 1.939888
H -0.255721 1.265510 1.534339
H -0.381636 1.741561 -0.555691
H -1.709793 2.094857 0.053695
H -1.905885 0.448585 -1.303268
H -2.731015 -0.126017 -0.149729
H -1.269270 -1.741911 0.401541
H -2.294257 -2.183025 1.513204
H -0.263280 -1.163853 -1.287960
H 0.656315 -1.983577 -0.399093
H 3.501196 -0.001897 -0.026831
H 0.099199 0.620479 -2.520747

22
oh2.7h2o, E= -610.422801
O 0.715955 -1.403694 -0.774898
O 0.602448 -0.315703 1.701127
O 0.015473 0.997682 -1.752822
O 3.166028 -0.570891 0.061411
O -1.891942 -1.726443 -0.733926
O -2.144828 -0.219050 1.565065
O 1.728535 1.878749 0.271851
O -2.450542 1.695707 -0.269521
H 0.991472 -2.200896 -1.236850
H 0.275241 0.068127 -1.508837
H -0.848803 1.163094 -1.342301
H 2.316869 -0.878134 -0.360927
H 2.972964 -0.701227 0.997898
H 0.634724 -0.881152 0.889837
H 0.915040 0.552514 1.371838
H -0.880861 -1.649223 -0.736447
H -2.170275 -1.095220 -1.405774
H -2.182455 -0.907814 0.863847
H -1.187344 -0.209967 1.802792
H -2.347295 1.059404 0.514012
H 2.447668 1.241904 0.104246
H 1.134044 1.747725 -0.499629

22
oh2.7h2o, E= -610.422528
O 0.210221 0.855702 -1.220389
O 2.149015 -0.356559 0.106817
O -0.398080 -1.619833 -1.027510
O -0.572427 2.029879 1.063034
O 4.819107 -0.016326 -0.059706
O -0.337739 -0.645265 1.772883
O -2.851012 -1.449383 0.301374
O -2.572523 1.213982 -0.827772
H -1.645534 1.082763 -1.125762
H -2.843610 0.338910 -0.497124
H -1.494939 2.031555 0.757007
H -0.113804 1.779320 0.219160
H -0.196558 -0.643642 -1.256628
H 0.476079 -2.000928 -0.887189
H 1.507929 0.196063 -0.439116
H 1.715499 -0.443396 0.967788
H -0.435366 0.330107 1.671902
H -0.338759 -0.973681 0.857597
H -2.420278 -1.225557 1.139275
H -2.093211 -1.724251 -0.251217
H 3.824529 -0.121606 0.012899
H 0.485530 1.276763 -2.038436

22
oh2.7h2o, E= -610.422257
O 0.261275 -1.763433 -0.376987
O 2.773583 -0.719494 0.360665
O 2.083551 1.932832 -0.655509
O 0.466841 -0.854624 2.083245
O 0.128261 0.332968 -1.806445
O -0.502018 1.325507 0.783646
O -3.169522 1.016035 0.910245
O -2.338040 -1.072942 -1.148334
H -1.496760 -1.425069 -0.770600
H -2.728516 -0.527597 -0.449490
H -0.788884 0.238797 -2.100560
H 0.291399 -0.569455 -1.345010
H 0.270637 -1.378772 1.252062
H 1.423292 -0.721822 1.975506
H 1.980524 -1.165392 -0.015609
H 2.725473 0.187203 0.009820
H -0.339011 0.997293 -0.126592
H -0.153943 0.598610 1.365139
H -2.178612 1.209304 0.923826
H 1.480818 1.501488 -1.292582
H 1.486251 2.135200 0.078469
H 0.395877 -2.654575 -0.708585

22
oh2.7h2o, E= -610.422052
O -0.000627 -0.907215 -1.422297
O 0.000467 1.282693 -1.609259
O 2.486582 -1.240504 -0.279512
O -2.487855 -1.238823 -0.279329
O 2.069268 1.522655 0.192748
O -2.067660 1.524005 0.193174
O -0.000769 -1.741148 1.347124
O 0.000695 0.923349 2.015896
H -0.000119 -1.102586 -2.366781
H 1.673188 -1.100010 -0.822657
H 2.116710 -1.605253 0.537280
H -0.000785 1.187306 -2.569092
H -1.674549 -1.098998 -0.822770
H -2.117981 -1.603731 0.537391
H -0.000752 -1.612686 0.385561
H -0.000382 -0.814902 1.694527
H -2.454584 0.631271 0.121384
H -1.407179 1.522021 -0.534395
H -0.773654 1.223635 1.492658
H 0.775126 1.222987 1.492415
H 2.455285 0.629520 0.120982
H 1.408866 1.521332 -0.534852

22
oh2.7h2o, E= -610.421962

O 1.093282 -1.053764 -1.000560
O -0.521286 1.120904 -1.084933
O -0.909898 -3.034550 -0.194418
O -2.100097 -0.551025 0.065073
O 0.250786 -0.952994 1.695616
O -2.538396 2.437801 0.021337
O 1.234837 1.806531 0.894419
O 3.475114 0.452059 -0.288562
H 0.804684 -0.985413 0.890590
H -0.627849 -0.734985 1.332754
H -1.561426 -2.307032 -0.183570
H -1.526932 0.038269 -0.527116
H 0.529651 -0.241991 -1.119817
H -0.470764 1.429352 -1.993680
H 0.979052 1.087636 1.490613
H 0.635190 1.681450 0.119007
H 0.492265 -1.813651 -1.067830
H -0.431726 -2.885057 0.633423
H -1.676844 2.058141 -0.365300
H -2.755906 0.065802 0.411215
H 2.894269 -0.238910 -0.640063
H 2.841608 1.046711 0.156003

25
oh2.8h2o, E= -686.888466
O -0.850830 1.457356 -0.535049
O 1.411057 3.079365 -0.110443
O 2.396782 0.625870 -0.420591
O 0.289331 -0.674621 -1.586636
O -1.945606 -1.712755 -0.412499
O -3.425437 0.769012 0.093066
O -0.978963 -0.200591 1.726318
O 1.744124 -0.967078 1.788009
O 1.507886 -2.681995 -0.496521
H -0.411694 0.696562 -1.024542
H -0.185727 2.169682 -0.468308
H 1.423156 3.219015 0.841557
H 1.883312 2.206891 -0.239345
H 1.706334 0.175973 -0.969363
H 2.322335 0.155671 0.438001
H -1.171152 -1.393662 -0.942604
H -2.620611 -1.020842 -0.508261
H -3.151095 0.521344 0.985842
H -2.616582 1.201491 -0.254356
H -0.847679 0.509701 1.063739
H -1.300246 -0.934739 1.158820
H 1.691223 -1.717100 1.164612
H 0.809374 -0.702452 1.917192
H 1.004399 -1.921975 -0.981301
H 0.277898 -0.722076 -2.546910

25
oh2.8h2o, E= -686.887341
O 2.189589 1.278100 -0.858769
O 3.496863 -1.035162 0.726890
O 0.931600 -1.574474 0.416967
O 0.207984 -0.397444 -1.677703
O -1.682773 1.453425 -1.234045
O -2.765583 -0.210877 0.892944
O -0.432046 0.303563 2.000519
O 0.141385 2.548491 0.562635
O -1.664843 -2.310249 -0.712139
H 1.483959 0.696920 -1.249846
H 2.745565 0.675386 -0.343459
H 2.549237 -1.372069 0.680899
H 0.700187 -1.105718 -0.489609
H 0.289526 -2.297891 0.440157
H -1.009044 0.780803 -1.524704
H -2.247481 0.962532 -0.615707
H -1.908607 -0.012846 1.380882
H -3.477758 -0.054495 1.518276
H -0.181218 1.173593 1.596651
H 0.134554 -0.352883 1.542557
H -1.124831 -1.657488 -1.207811
H -2.217090 -1.746768 -0.144405
H 0.971178 2.258788 0.127081
H -0.549336 2.336773 -0.106162
H 0.463744 -0.727622 -2.543181

25
oh2.8h2o, E= -686.886503
O 0.288145 -1.624151 0.238998
O 2.218250 -0.865623 -1.093170
O 0.779885 0.680709 1.473870
O 3.685440 0.080679 0.916845
O -1.883971 -1.415021 -1.430812
O 1.046911 1.693333 -1.155772
O -1.724093 1.397772 -1.502387
O -1.959852 1.550325 1.267819
O -2.365947 -1.221121 1.387589
H -1.434807 -1.392883 1.155392
H -2.390008 -0.242826 1.456421
H -2.481733 -1.419941 -0.663415
H -1.022479 -1.575756 -0.977580
H 0.913708 1.556673 -0.196906
H 1.486635 0.860814 -1.408500
H 1.367645 -1.268942 -0.553238
H 2.442951 -1.463046 -1.810588
H -1.809951 0.426029 -1.606830
H -0.761210 1.566603 -1.553121
H -1.953499 1.592589 0.283353
H -1.023143 1.469106 1.509842
H 3.229522 -0.289465 0.097728
H 0.559742 -0.225488 1.111093
H 1.731843 0.648566 1.646987
H 0.466648 -2.457259 0.685522

25
oh2.8h2o, E= -686.885921
O -1.245513 -1.680884 -0.081399
O -2.340051 -0.000501 1.938301
O 0.407353 -0.734818 1.641777
O 0.714724 -0.921938 -1.719835
O -2.289736 -0.028425 -1.852708
O -1.988687 2.058073 -0.012274
O 2.806556 -1.291599 0.252180
O 3.458261 1.612467 -0.010165
O 0.797077 1.302241 -0.134385
H -1.491735 -2.609079 -0.081441
H -2.025334 -0.719646 -1.174753
H -1.513578 0.012613 -2.423262
H -0.191996 -1.229789 0.977328
H -0.257160 -0.395441 2.263044
H -0.014757 -1.334177 -1.172479

H 1.531626 -1.210417 -1.272112
H -2.176530 -0.683505 1.254883
H -2.320565 0.825037 1.414575
H -2.231036 1.421698 -0.719235
H -1.016004 2.035995 -0.033289
H 0.653448 0.684377 0.626080
H 0.713315 0.668375 -0.889433
H 2.105934 -1.185556 0.922078
H 3.225368 -0.417245 0.204925
H 2.449125 1.619830 -0.068845

25
oh2.8h2o, E= -686.885738
O -1.340511 -1.586096 0.224592
O -2.285953 0.593017 1.877472
O 0.399350 -0.254339 1.617473
O 0.780129 -1.432978 -1.498092
O -2.979766 -0.536448 -1.433126
O -1.950390 1.986634 -0.579555
O 2.803770 -1.279598 0.564694
O 3.493088 1.368617 -0.336958
O 0.800901 1.171192 -0.700892
H -1.540101 -2.494454 0.468130
H -2.324217 -1.022733 -0.787233
H -0.214700 -0.881307 1.107463
H -0.249701 0.243887 2.141770
H -0.021871 -1.611117 -0.943607
H 1.534192 -1.596999 -0.900285
H -2.198115 -0.255789 1.401296
H -2.234626 1.243245 1.147544
H -2.364496 1.217067 -1.018450
H -0.994816 1.857229 -0.726987
H 0.661980 0.801045 0.203639
H 0.765243 0.340684 -1.238389
H 2.094886 -0.968095 1.155918
H 3.217301 -0.456977 0.244342
H 2.535769 1.418108 -0.583573
H 3.568334 1.926200 0.443546

25
oh2.8h2o, E= -686.885612
O -0.219660 -1.488958 0.961450
O 1.989576 -1.625849 -0.352379
O 0.372533 0.812652 1.832159
O 3.086673 0.621352 1.086683
O -1.584610 -1.469816 -1.434032
O -0.803528 1.207776 -1.886705
O -1.928583 2.004224 0.498692
O -2.973532 -0.571978 0.898392
O 1.980619 0.889120 -1.578824
H -2.079383 -0.920124 1.078295
H -2.805916 0.386922 0.778841
H -2.407124 -1.220186 -0.978106
H -0.999529 -1.616996 -0.656606
H 2.399469 1.238414 -0.774892
H 1.952672 -0.065808 -1.372502
H 1.105715 -1.625041 0.165847
H 2.021927 -2.451289 -0.842760
H -1.053164 0.260165 -1.862705
H 0.172612 1.213359 -1.817926
H -1.550736 1.827224 -0.395614
H -1.192021 1.809252 1.101419
H 2.992771 -0.266798 0.704820
H 0.105335 -0.162246 1.513161
H -0.252730 -2.195922 1.612972
H 2.234198 0.760902 1.542265

25
oh2.8h2o, E= -686.885214
O -0.602613 0.506775 -1.643496
O -2.343901 -1.614534 -0.926666
O 0.489469 -1.710443 -0.880733
O 0.810451 1.805612 0.152580
O -2.175251 1.988656 -0.067381
O -2.604099 -0.246406 1.564841
O 3.653044 1.043061 -0.350443
O 2.769644 -1.327151 0.583629
O 0.275563 -0.424165 1.673225
H -0.540294 0.772299 -2.564546
H -1.710738 1.445770 -0.764891
H -1.436425 2.454581 0.343658
H 0.179872 -0.827986 -1.280312
H -0.346437 -2.206107 -0.861046
H 0.361484 1.363994 -0.634273
H 1.762850 1.777645 -0.027161
H -1.914269 -0.828307 -1.325174
H -2.575760 -1.299996 -0.030105
H -2.596577 0.598227 1.064832
H -1.674285 -0.347760 1.837417
H 0.181432 -0.970372 0.867765
H 0.452757 0.467165 1.288961
H 3.382624 0.125162 -0.029060
H 2.202323 -1.050694 1.324890
H 2.092982 -1.644864 -0.055408

25
oh2.8h2o, E= -686.884855
O 0.358671 -1.344813 -0.835677
O 2.090529 0.354028 -1.607256
O 0.906568 -0.475235 1.484475
O 3.666610 -0.553456 0.535334
O -1.920095 -0.242925 -1.937924
O -1.866594 2.027779 -0.281096
O -1.873437 0.338947 1.904077
O -2.191989 -1.993158 0.361616
O 0.906668 2.142003 0.244415
H -1.293210 -1.895512 -0.006892
H -2.251478 -1.242211 0.987985
H -2.477199 -0.786322 -1.357055
H -1.030466 -0.606039 -1.723826
H 0.946120 1.451443 0.924045
H 1.348736 1.725029 -0.519673
H 1.354697 -0.359866 -1.404431
H -1.913201 1.328258 -0.967630
H -0.911885 2.214504 -0.177109
H -2.018982 1.008456 1.195285
H -0.912163 0.198493 1.882418
H 3.510245 -0.312100 -0.390312
H 0.668779 -0.916936 0.578195
H 0.678569 -2.221539 -1.070795
H 2.795412 -0.443523 0.954008
H 0.890578 -1.167497 2.152070

25

oh2.8h2o, E= -686.884796
O -0.006870 -0.639665 -1.247647
O -2.257690 -2.219641 0.326035
O 2.242573 -0.304527 1.213235
O 1.852684 1.174586 -0.798809
O -1.581954 1.806139 0.941208
O 0.534611 3.267436 -0.283288
O -2.672133 0.232615 -1.137785
O -0.468276 -0.580553 1.618844
O 2.138722 -2.522882 -0.648384
H 0.699010 0.074617 -1.244323
H 2.577208 1.227376 -1.428055
H 2.835292 0.115958 1.841335
H 2.160707 0.337395 0.398123
H 0.510256 -0.513863 1.658501
H -0.820226 0.337932 1.511718
H 1.409583 -2.044034 -1.077169
H 2.359536 -1.934672 0.092095
H -2.077026 1.412275 0.192569
H -0.898963 2.381047 0.537649
H -1.771136 0.055412 -1.474747
H -2.841369 -0.574007 -0.617197
H 1.126357 2.435821 -0.530955
H -1.671586 -2.543514 -0.367865
H -1.646109 -1.753328 0.937757
H -0.204879 -0.722464 -0.296704

25
oh2.8h2o, E= -686.883891
O -2.641126 0.912141 0.023989
O -1.953779 -1.576725 -0.219351
O -0.136011 -1.376271 2.011751
O -0.582879 1.216818 1.895609
O 1.748537 -1.688742 -0.129687
O 3.416710 0.309968 0.028564
O 1.208590 2.193380 0.118632
O -0.467553 1.393605 -1.874683
O -0.069903 -1.200316 -1.884208
H -2.438353 -0.079498 -0.079690
H -3.597451 0.998636 0.038960
H 1.191557 -1.686332 0.668345
H 1.082279 -1.530694 -0.891742
H -1.387166 1.259617 1.341893
H 0.125762 1.624105 1.329546
H 2.783682 -0.496871 -0.059541
H -0.026491 -1.623527 2.933435
H -0.274284 -0.379600 2.000912
H 0.672202 1.952446 -0.680331
H -1.306186 1.410606 -1.387922
H -0.275837 0.408060 -1.978295
H -0.055611 -1.691358 -2.709213
H 2.004583 1.632841 0.076742
H -1.229457 -1.506499 -0.957752
H -1.449917 -1.762797 0.589727

25
oh2.8h2o, E= -686.883626
O -2.665516 0.946157 0.084449
O -0.845202 1.253151 -1.810777
O -0.294121 -1.503060 -1.873667
O -1.752268 -1.766875 0.251362
O 3.383155 0.591635 -0.264356
O 1.926947 -1.561852 -0.039903
O 0.181311 -1.279818 1.828680
O -0.433914 1.269291 1.948696
O 1.018712 2.263395 -0.136013
H -2.016194 1.100157 -0.665965
H -3.426743 1.502807 -0.097893
H 1.864133 1.779976 -0.171727
H -2.281090 -0.952989 0.294163
H -1.013759 -1.641732 0.967483
H 1.315726 -1.620916 -0.793799
H 2.835509 -0.272386 -0.156274
H -0.540999 -2.155819 -2.533895
H -0.902425 -1.658952 -1.070805
H -0.158184 0.299592 1.995291
H -1.298247 1.244955 1.509061
H 0.568456 1.952259 0.690894
H 0.273795 -1.780725 2.642601
H 1.299571 -1.482961 0.766735
H -0.098179 1.659168 -1.295707
H -0.574199 0.331378 -1.987939

25
oh2.8h2o, E= -686.883558
O 0.291468 0.076125 1.580604
O 2.060565 -1.458278 0.481077
O 0.631021 1.535814 -0.582124
O 3.484845 0.976436 -0.235317
O -1.475516 -1.761884 1.817482
O -1.948278 -1.689159 -1.027692
O -2.274738 1.034695 -1.346542
O -1.662269 2.231029 1.154824
O 0.645559 -1.041155 -1.883325
H -1.123955 1.474262 1.448816
H -2.121857 1.877646 0.369126
H -0.788416 -0.987853 1.794942
H 0.637989 -0.077878 -1.762423
H 1.229330 -1.342891 -1.158720
H 1.377284 -0.891746 0.982929
H -1.841077 -1.809788 -0.065075
H -1.036532 -1.588629 -1.375948
H -2.358780 0.058984 -1.243896
H -1.319324 1.154458 -1.445412
H 3.254658 0.070195 0.021454
H 0.528062 0.973390 0.248406
H 0.674566 0.466628 2.372311
H 2.623085 1.366433 -0.453978
H 0.137759 2.331635 -0.339568
H 2.105960 -2.303810 0.935131

25
oh2.8h2o, E= -686.882925
O 0.170118 1.540101 -1.350468
O 2.695391 1.615281 -0.102550
O 2.081691 -0.225422 1.669516
O -0.474807 -0.834759 1.400362
O 0.236581 -1.262926 -1.043535
O -2.557866 -1.746456 -0.324765
O -3.974654 0.830396 -0.290288
O -1.650470 1.461305 0.767665
O 2.997927 -1.293274 -0.872003
H 0.155554 0.561408 -1.394347
H 1.052308 1.734327 -0.983379

H 3.135066 2.379802 0.278446
H 2.432931 1.024222 0.666474
H 1.088686 -0.408086 1.649374
H 2.461862 -0.901569 1.082614
H -1.935604 -1.431870 0.365322
H -3.167316 -0.998974 -0.440768
H -3.096006 1.128132 0.141377
H -1.035968 1.664644 0.029183
H -1.262639 0.627927 1.157680
H 2.053027 -1.422076 -1.095356
H 3.129220 -0.337362 -0.946755
H 0.030255 -1.188134 -0.052316
H -0.588265 -1.634306 -1.388092
H -0.644396 -1.472043 2.099073

25
oh2.8h2o, E= -686.882591
O 0.968035 1.014572 1.254478
O 2.353121 -1.388725 1.484722
O -0.509075 -0.617311 1.544185
O -0.607855 1.961466 -0.719220
O 2.365697 1.487059 -1.063482
O 2.162392 -1.352030 -1.377752
O -2.766081 0.964196 0.834758
O -3.267891 -1.305267 -0.855198
O -0.609997 -0.793660 -1.108426
H 0.692290 1.351561 2.113290
H 2.069268 1.388921 -0.134553
H 1.576662 1.842375 -1.492872
H 0.287592 -1.117191 1.795629
H -0.048126 1.697373 0.058213
H -1.508762 1.872656 -0.354395
H 2.081652 -0.448245 1.489834
H 2.379130 -1.585924 0.527958
H 2.401182 -0.405498 -1.391407
H 1.186636 -1.323362 -1.413145
H -0.516864 -0.798554 -0.109453
H -0.582476 0.170173 -1.285953
H -2.036958 0.507348 1.292070
H -3.131822 0.271005 0.254434
H -2.318135 -1.180325 -1.107816
H -3.238032 -2.004716 -0.194355

25
oh2.8h2o, E= -686.880694
O 2.348400 1.436248 0.077937
O 2.368091 -1.316383 -0.273506
O 0.334359 -0.982593 -1.925913
O 0.307173 1.642374 -1.777082
O -1.317594 -1.921447 -0.298267
O -3.272530 -0.349095 -0.073122
O -1.767857 1.850559 0.328000
O 0.435087 1.145440 1.961283
O 0.424805 -1.497421 1.921964
H 2.529571 0.475086 -0.045465
H -0.648751 -1.564126 -1.040805
H -0.788252 -1.927067 0.519284
H 1.720089 1.636286 -0.656084
H -0.434826 1.796968 -1.173678
H -2.468146 -1.043166 -0.186380
H 0.380006 -1.403619 -2.788194
H 0.299879 0.644674 -1.929144
H -0.352858 1.451374 1.474980
H 1.195630 1.335809 1.345920
H 0.407856 -0.495166 1.975621
H 0.459641 -1.815657 2.827564
H -2.353238 2.605323 0.432642
H -2.351876 1.059116 0.176918
H 1.869826 -1.568823 0.518837
H 1.655979 -1.248472 -0.982365

28
oh2.9h2o, E= -763.349130
O 0.488870 2.104568 1.603710
O -1.927885 0.917497 1.506708
O -1.659024 -1.690339 1.418405
O 2.458811 0.148150 1.382579
O 1.122319 -2.222083 1.002113
O 0.994848 -1.663369 -1.622231
O 2.198491 0.476239 -1.338426
O -1.573852 -1.450749 -1.420005
O -2.097306 1.087767 -1.294309
O 0.343346 2.531673 -1.164426
H 1.201297 1.426177 1.630509
H 2.731521 0.288394 0.457285
H 2.005601 -0.729914 1.360000
H -0.503527 2.068225 -1.303019
H 0.490621 2.413735 0.671827
H -1.046135 1.355822 1.655437
H -2.139135 1.119366 0.575376
H 1.108050 -2.118927 0.011020
H 0.193061 -2.106697 1.265017
H -1.890600 0.099040 -1.401766
H -2.792840 1.294064 -1.923926
H -2.228430 -2.083502 2.084602
H -1.749780 -0.692043 1.515808
H 1.690159 -0.482561 -1.520862
H 1.392193 -2.324467 -2.194998
H 1.031680 1.868901 -1.388107
H -1.704526 -1.740250 -0.499820
H -0.578143 -1.570188 -1.587319

28
oh2.9h2o, E= -763.348650
O 2.778206 -1.163387 0.578934
O 0.314136 -2.128167 -0.603402
O 0.660084 -0.142763 -2.150990
O 0.396929 -1.519421 2.087174
O -1.013543 0.828391 2.126404
O 0.369606 2.559537 0.777711
O -1.161107 1.873036 -1.499970
O -2.886381 0.432997 0.154917
O -2.259168 -2.227218 -0.711039
O 2.492188 1.358209 -0.638969
H -2.393111 0.963556 -0.508759
H -1.773856 0.650620 1.521451
H -0.524230 -0.027538 2.193938
H -2.773463 -0.493297 -0.133683
H -1.223532 -2.205394 -0.663513
H -0.570350 1.160074 -1.818090
H -0.651937 2.292135 -0.781679
H 1.336855 -1.282604 1.997414
H 0.214101 -1.851383 1.176891
H 1.938550 0.902377 -1.302177

H 1.847114 1.840775 -0.088128
H 2.030045 -1.527506 0.066571
H 2.844239 -0.242637 0.253595
H 0.528439 -0.984185 -1.560811
H 0.389048 3.380694 1.275856
H -0.130949 1.885829 1.349145
H 0.648152 -2.985603 -0.884894
H 0.737289 -0.445613 -3.059285

28
oh2.9h2o, E= -763.347814
O -0.168353 2.204490 1.593308
O -2.060150 0.300422 1.584535
O -1.032966 -2.096451 1.518334
O 2.267518 0.937846 1.281816
O 1.799451 -1.797321 1.147954
O 1.302232 -1.477049 -1.453493
O 1.868639 0.959883 -1.503533
O -1.216047 -1.903399 -1.316110
O -2.418008 0.392193 -1.198783
O -0.447248 2.442748 -1.188368
H 0.717099 1.771089 1.618408
H 2.273681 1.051049 0.311042
H 2.147138 -0.032377 1.387646
H -1.154704 1.780496 -1.291845
H -0.272525 2.458919 0.650543
H -1.364314 1.005268 1.704200
H -2.369397 0.422719 0.666631
H 1.650603 -1.752369 0.158737
H 0.936833 -2.035917 1.520382
H -1.946509 -0.501201 -1.299817
H -3.184772 0.365855 -1.776799
H -1.437798 -1.163279 1.629507
H 1.654729 -0.073634 -1.545886
H 1.794950 -2.069508 -2.027519
H 0.381008 1.956318 -1.391619
H 2.525705 1.143370 -2.179395
H -1.307834 -2.273963 -0.422903
H -0.204446 -1.759720 -1.436599

28
oh2.9h2o, E= -763.346505
O 1.988631 1.979716 0.684172
O -0.532311 1.619303 1.699929
O -1.857887 1.716694 -0.523257
O 3.192729 -0.525592 0.292074
O 1.162565 -1.195840 -1.394820
O -1.450076 -1.048462 -2.019011
O -3.051877 -0.805046 0.219606
O -1.112872 -1.005767 2.222345
O 0.760822 1.473744 -1.766902
H 2.522503 1.161873 0.729773
H 1.648984 1.961416 -0.237847
H -0.153502 1.581996 -1.456505
H 0.939834 0.500320 -1.706753
H 0.421846 1.735152 1.471477
H -0.651691 0.687815 2.007392
H -1.349752 1.714406 0.359418
H -2.238072 2.596204 -0.605468
H -1.553080 -0.089352 -2.054452
H -0.486800 -1.172027 -1.792406
H 2.525764 -0.704492 -0.422428

H 1.577829 -1.675380 -2.118196
H 3.038864 -1.245546 0.915078
H -0.420598 -1.553647 1.804667
H -1.867876 -1.037748 1.595190
H -2.553267 -1.167797 -0.544257
H -2.928482 0.148203 0.075810
O 0.968523 -2.389217 0.848288
H 0.981514 -1.997665 -0.119894

28
oh2.9h2o, E= -763.346319
O -0.128508 -0.001338 -1.613396
O 1.611785 1.577820 -0.497782
O -1.532087 1.219168 0.186012
O 3.507536 -0.036238 0.609074
O -3.785508 -0.287211 0.461115
O -0.476677 3.670792 -0.162911
O 0.493607 0.267755 1.815383
O -1.799918 -2.024747 -0.779600
O 1.898837 -1.808438 -1.053667
O 0.232353 -2.352272 1.083416
H -1.076299 0.754724 -0.602564
H -0.984940 0.906973 0.943811
H 0.995313 0.664753 1.079822
H 0.408094 -0.697562 1.602305
H -0.598821 -2.366003 0.560341
H 0.915825 -2.361376 0.376279
H -3.054276 0.370744 0.366427
H -3.673073 -0.639673 1.349747
H 0.981452 0.997801 -1.040404
H 1.172817 2.437308 -0.410111
H -1.210312 -1.336974 -1.177042
H -2.588876 -1.539300 -0.485418
H 1.206903 -1.174694 -1.362221
H 2.582050 -1.264908 -0.624415
H 3.008560 0.700383 0.197380
H 3.007897 -0.220064 1.414148
H -0.981343 2.807456 -0.020487
H -0.282340 0.158069 -2.548752

28
oh2.9h2o, E= -763.346230
O 0.115707 -0.045781 -1.483025
O 1.819513 1.101959 0.091959
O -1.400894 1.444107 0.057456
O 4.035635 -0.326034 0.065580
O -3.832065 0.224749 0.002242
O 0.344900 3.525987 -0.130065
O -0.072288 0.176805 2.056590
O -1.956844 -1.776153 -0.996290
O 1.691996 -2.131993 -0.664042
O -0.370637 -2.415319 1.205101
H -0.909625 0.906436 -0.641521
H -1.052756 1.037252 0.892877
H 0.749139 0.418944 1.591422
H -0.199337 -0.782903 1.849763
H -1.057113 -2.314031 0.509873
H 0.452773 -2.481226 0.677200
H -3.022949 0.793780 0.004336
H -3.868061 -0.131807 0.895791
H 1.192759 0.668926 -0.585473
H 1.572094 2.044385 0.099335

H -1.219136 -1.176588 -1.271307
H -2.698905 -1.182580 -0.791038
H 1.134948 -1.414344 -1.058112
H 2.527081 -1.703699 -0.424081
H 3.266860 0.324724 0.109144
H -0.398299 2.880123 -0.014409
H 0.323273 3.747087 -1.066199
H 0.207069 0.138894 -2.421646

28
oh2.9h2o, E= -763.346181
O -0.242811 -0.042611 -1.543010
O 1.388483 1.712138 -0.472569
O -1.682782 0.937684 0.366297
O 3.488415 0.276996 0.494462
O -3.878120 -0.587818 0.448880
O -0.826773 3.486911 -0.113837
O 0.531302 0.221886 1.857981
O -1.617425 -2.254082 -0.679160
O 2.017473 -1.605681 -1.164324
O 0.564317 -2.394522 1.056008
H -1.196283 0.563276 -0.451732
H -1.074267 0.686306 1.100155
H 0.945574 0.697174 1.115035
H 0.557348 -0.740236 1.619666
H -0.297248 -2.497269 0.599768
H 1.188371 -2.306158 0.301561
H -3.097843 0.053267 0.473043
H 0.805621 1.075782 -0.993298
H 0.834997 2.497009 -0.314444
H -1.128368 -1.491556 -1.080150
H -2.448922 -1.877526 -0.355426
H 1.237901 -1.049191 -1.404098
H 2.651634 -1.001909 -0.739308
H 2.889490 0.967344 0.137996
H 3.058338 0.013563 1.317407
H -1.257499 2.633332 0.139902
H -1.145039 3.658779 -1.005421
H -0.460442 0.110803 -2.466482

28
oh2.9h2o, E= -763.346099
O -1.300333 -1.608786 1.818990
O -1.714875 0.974071 1.715288
O 0.583740 2.329656 1.258691
O 1.429557 -1.971559 1.005559
O 2.577401 0.361588 1.015754
O 0.000478 2.332585 -1.434213
O -2.394527 0.848446 -0.980184
O -1.821316 -1.671010 -0.966055
O 0.637532 -1.934766 -1.428788
O 1.882279 0.382067 -1.806385
H -1.456086 -0.600079 1.860487
H -2.106002 1.068289 0.823781
H -0.865107 1.486659 1.663304
H -0.811462 -1.815936 -1.213627
H -1.912281 -1.978557 -0.051555
H 0.594328 -1.964460 1.501090
H 1.148041 -2.047734 0.018386
H 0.412304 2.473124 0.296426
H 1.326028 1.695290 1.274127
H 1.410620 -0.501920 -1.762261

H 0.891750 -2.660351 -2.004818
H 3.424305 0.280607 1.461527
H 2.136758 -0.548937 1.068177
H -0.844500 1.849785 -1.393496
H -2.170675 -0.143697 -1.020858
H -3.204259 0.968201 -1.482765
H 2.315634 0.451214 -0.940344
H 0.671112 1.650163 -1.686820

28
oh2.9h2o, E= -763.345108
O 0.395793 0.272828 -1.529136
O -0.348582 0.153495 2.306601
O -0.243027 -2.369983 1.097950
O -2.662913 -2.360319 -0.442047
O -2.221250 0.047372 -1.153396
O -2.036270 1.710222 0.698320
O -0.062485 3.153285 -0.635392
O 1.629761 1.253487 0.626970
O 1.681587 -2.058931 -0.826372
O 3.833846 -0.233691 -0.035581
H 0.467858 0.524550 1.922278
H -1.044409 0.670331 1.851765
H 0.119433 2.454953 -1.280758
H -0.813109 2.785689 -0.120907
H 1.034166 -2.249327 -0.102679
H 2.455022 -1.658115 -0.395696
H 1.129133 0.841004 -0.133597
H 1.378071 2.186373 0.528131
H -0.311130 -1.518878 1.584436
H -1.091983 -2.458203 0.622307
H -0.591186 0.131937 -1.428907
H -2.701456 0.333193 -1.935908
H -2.525515 -1.376133 -0.756330
H 3.876737 -0.003061 -0.969513
H 3.166157 0.390472 0.326305
H -2.189427 1.022330 -0.086627
H -2.902500 1.999232 0.996659
H 0.812452 -0.618462 -1.484291

28
oh2.9h2o, E= -763.343571
O -0.533433 1.763153 -1.784977
O 1.899824 1.447888 -1.136046
O 2.488318 -1.200591 -0.864524
O -1.835091 -0.607081 -1.833637
O -0.083770 -2.567400 -0.981001
O -0.782646 -1.835294 1.473131
O -2.507728 -0.177778 0.834868
O 1.563987 -0.739951 1.858316
O 1.284010 1.921105 1.512315
O -1.375692 2.338055 0.732411
H -0.987628 0.889071 -1.924335
H -2.247713 -0.478481 -0.953594
H -1.202541 -1.346831 -1.675879
H -0.484554 2.235177 1.129512
H -0.925304 2.104288 -0.938748
H 0.916301 1.583468 -1.473052
H 1.784582 2.082736 0.698023
H -0.352875 -2.395768 -0.030157
H 0.822879 -2.229704 -1.038619
H 1.395629 0.948396 1.696853

H 3.373881 -1.356819 -1.203238
H 2.283184 -0.251953 -1.050485
H -1.798319 -0.910379 1.176537
H -1.017878 -2.524869 2.099278
H -1.823823 1.475200 0.877679
H -3.343575 -0.355905 1.272785
H 2.018189 -1.044694 1.058185
H 0.647329 -1.159775 1.792394

28
oh2.9h2o, E= -763.343176
O -2.362155 0.333486 -1.744752
O -2.572683 -2.109793 -0.471260
O 0.302529 0.094290 -1.036473
O -0.354295 1.415351 1.189566
O -2.628958 2.227886 0.381394
O -1.272332 -1.195407 1.853922
O 0.549306 -2.353364 0.127804
O 3.205402 -1.773909 0.398247
O 2.997176 0.679215 -1.017576
H -1.384657 0.320033 -1.732614
H -2.588724 1.025325 -1.090914
H -1.705445 1.948263 0.780388
H 0.133388 0.535029 -0.161228
H -0.151663 1.478317 2.128444
H 2.761615 1.439915 -0.446090
H 3.171972 -0.079906 -0.426648
H 2.252459 -2.044348 0.410922
H 3.617760 -2.381272 -0.223482
H 0.018034 -2.110651 0.919777
H 0.439428 -1.549298 -0.433274
H -1.020204 -0.280943 1.599587
H -1.957663 -1.456767 1.207742
H -1.695535 -2.477769 -0.643435
H -2.598395 -1.266558 -0.982895
H 1.233713 0.320509 -1.255344
O 1.770044 2.796199 0.328712
H 1.449105 3.350193 -0.389457
H 0.952550 2.318302 0.661849

28
oh2.9h2o, E= -763.342945
O -1.439022 -1.488343 -1.002728
O 1.088728 -2.032959 -0.955851
O -1.193522 -1.999330 1.718271
O 2.081071 0.195920 -1.795836
O -0.714391 0.779016 -2.237626
O -2.901753 0.073504 0.648799
O -1.539605 2.304941 -0.140019
O 0.435566 2.934748 1.415177
O 2.412139 1.037313 0.824098
O 1.624260 -1.323345 1.752768
H -1.028796 -0.090535 -1.854272
H -1.953151 -2.106357 -1.529749
H -0.358759 2.705016 0.797472
H 0.077012 -1.887374 -1.008691
H 1.295243 -2.041583 -0.005280
H 2.374161 0.877181 -0.141817
H 1.762970 1.749203 1.010626
H 2.752772 0.044542 -2.465794
H 1.734083 -0.719352 -1.512344
H -0.273762 -1.728419 1.867674

H -1.292189 -1.975848 0.737266
H 2.258132 -1.612837 2.413815
H 1.907900 -0.408030 1.467994
H -1.214415 1.863797 -0.965316
H 0.253801 0.711337 -2.229293
H -2.111097 1.606647 0.262811
H -2.492628 -0.465014 -0.081776
H -2.519052 -0.374107 1.420247

28
oh2.9h2o, E= -763.342856
O 0.149100 0.534927 1.950097
O -0.352430 2.506549 0.211474
O -2.185398 -0.473199 0.996259
O -2.808603 1.528428 -0.813375
O -1.623178 -2.486720 -1.115713
O 0.952980 -1.959546 -0.780969
O 0.668947 -1.999965 1.910695
O 2.744669 -0.015046 -1.561773
H 1.992401 0.394728 -2.020723
H 2.758262 0.469222 -0.702753
H 0.522157 -0.998015 2.005831
H -0.205751 -2.376276 2.054367
H 1.729527 -1.436013 -1.082258
H 0.980067 -2.004953 0.206782
H 0.059609 0.856112 2.852300
H 1.950534 2.230243 0.408487
H 1.647127 1.019874 1.285623
O 2.382639 1.440848 0.758251
H -1.300674 2.490012 -0.016311
H -0.249760 1.824555 0.931662
H -1.331207 -0.118942 1.363078
H -1.965835 -1.205971 0.398580
H -0.632363 -2.331734 -1.158206
H -2.211209 1.215614 -1.508508
H -2.742482 0.818541 -0.133774
O -0.054535 0.669637 -1.850366
H 0.038019 -0.171433 -1.375265
H -0.031948 1.357125 -1.145554

28
oh2.9h2o, E= -763.340703
O 0.865542 2.771761 -0.377494
O -1.299189 1.330095 -1.102798
O -2.625042 -0.129347 0.577832
O 0.031053 -0.999077 -1.264974
O -1.995289 2.906051 0.898005
O -0.644218 -1.844061 1.457536
O 3.034842 1.173485 0.366265
O 2.878728 -1.161293 -1.194009
O 2.181453 -1.289190 1.584250
O -2.260955 -2.650019 -0.794223
H -3.062871 0.547493 1.105355
H 0.389171 3.232323 0.323097
H 0.160806 2.207506 -0.786228
H -1.304248 -1.123427 1.370976
H -1.048670 -2.546169 0.921257
H -2.740962 -1.911139 -0.387554
H -1.513897 -2.202348 -1.235697
H -2.156688 0.396222 -0.153607
H -1.707779 1.535115 -1.948413
H -0.389735 -0.096583 -1.305934

H 0.098105 -1.174652 -0.311815
H 2.291864 1.766334 0.128570
H 2.681145 0.550035 1.025421
H 1.945920 -1.101893 -1.469323
H 3.082511 -0.262096 -0.862859
H 1.239096 -1.522383 1.640659
H 2.457284 -1.527207 0.677845
H -1.756456 2.365633 0.065128

28
oh2.9h2o, E= -763.340523
O -3.613684 -1.453302 0.165392
O -3.154282 1.154381 -0.238979
O -0.902824 1.142274 1.108920
O -0.811286 -1.394146 0.250228
O -0.307979 1.858901 -1.067760
O 1.578495 0.589924 2.210396
O 1.773027 -1.876797 1.096929
O 2.811864 -0.909100 -1.121975
O 2.379120 1.770880 -0.204420
O 0.192021 -0.709856 -2.221602
H -3.949068 -1.418127 1.066614
H -3.530699 -0.495977 -0.098678
H -2.731837 1.432508 -1.061717
H -2.365565 1.198973 0.388093
H -0.775395 -0.443182 0.536752
H -0.225949 -1.132283 -1.441411
H 1.145112 -0.795638 -2.046384
H 2.469811 -1.337720 -0.278041
H 3.594694 -1.399819 -1.384824
H 1.778106 -1.048484 1.643848
H 0.827271 -1.970851 0.862491
H 1.460551 1.912619 -0.527632
H 2.657847 0.940148 -0.626948
H 0.623976 0.723994 2.001884
H 2.006657 1.090504 1.482509
H -0.563370 2.015823 0.880203
H -0.214805 0.961358 -1.462203
H -1.763109 -1.619120 0.248413

31
oh2.10h2o, E= -839.809614
O -1.798360 -2.326206 -0.745013
O 0.594025 -1.243495 -0.766971
O 0.055406 0.978854 -1.827368
O -2.509119 0.154134 -1.712364
O -1.919032 -1.324277 1.996262
O 2.107785 2.221362 -0.514598
O -3.121563 1.000406 0.882859
O -0.630434 2.141093 0.544229
O 0.557124 -0.032177 1.690039
O 3.199087 -1.983233 -0.570162
H 2.258123 -1.758461 -0.780688
H 3.141065 -2.778674 -0.032012
H -0.833223 -2.013456 -0.819234
H 0.580178 -0.883193 0.153829
H -2.476654 -0.811041 -1.786798
H -1.563298 0.467426 -1.795865
H -1.931794 -1.854637 1.182180
H -1.006875 -0.982489 2.060488
H 1.535815 0.080826 1.720342
H 0.184436 0.826392 1.365179
H 1.534786 2.709649 0.090522
H 1.447944 1.734223 -1.075854
H 0.448002 -0.387681 -1.333113
H 0.179776 1.237052 -2.744390
H -1.566556 1.923333 0.757406
H -0.514082 1.859661 -0.393098
H -3.076236 0.700349 -0.049449
H -2.829235 0.218024 1.392100
O 3.230108 0.307946 1.146706
H 3.390004 -0.468006 0.577098
H 2.977612 1.025440 0.522397

31
oh2.10h2o, E= -839.807519
O 2.262515 -1.223238 -1.489316
O 2.218220 1.366357 -1.309536
O 2.277731 1.402801 1.402997
O -0.348922 2.018450 1.258205
O -1.867393 0.020986 1.923388
O -0.451628 -2.078753 1.440116
O -0.423104 2.079055 -1.194048
O -0.371915 -2.180064 -1.355863
O -2.004270 -0.029977 -1.909524
O -3.937707 -0.038187 -0.128578
O 2.287815 -1.442527 1.351118
H 2.245142 -0.208472 -1.481426
H 2.929453 -1.483277 -2.129968
H 2.406683 -1.480760 0.384999
H 1.350135 -1.683222 1.483003
H -1.427088 0.742352 -1.697195
H -3.217731 -0.044794 -0.855715
H 1.311789 1.635433 1.457998
H -0.555584 2.832090 1.726689
H -1.433684 -0.824229 -1.783518
H 2.380801 1.473379 -0.335147
H 1.285528 1.668634 -1.410827
H -0.701460 2.937922 -1.522733
H -0.426494 2.117963 -0.093372
H -0.448864 -2.250415 -0.383652
H 0.549497 -1.892029 -1.505545
H -1.011448 -1.250982 1.642906
H -0.769518 -2.771364 2.025293
H 2.319928 0.436483 1.547749
H -2.626821 0.057072 1.315110
H -1.290986 0.828989 1.703686

31
oh2.10h2o, E= -839.805688
O 3.968600 -0.363636 -0.644166
O 2.238005 0.236993 1.599060
O 0.843201 -1.731534 0.103652
O 1.162460 -0.039909 -1.738643
O -0.313099 -1.005454 2.487605
O -2.400015 0.520671 1.541322
O -1.355378 2.552905 0.321607
O -1.422658 1.078371 -2.071559
O -3.045336 -0.792704 -0.774101
O 1.395785 2.282925 -0.087764
O -1.244857 -3.015991 -0.723044
H 3.704578 -0.154025 0.268056
H -2.540675 -0.178861 -1.350653
H -2.739550 -0.014635 0.782604

H -1.698670 -0.032135 1.959097
H -2.525962 -1.619788 -0.776693
H -0.402534 -2.527226 -0.378324
H -0.525188 0.699515 -1.991518
H -1.466615 1.742314 -1.358793
H 0.421249 -0.420480 2.724185
H 0.018657 -1.411806 1.650904
H 1.395152 1.656427 -0.830568
H 0.452040 2.456435 0.097283
H 1.762875 -0.442381 1.075463
H 1.993118 1.075581 1.152545
H 3.170664 -0.132237 -1.141530
H 1.027458 -0.777429 -1.006450
H -1.814598 3.352304 0.591173
H -1.752993 1.786409 0.856358
H 1.583170 -2.348018 0.137614
H 1.324165 -0.491056 -2.572512

31
oh2.10h2o, E= -839.805321
O 0.692691 -2.579071 0.461155
O -1.496287 -0.835785 -2.056245
O -0.586954 0.387220 1.737553
O -2.241538 2.447852 0.705787
O -1.833034 -1.534166 0.531997
O -3.627221 0.273638 -0.216387
O 1.333156 -1.211828 -1.717688
O 0.343350 1.155096 -0.679986
O 1.993793 -0.726277 2.098144
O 3.428330 0.102176 -0.226613
O 2.423368 2.840505 -0.614700
H -1.196370 0.045982 -1.792579
H -1.638545 -1.279453 -1.177875
H 1.168367 -1.814521 -0.933715
H 0.506183 -1.283100 -2.225993
H 1.191671 -0.174964 2.134102
H 2.543398 -0.366999 1.373679
H 0.062787 0.873737 0.230765
H 0.780113 0.352223 -1.050608
H -1.120034 -0.407696 1.399662
H -1.212260 1.133801 1.771199
H -0.243898 -2.258276 0.524206
H -2.310555 -2.252993 0.955259
H -1.544815 2.508955 0.040975
H -2.838971 1.743415 0.356505
H 2.899574 -0.344956 -0.908376
H 3.309869 1.050785 -0.399053
H 1.571168 2.300609 -0.631161
H -3.524807 0.142693 -1.166895
H -3.019530 -0.434365 0.157229
H 1.179413 -2.089750 1.158550

31
oh2.10h2o, E= -839.804372
O 0.356728 -0.110468 0.995580
O 3.587934 -0.018410 -0.648508
O -1.194755 -1.912080 -0.662970
O 0.948048 -3.680361 -0.839709
O 2.122923 -1.870131 0.494380
O 2.294100 1.898905 0.844555
O -0.962089 1.731451 -0.583484
O -2.045558 -1.098305 2.018532
O 0.972970 3.738299 -0.563849
O -3.345564 0.991784 0.711509
O -2.832605 0.009363 -1.890637
H 3.274433 0.025563 -1.556912
H 3.073955 -0.784378 -0.232379
H 1.577965 1.289219 1.113308
H 2.881817 1.318011 0.307862
H -1.997337 -1.699569 1.257914
H -2.596991 -0.354926 1.684985
H -0.413765 1.081671 -0.093334
H -0.386083 2.514397 -0.703972
H -0.618722 -1.214958 -0.301322
H -0.567673 -2.637297 -0.875697
H 1.070379 -0.849413 0.888878
H 2.616931 -2.220439 1.242001
H 1.498769 -2.963691 -0.300146
H -2.558694 1.517147 0.472635
H -3.525550 0.551935 -0.142260
H -2.155520 0.693586 -1.740269
H -2.366069 -0.813045 -1.648935
H 1.556834 3.098790 -0.068303
H 0.757233 4.421658 0.077681
H -0.338967 -0.414653 1.615093

31
oh2.10h2o, E= -839.803492
O -2.038925 -1.544652 -1.038119
O -2.400750 -0.944532 1.309293
O 0.366681 -2.358891 -0.901842
O -2.314881 1.685091 0.985907
O 2.166959 -0.398852 -1.774072
O 0.653330 1.833967 -1.814818
O -2.045578 1.063055 -1.836077
O 3.931379 -0.136388 0.165503
O 1.867998 0.444092 2.136683
O 0.448055 2.455790 0.883005
O 0.229577 -1.788094 1.848982
H -0.614609 -2.073768 -1.033918
H -2.704493 -2.050105 -1.511730
H 3.274447 -0.229219 -0.611593
H 1.630475 0.435225 -1.850274
H -2.272851 1.473259 -0.987076
H -2.036769 0.094274 -1.632232
H -2.392488 0.713549 1.194632
H -2.849524 2.153202 1.632213
H 0.461541 -3.231641 -1.291763
H 1.526481 -1.090621 -1.513076
H -0.281383 1.573841 -1.960909
H 0.408819 -2.089139 0.937023
H -0.697074 -1.479898 1.804903
H -3.121931 -1.428516 1.719252
H -2.318892 -1.265928 0.264659
H -0.484303 2.200392 0.990051
H 0.667050 2.140047 -0.879385
H 0.966046 1.772399 1.368957
H 1.303265 -0.359282 2.091222
H 2.625439 0.257239 1.553494

31
oh2.10h2o, E= -839.803396
O 0.549409 -1.617981 1.960812
O -1.587909 -0.137736 2.072771

O -1.389046 2.288304 1.006264
O 2.858540 -1.008016 0.405088
O 3.252479 1.751612 0.456159
O 0.959725 2.497202 -0.241363
O -0.016965 0.568592 -1.610196
O 1.678503 -1.640040 -1.999726
O -0.423697 -2.483922 -0.487536
O -2.778961 -1.329148 -0.221748
O -2.691787 1.321034 -1.371103
H -0.233035 -0.987006 2.061589
H 0.679525 -2.041072 2.813387
H -0.013063 -2.376789 0.392913
H 0.301733 -2.299180 -1.137557
H 2.284289 2.107992 0.198556
H 0.978320 3.355869 -0.674766
H -2.935088 0.444807 -1.028466
H -1.778782 1.166553 -1.694153
H 3.057838 -0.044672 0.446320
H -2.176888 -0.540261 1.409985
H -1.478334 0.810191 1.768137
H -0.473322 2.424511 0.624503
H -1.954101 2.143771 0.223034
H 2.239322 -1.446710 -1.210621
H 2.109244 -1.141217 1.011891
H 1.257764 -0.779749 -2.188065
H -3.449778 -2.011355 -0.311061
H -1.905349 -1.770216 -0.429367
H 0.417232 1.367243 -1.127122
H -0.209855 -0.061912 -0.904511

31
oh2.10h2o, E= -839.802248
O 0.201459 -0.629932 -0.383538
O -2.119520 -1.398656 -1.432835
O -1.770110 -2.715462 1.077801
O 1.256113 -2.851171 -0.017622
O -2.897701 1.059009 -0.864106
O -1.497941 0.140962 1.430981
O 0.100896 2.392795 1.882890
O -0.532276 2.745688 -0.842963
O 3.472989 -1.016257 -0.706929
O 1.911130 1.554413 -1.392321
O 2.128004 0.465514 1.314314
H -0.852141 -2.932725 0.852703
H -1.180368 -1.147489 -1.200566
H -2.322455 -2.011346 -0.702372
H 2.121143 1.508777 -0.445039
H 1.054927 2.039323 -1.403373
H -0.827631 -0.078165 0.708465
H 1.369666 -0.003865 0.898238
H 2.889807 -0.000797 0.927556
H 0.808164 -1.906411 -0.227123
H 2.866336 -1.770023 -0.589649
H -1.320331 2.180999 -0.934946
H -0.339905 2.754158 0.119361
H 0.975040 1.969934 1.863310
H -2.712997 0.118878 -1.159579
H -3.710553 1.337835 -1.293333
H 0.639317 -0.004330 -0.978452
H 2.992099 -0.393530 -1.272387
H -0.513830 1.632853 1.955742
H -2.233145 0.533414 0.934177
H -1.727485 -1.802718 1.421900

31
oh2.10h2o, E= -839.801865
O 3.141433 -0.372462 -0.389502
O 1.976144 -2.178518 1.461758
O 0.748569 -2.929511 -1.008382
O 0.595208 -0.374939 -1.556495
O -2.032724 0.259045 -1.479305
O -1.380943 2.378993 0.318782
O 0.836709 2.442549 -1.228802
O 2.409220 1.873141 1.012450
O 0.149667 0.012876 1.221851
O -2.534481 -0.003429 1.901669
O -3.646599 -1.376359 -0.187179
H 2.298216 -0.366168 -0.897186
H 2.990122 -1.066697 0.281335
H 1.358355 -1.447916 1.645306
H 1.534846 -2.657074 0.734955
H 0.630724 -1.937830 -1.249421
H 0.767215 1.482064 -1.421911
H 1.486252 2.463666 -0.494108
H 1.662003 1.413313 1.424507
H 2.867919 1.136827 0.543802
H -0.777338 -0.176696 1.491227
H 0.204545 -0.111532 0.254719
H -3.038714 -0.835872 -0.770400
H -4.536630 -1.196428 -0.502271
H -3.023156 -0.589670 1.280266
H -2.696764 0.885856 1.561893
H -1.089245 -0.041685 -1.575908
H -1.964233 1.053354 -0.914703
H -0.687347 2.654564 -0.330570
H -0.849142 1.821211 0.907397
H 0.764738 -0.334381 -2.503685

31
oh2.10h2o, E= -839.801419
O -0.118276 -0.021177 1.274617
O -1.066835 -1.301829 -2.185161
O 0.547028 1.001682 -1.295484
O -3.096876 -0.714655 -0.387211
O -1.313199 3.049687 -1.000222
O -2.196964 1.410294 0.692074
O -2.305172 -1.804373 2.189940
O 0.754822 -2.236858 -0.308983
O 2.088191 1.699858 1.372037
O 4.043718 -0.211563 0.909435
O 2.771098 -0.751764 -1.389492
H -0.564200 -0.470468 -2.180940
H -1.840606 -1.125107 -1.606727
H -1.449466 -1.353434 2.143420
H -2.693380 -1.617174 1.319332
H 1.846526 1.881265 0.453378
H 2.858714 1.093750 1.300988
H 0.429884 -1.625277 0.377514
H 0.080250 -2.145419 -1.016603
H 0.297925 0.550282 -0.462826
H -0.043810 1.787360 -1.340009
H -0.939925 0.564869 1.168324
H -2.716068 1.714933 1.441681
H -1.752842 2.435932 -0.263797

H 3.819162 -0.985594 1.435941
H 3.674124 -0.413768 0.006913
H 2.107623 -1.402998 -1.050032
H 2.213321 0.011484 -1.614962
H -3.986999 -0.581649 -0.723887
H -2.805691 0.176630 0.038718
H 0.605182 0.549958 1.601172

31
oh2.10h2o, E= -839.799932
O -2.914717 -2.953971 0.211218
O -2.957091 -0.504207 -0.734408
O -2.488338 1.418902 0.885969
O -0.277475 0.186081 1.277578
O -1.478453 3.400739 -0.422006
O 0.722605 1.797252 -0.653791
O 3.357036 1.302191 -0.201153
O -0.305662 -2.041435 -0.459959
O 2.331344 -2.300720 0.121834
O 2.382027 -0.262104 1.974248
O 1.521965 -0.466215 -2.037605
H -3.040555 -1.991767 -0.105682
H -2.110188 -0.507577 -1.196009
H -2.876278 0.284298 -0.076365
H -1.178690 0.666748 1.287451
H 1.432731 -0.027275 1.933672
H 2.818746 0.367270 1.367398
H 2.488616 1.734187 -0.355498
H 3.353824 0.592056 -0.855464
H 0.008036 2.470838 -0.749259
H 0.402728 1.229140 0.093170
H 2.411914 -1.690256 0.893108
H 2.407842 -1.706081 -0.643021
H -1.962397 2.658949 0.092231
H -1.237862 4.043396 0.252124
H 1.202925 0.408636 -1.728884
H 0.774558 -1.053359 -1.839911
H 0.549688 -2.405084 -0.133633
H -1.037582 -2.646632 -0.246401
H -0.409688 -0.624101 0.748196
H -3.144303 1.584516 1.567373

31
oh2.10h2o, E= -839.799669
O 0.218277 -0.740244 1.059159
O 3.044565 -0.962506 0.951103
O 2.532420 0.971616 -0.740072
O 0.328978 -0.406615 -1.442362
O -1.031776 1.772378 -1.852195
O -2.441225 -0.924794 -1.353277
O -3.256122 0.574669 0.962150
O -2.214088 -2.035152 1.343423
O 1.850757 -3.045739 -0.604056
O -0.674033 1.865923 0.860152
O 1.630205 3.242003 0.894843
H 0.297255 -0.692055 0.028309
H 1.126944 -0.861604 1.376861
H 3.899188 -0.899152 1.384478
H 2.964702 -0.178322 0.327771
H 1.698580 0.550231 -1.097011
H 2.268786 1.803294 -0.310891
H -1.467326 -0.796812 -1.409053
H -2.759603 -0.277138 -0.696170
H -3.007851 -0.280053 1.367277
H -2.501976 1.164153 1.133547
H -2.380851 -2.049946 0.385057
H -1.293773 -1.704219 1.397680
H 0.716539 2.816841 0.957777
H 2.397056 -2.414331 -0.102819
H 0.952307 -2.792470 -0.363138
H -0.437290 0.966940 -1.903706
H -1.903276 1.410112 -2.051847
H -0.337914 0.954824 1.035962
H -0.857659 1.890576 -0.113767
H 0.722499 -1.103181 -1.977274

31
oh2.10h2o, E= -839.799635
O -1.268443 -1.545361 -1.835261
O 0.854898 -0.039044 -1.711947
O 1.599151 2.389387 -0.962860
O -0.455458 2.774361 0.622355
O -2.662589 1.973443 -0.372665
O 0.330197 0.479364 1.345835
O -3.094154 -0.671504 0.023111
O -1.323458 -1.618508 1.976015
O 0.219421 -2.947861 0.203855
O 2.535214 -1.778034 -0.311862
O 3.058428 0.790901 0.991919
H -0.510667 -0.871358 -1.838747
H -0.306020 -2.728610 -0.585149
H -0.317160 -2.564926 0.950561
H -1.802830 2.337179 0.066369
H -0.490340 3.520871 1.225052
H 3.146131 -0.083561 0.581146
H 2.150159 0.744555 1.362110
H -2.955049 0.308392 -0.067728
H 1.592667 -0.577912 -1.370359
H 1.090375 0.921452 -1.541455
H 0.829750 2.674571 -0.375512
H 2.274058 2.061196 -0.340727
H -2.057650 -1.348104 1.383063
H -2.802989 -1.036933 -0.826378
H -0.761175 -0.813027 1.998461
H 3.100060 -2.461837 -0.681751
H 1.696562 -2.242110 -0.015082
H -0.023714 1.439175 1.197398
H 0.340805 0.128190 0.443223
H -2.538630 2.135650 -1.312459

31
oh2.10h2o, E= -839.799503
O -3.318493 -2.007757 -0.008269
O -0.746061 -1.706925 -0.490480
O -0.269995 0.014724 -2.424281
O 2.214766 -0.208801 -1.749915
O 1.855362 -2.257567 -0.196016
O 2.805168 1.362134 0.202641
O 0.382938 2.317423 0.792108
O -1.507638 2.218204 -0.998605
O -3.114120 0.790679 0.841992
O -0.680541 -0.151562 1.731681
O 1.944169 -0.613252 2.280671
H -2.328889 -2.039821 -0.212091

H -0.642369 -1.077941 -1.270340
H 0.156211 -2.117056 -0.386469
H 2.033170 -1.856905 0.668594
H 2.079862 -1.515617 -0.859695
H -0.676556 -0.711094 0.912453
H -1.578481 0.244856 1.716379
H 0.989645 -0.385119 2.229771
H 2.082764 -0.963099 3.164917
H 2.845108 0.740603 0.943100
H 2.630313 0.773022 -0.617223
H 0.735366 0.007836 -2.246158
H 2.863552 -0.278414 -2.454910
H -0.619203 0.860400 -2.101046
H -1.737020 3.095025 -1.318277
H -0.732762 2.339339 -0.363727
H -2.678154 1.299064 0.128275
H -3.346586 -0.071030 0.452739
H 1.312361 2.087783 0.507575
H 0.087221 1.509775 1.253912

31
oh2.10h2o, E= -839.799222
O -2.114896 0.829948 -1.805728
O -2.563513 -1.466555 -0.678840
O -0.054230 -2.539908 -0.316431
O 0.629869 1.340800 -2.074384
O 1.855321 -1.116836 -1.718958
O 3.728309 -0.520627 0.082592
O 1.836445 0.518726 1.895751
O 0.919658 2.459815 0.479038
O -1.871697 2.089240 0.742531
O -2.370971 -0.537143 1.830612
O -0.172471 -1.142785 1.735528
H -2.289791 -0.092125 -1.418797
H -2.786089 0.978157 -2.476775
H -2.061829 1.765282 -0.155820
H -0.912480 2.267444 0.729809
H 3.054807 -0.803050 -0.601278
H 4.115970 0.277865 -0.288586
H -0.622469 -1.477471 2.516939
H 1.416801 -0.256549 -1.911923
H 1.171971 -1.660118 -1.266601
H -2.575531 -1.176212 0.279052
H -1.742585 -1.998191 -0.728908
H 0.184643 -3.443939 -0.095873
H -0.108523 -1.999555 0.597728
H 0.790890 1.810770 -1.233149
H -0.331327 1.163904 -2.076259
H 1.306145 1.713795 1.059201
H 1.303634 3.284227 0.788010
H -2.076226 0.375573 1.618244
H 2.532571 0.071192 1.372366
H 1.054828 -0.118399 1.918932

34
oh2.11h2o, E= -916.271768
O 2.965304 -0.418485 1.877139
O 0.377832 0.362352 1.653966
O 0.422726 1.849963 -0.720438
O 1.956422 0.232924 -1.769670
O -0.457390 -1.157536 -2.073627
O -2.914554 -0.052893 -1.573138
O -3.173380 -1.367577 0.664166
O 3.628034 1.497836 -0.069325
O 2.326455 -2.054027 -0.265785
O -2.316256 0.941785 2.086026
O -2.323152 2.335578 -0.331513
O -0.458422 -2.033182 0.585543
H 2.836700 -1.138848 1.218558
H 3.375137 0.288072 1.339027
H -2.352664 1.965950 0.579938
H -2.763374 0.867447 -1.254177
H -2.040501 -0.363037 -1.908767
H -1.376115 2.441523 -0.524549
H 1.078428 1.189211 -1.280328
H -1.362864 0.754017 2.180636
H -2.697547 0.110724 1.747995
H -0.441129 -1.583583 -1.192249
H 0.385993 -0.656826 -2.095854
H -0.178383 -1.192692 1.017259
H -1.426580 -2.056916 0.696957
H 3.068907 2.259451 0.117101
H 3.109017 0.984706 -0.747603
H 2.225800 -1.302040 -0.896174
H 1.423530 -2.350577 -0.077415
H 1.307822 0.120200 1.899663
H 0.454320 0.944661 0.868260
H -4.016184 -1.826603 0.709011
H -3.141742 -0.891780 -0.229192
H 2.262467 0.343033 -2.674846

34
oh2.11h2o, E= -916.267860
O 2.991590 -0.344065 1.840146
O 0.422168 0.489898 1.643407
O -0.528006 -1.962956 0.842521
O 2.237038 -2.124244 -0.148938
O 1.744980 -0.056222 -1.838130
O 0.713699 1.956555 -0.620243
O 3.533164 1.351376 -0.321270
O -2.174882 1.256800 1.849403
O -2.110790 2.292898 -0.585038
O -2.988128 -0.219552 -1.562736
O -3.245924 -1.138455 0.877546
O -0.609647 -1.328283 -1.898493
H 2.812974 -1.107197 1.243639
H 3.355267 0.317610 1.214361
H -2.172718 2.009087 0.388481
H -2.856474 0.736414 -1.440935
H -2.090895 -0.583589 -1.821505
H -0.145182 2.301713 -0.900797
H 1.019356 1.218974 -1.250814
H -1.236511 1.019827 2.023574
H -2.628865 0.405767 1.692411
H -0.553485 -1.702449 -0.997219
H 0.247041 -0.819768 -1.980382
H -0.184244 -1.090160 1.149328
H -1.489108 -1.913208 0.979089
H 2.901328 2.072994 -0.203765
H 3.020419 0.747018 -0.920393
H 2.100483 -1.425359 -0.843326
H 1.345583 -2.403446 0.102059
H 1.339765 0.230378 1.916195
H 0.548689 1.042989 0.831703

H -4.089685 -1.579075 1.007873
H -3.218558 -0.824741 -0.080922
H 2.092712 -0.043791 -2.734036

34
oh2.11h2o, E= -916.267420
O 1.222814 0.724790 2.484154
O -1.233006 -0.435950 2.464503
O -3.008058 1.083736 0.846934
O 2.820030 -1.047380 0.918210
O -1.088216 2.585267 -0.640643
O 1.240371 2.527237 0.473875
O 2.996253 0.957789 -1.021443
O 1.171522 -0.637103 -2.506609
O -1.170785 0.441163 -2.377854
O 1.185878 -2.541429 -0.438933
O -1.181642 -2.380391 0.553472
O -2.729395 -1.101751 -0.845937
H 0.320710 0.315071 2.532124
H 3.507166 -1.618512 1.271390
H 1.787802 0.049429 2.068392
H -1.520773 -3.203326 0.916731
H 0.240683 -2.510365 -0.048969
H -1.782779 -0.163391 -1.880507
H -1.147326 1.269158 -1.852349
H -1.177842 -1.189740 1.823750
H -1.885404 0.167884 2.056871
H 0.238982 -0.201469 -2.493275
H -2.386644 1.685441 0.398768
H -3.070921 0.314358 0.243242
H 3.099989 0.271902 -0.331328
H 2.471800 0.515176 -1.712476
H 1.209458 1.947847 1.281642
H 1.894534 2.086849 -0.111557
H -1.253315 3.504096 -0.868071
H -0.175413 2.561185 -0.191711
H -2.068388 -1.736727 -0.227871
H -3.411030 -1.652752 -1.237999
H 2.176294 -1.650378 0.419509
H 1.126292 -2.169566 -1.334144

34
oh2.11h2o, E= -916.267291
O 2.775235 2.037951 -0.387834
O 0.050959 1.862443 -0.589434
O -0.214294 0.712126 1.864421
O 2.607554 0.490891 1.981061
O 2.612112 -1.950637 0.440254
O 0.057036 -0.609973 -1.785130
O 2.835694 -0.332877 -1.912725
O -2.798874 1.868362 -0.653294
O -2.608608 -0.709155 -1.893565
O -2.475346 -1.689213 0.227506
O -2.922775 0.314205 1.654019
O 0.022667 -1.817221 0.773225
H 2.826260 1.620410 0.496492
H 2.923614 1.290524 -1.004946
H -2.781253 0.167209 -1.477519
H -3.032808 -1.997431 -0.495359
H -0.944166 -1.970792 0.507845
H -0.919332 -0.686136 -1.981883
H 0.143951 -1.100199 -0.944600
H -1.857845 2.115360 -0.674120
H -2.937367 1.490278 0.236658
H -0.017181 -0.962783 1.259075
H 1.624176 -2.062025 0.518992
H -0.149698 1.211731 1.013054
H -1.177252 0.636147 2.034586
H 1.894233 -0.400490 -2.159980
H 2.918259 -0.969648 -1.181381
H 2.702116 -0.378759 1.554313
H 1.658009 0.571533 2.182340
H 0.995857 2.146277 -0.561970
H 0.063609 1.028229 -1.115319
H -3.633502 0.161639 2.281259
H -2.836435 -0.541980 1.053684
H 3.005887 -2.784311 0.710748

34
oh2.11h2o, E= -916.267196
O -0.493169 -0.259013 -1.815099
O 1.773402 0.324132 -0.776946
O 2.649647 2.778340 -1.188642
O -1.514643 1.690508 -0.331636
O 3.271803 -2.022541 -0.760761
O -1.582143 -0.136383 1.624531
O -0.494354 -2.189480 0.115810
O 1.865904 -2.353640 1.512829
O -2.944207 -1.409318 -1.414568
O 0.595408 3.108347 0.831660
O -4.087079 0.398099 0.392720
H -1.124405 1.087559 -1.025191
H -0.816491 2.315283 -0.041514
H 0.858570 2.363842 1.402740
H 1.317238 3.184289 0.182517
H 2.862441 -2.663826 -1.350190
H 2.875257 -2.214951 0.130287
H 0.883758 0.093095 -1.241006
H 2.368829 -0.447255 -0.889069
H -0.368239 -1.637473 -0.693635
H -0.954681 -1.565967 0.719751
H -2.115639 -0.911848 -1.640297
H -2.591798 -2.150159 -0.905020
H -1.523693 0.567171 0.921718
H -2.545414 -0.211178 1.721692
H 1.835108 -1.469251 1.917958
H 0.999438 -2.412936 1.042544
H -3.507296 1.138385 0.155967
H -3.828904 -0.283374 -0.264782
H 2.374420 1.805596 -1.106719
H -0.416939 -0.255474 -2.773492
O 1.186592 0.456063 1.904458
H 1.347007 0.369324 0.934361
H 0.254137 0.218220 2.046528

34
oh2.11h2o, E= -916.265162
O -1.701001 2.472828 -0.555715
O 0.796390 3.834824 0.067904
O 1.689605 1.299320 -0.296107
O -0.357405 0.181322 -1.305253
O 0.610621 -2.386264 -0.951280
O -1.804320 -3.045812 0.315904
O -2.980952 -0.988686 -1.170262

O -3.443424 0.873996 0.915739
O -1.017749 -0.532153 1.364329
O 1.605737 0.157061 2.086675
O 4.018254 -0.182589 -1.045152
O 2.793024 -1.931605 0.517726
H -1.236228 1.663699 -0.879316
H -1.001765 3.003864 -0.139557
H 0.770111 4.238291 -0.805291
H 1.232949 2.961663 -0.074426
H 0.889754 0.838636 -0.770783
H 2.510537 0.948996 -0.691407
H -2.102024 -0.588958 -1.323665
H -3.375634 -0.391006 -0.502256
H -2.721352 0.413460 1.377445
H -2.967536 1.595429 0.452141
H -0.172464 -0.331292 1.806046
H -0.855496 -0.329115 0.423290
H 3.611761 -0.915922 -0.464214
H 1.669087 0.670709 1.229957
H 1.821909 0.770487 2.794499
H 0.285410 -1.474049 -1.140085
H -0.168363 -2.827192 -0.550816
H -2.369326 -2.507338 -0.279451
H -1.553302 -2.382726 0.982583
H 1.979281 -2.204732 0.008899
H 2.457137 -1.397981 1.256873
H -0.374694 0.227138 -2.266535

34
oh2.11h2o, E= -916.264805
O -0.536569 -0.335885 -1.829127
O 1.677701 0.495507 -0.733987
O 1.868552 3.135585 -1.146819
O -1.697155 1.420765 -0.174295
O -1.509432 -0.569280 1.642347
O -0.128811 -2.357906 -0.000565
O -2.914291 -1.458830 -1.693253
O 2.226742 -2.231880 1.380486
O 3.522279 -1.583615 -0.882738
O 1.149223 0.413637 1.965174
O 0.175671 3.014715 1.099072
O -4.111419 -0.063312 0.513416
H -1.252946 0.929729 -0.914837
H -1.071497 2.086641 0.191067
H 0.571923 2.288178 1.613795
H 0.835756 3.218683 0.406887
H 3.193363 -2.251757 -1.492540
H 3.181537 -1.873416 0.006070
H 0.837818 0.151353 -1.185230
H 2.385010 -0.165008 -0.893962
H -0.158889 -1.768607 -0.787221
H -0.717958 -1.895041 0.629043
H -1.974571 -1.054487 -1.727292
H -1.556487 0.172479 0.984619
H -2.449780 -0.735933 1.804828
H 2.077876 -1.392075 1.848562
H 1.371512 -2.376239 0.903901
H -3.553428 0.710071 0.330691
H -3.840274 -0.674805 -0.197064
H 1.912599 2.147717 -1.065942
H -0.403040 -0.283534 -2.780344
H 1.305809 0.408580 0.990854
H 0.264563 0.029626 2.093057
H 1.261165 3.291837 -1.876623

34
oh2.11h2o, E= -916.263895
O -0.068866 0.065250 1.120111
O 1.887002 -0.518102 -2.056243
O -1.412666 -1.859309 -0.315577
O 1.040452 -2.957149 -0.981872
O 1.676559 -1.640689 1.443304
O 3.469914 -0.080316 0.316624
O 1.804413 1.969902 1.424449
O -0.177696 1.211942 -1.282257
O -2.826014 -0.866322 2.035178
O 0.830317 3.685999 -0.393811
O -4.188525 0.974418 0.232942
O -2.658735 0.111769 -1.754019
H 1.146184 0.112065 -1.928293
H 2.524166 -0.280510 -1.360907
H 1.064860 1.300979 1.420724
H 2.562316 1.469334 1.079655
H -2.509964 -1.496941 1.368659
H -3.386501 -0.252158 1.528403
H -0.187341 0.833168 -0.357510
H 0.127967 2.133950 -1.172721
H -0.879124 -1.160539 0.135913
H -0.718081 -2.454655 -0.671097
H 0.865067 -0.918995 1.374503
H 1.711928 -1.943691 2.354357
H 1.330380 -2.176818 -1.504765
H 1.317279 -2.719528 -0.077259
H -3.642081 0.678225 -0.571986
H -1.848417 0.664880 -1.780015
H -2.309995 -0.736394 -1.396665
H 1.277310 3.127917 0.298986
H 0.113049 4.125402 0.073295
H 4.369247 -0.416633 0.353039
H 2.894172 -0.735734 0.806500
H -0.831657 0.067531 1.716531

34
oh2.11h2o, E= -916.263147
O 2.186285 1.211148 2.155042
O -0.293116 0.346139 1.383002
O 0.399212 -2.141082 0.688843
O 3.105720 -1.234826 1.028829
O 2.924440 -0.045165 -1.558027
O -0.660401 1.671088 -0.948911
O 2.025953 2.361363 -0.380774
O -3.046223 0.070722 1.743637
O -3.437329 1.805169 -0.497327
O -1.708073 -0.245792 -1.557420
O -2.323731 -2.183723 0.091663
O 0.723837 -1.386717 -1.944448
H 2.613222 0.365138 1.911093
H 2.259754 1.757831 1.343276
H -3.437238 1.269503 0.321982
H -2.460797 0.370974 -1.492690
H -0.114880 -0.884989 -2.024491
H -2.503770 2.075553 -0.590043
H -0.467195 1.488431 -1.875186
H -2.096081 0.278019 1.845500

H -3.017952 -0.800269 1.313485
H 0.650495 -1.777163 -1.042472
H 2.065054 -0.514119 -1.796365
H 0.159171 -1.228484 1.007763
H -0.473314 -2.564824 0.612516
H 1.063862 2.229362 -0.513149
H 2.428717 1.619845 -0.868696
H 3.168555 -0.875634 0.124492
H 2.301690 -1.780120 1.019971
H 0.510248 0.736591 1.803586
H -0.384782 0.774177 0.497211
H -2.917723 -2.736768 -0.423752
H -2.056377 -1.425510 -0.525521
H 3.536752 -0.204139 -2.281376

34
oh2.11h2o, E= -916.263089
O 1.122897 -2.455253 0.759883
O -1.136650 -2.451437 -0.350095
O -3.006762 -0.961042 1.075647
O 2.923103 -1.157306 -0.752365
O -1.232043 0.766936 2.496031
O 1.156889 -0.230435 2.459156
O 2.864185 1.179675 0.921477
O 1.264729 2.598998 -0.726588
O -1.316629 2.630201 0.358636
O 1.215360 0.466389 -2.481520
O -1.166984 -0.732561 -2.284510
O -2.725506 0.736013 -1.055208
H 0.184483 -2.452546 0.302595
H 1.253252 -3.335270 1.122978
H 3.438325 -1.767970 -1.285762
H 2.289404 -1.703486 -0.222149
H -1.475129 -1.070006 -3.130265
H 0.317099 0.031937 -2.447125
H -1.830376 1.990380 -0.185677
H -1.300800 2.240076 1.247643
H -1.128065 -1.842481 -1.167930
H -1.826731 -2.058402 0.229716
H 0.363584 2.673052 -0.346713
H -2.515912 -0.468154 1.755478
H -3.069239 -0.328709 0.323510
H 3.129024 0.473384 0.310913
H 2.304447 1.785588 0.364526
H 1.137675 -1.064218 1.948293
H 1.801410 0.350697 1.971948
H -0.277603 0.390245 2.525059
H -2.068788 0.120888 -1.658573
H -3.408175 1.092914 -1.628919
H 1.791111 -0.106215 -1.950396
H 1.190286 1.926872 -1.443492

34
oh2.11h2o, E= -916.262929
O -1.190563 0.305040 -2.412867
O 1.021904 -0.810050 -2.317143
O 1.067508 -2.465427 -0.369122
O 2.753088 -1.101505 0.850152
O -1.243917 -2.410732 0.978941
O -3.010155 -1.047744 -0.912782
O 2.946079 0.794432 -1.145155
O -1.190611 -0.185725 2.623517
O -2.786950 1.156195 0.919369
O -1.213125 2.528273 -0.768328
O 1.238840 2.633079 0.164174
O 1.402727 0.803330 2.342666
H -0.230140 -0.178834 -2.430911
H -3.695039 -1.455501 -1.449479
H -2.372116 -0.628120 -1.541487
H 1.322969 -3.361574 -0.605456
H -0.374097 -2.464273 0.491753
H 1.892219 0.080357 1.894787
H 1.334250 1.498920 1.664688
H 1.019453 -1.534670 -1.583268
H 1.741558 -0.190587 -2.051920
H -0.274211 0.166421 2.594894
H 2.419118 1.510457 -0.743628
H 3.056513 0.140849 -0.421127
H -3.084378 0.406234 0.378796
H -2.213553 0.735620 1.617452
H -1.241355 1.963131 -1.561362
H -1.816450 2.079367 -0.112405
H 1.426354 3.565305 0.302789
H 0.300455 2.586395 -0.192194
H 2.057824 -1.750070 0.367404
H 3.407706 -1.646846 1.293041
H -1.877480 -2.075328 0.326168
H -1.158194 -1.040575 2.134096

34
oh2.11h2o, E= -916.262650
O 0.029125 -2.595427 -1.032332
O -2.682078 -2.109973 -0.168875
O -2.272629 0.106165 -1.328415
O 0.361781 0.055771 -1.561968
O 1.094097 2.651251 -0.869530
O 3.048086 1.508097 0.801331
O -0.586464 2.653858 1.257916
O 3.048371 -0.572456 -0.986197
O 2.060703 -2.511618 0.822650
O 0.489891 0.010019 1.234651
O -1.540684 -1.732252 2.199129
O -2.937712 2.425588 0.017178
H 0.147225 -1.649931 -1.311452
H -0.909109 -2.671242 -0.800981
H -3.540941 -2.476591 -0.393061
H -2.590543 -1.199308 -0.642397
H -1.274728 0.158490 -1.451590
H -2.552193 0.922636 -0.858211
H 2.125892 -0.362517 -1.255026
H 2.942485 -1.369683 -0.431442
H 1.732366 -1.728892 1.289233
H 1.323027 -2.720714 0.206402
H -0.214249 -0.585122 1.558250
H 0.449195 -0.004230 0.256029
H -2.907100 3.151953 -0.613122
H -2.106058 2.530977 0.559874
H -2.046834 -1.944921 1.357065
H 0.806073 1.762751 -1.190397
H 1.909008 2.450366 -0.364671
H 3.231768 0.772682 0.170207
H 2.338154 1.144781 1.348755
H 0.004366 2.841800 0.485469
H -0.288162 1.759614 1.506785

H 0.520472 0.104906 -2.510032

34
oh2.11h2o, E= -916.262558
O 0.435569 1.456070 2.402980
O -0.090951 3.013268 0.051299
O 1.914492 2.561009 -1.623372
O 2.756771 0.294988 -1.133057
O 1.451470 -1.810742 -1.642225
O -2.614745 1.748201 -0.043478
O -1.919921 0.145807 2.085021
O -1.445835 -2.231202 1.216620
O -1.298916 -1.990950 -1.545758
O -2.843898 0.067124 -2.252549
O 1.324906 -2.750139 1.003682
O 2.431049 -0.200236 1.552276
H 1.183019 0.856509 2.153184
H -0.116071 3.972189 0.116342
H 0.332422 2.057841 1.644758
H 3.695867 0.207443 -1.323632
H 2.292365 1.549811 -1.448596
H -0.331360 -1.863718 -1.654718
H -1.422883 -2.139186 -0.586035
H 2.588821 0.033825 0.605649
H 2.096632 -1.118888 1.514961
H -2.222577 -0.679267 -2.027951
H -3.715607 -0.338448 -2.268362
H 0.372951 -2.616738 1.171296
H 1.425277 -2.576027 0.047786
H -1.758316 2.210742 -0.113956
H -2.695254 1.206045 -0.856401
H -1.062242 0.587072 2.317578
H -2.280020 0.699208 1.351777
H -2.014865 -2.822851 1.715793
H -1.626313 -1.290755 1.568035
H 1.983972 -0.932441 -1.472419
H 1.846551 -2.232713 -2.409479
H 0.627710 2.804758 -0.617117

34
oh2.11h2o, E= -916.262295
O 2.487912 -0.665457 -1.960338
O 3.094520 1.674918 -0.323059
O 0.448288 1.637028 -0.157915
O -0.213691 -0.167858 -1.771382
O 3.554654 -1.003065 0.598127
O -0.487687 -0.024147 1.811238
O 1.458496 -1.925333 2.168681
O -0.534054 -2.106947 0.034960
O -3.236201 -2.572958 -0.560649
O -2.854093 0.030358 -0.982797
O -2.720019 1.606256 1.341954
O -1.163623 3.732132 0.056520
H 1.503586 -0.580353 -1.890359
H 2.811783 0.227349 -1.768757
H 3.408132 2.582969 -0.311885
H 2.101203 1.719017 -0.223392
H 0.177332 0.981209 -0.925728
H 0.202773 1.119531 0.645777
H -0.372042 -1.530447 -0.757970
H -1.426765 -2.480763 -0.100721
H -3.215241 -1.582278 -0.680347
H -3.365117 -2.921730 -1.447614
H -2.907938 0.606456 -0.191580
H -1.902802 0.020688 -1.255478
H -0.514173 2.969377 -0.102637
H -2.305124 2.430160 1.034927
H -1.997399 1.093296 1.748988
H -0.717629 -0.737022 1.166872
H 0.250485 -0.461620 2.285503
H 0.892809 -2.281445 1.462291
H 2.264938 -1.620823 1.696696
H 3.206279 -1.188807 -0.299131
H 3.589075 -0.032713 0.585910
H -0.360194 -0.051466 -2.714085

34
oh2.11h2o, E= -916.261426
O -1.851551 2.341606 -0.604271
O -0.398747 0.051229 -1.370891
O 1.601990 1.403060 -0.164327
O 1.580365 -0.261426 2.113323
O 0.403700 3.757177 0.340834
O 0.623527 -2.279180 -0.961147
O 3.168818 -1.883739 0.349006
O 4.194456 0.394996 -1.086998
O -3.537180 0.764791 0.876040
O -1.803938 -2.972053 0.339707
O -2.995451 -1.062898 -1.205286
O -0.932857 -0.578496 1.316904
H -1.334134 1.571581 -0.923648
H -1.189260 2.942237 -0.211215
H 0.737197 4.366908 -0.324048
H 0.922683 2.922412 0.204653
H 0.903820 0.892304 -0.661177
H 3.379547 0.896725 -0.928207
H -2.126145 -0.646882 -1.392296
H -3.402126 -0.457100 -0.551248
H -2.835761 0.345097 1.396806
H -3.056187 1.485378 0.409203
H 0.617035 -0.439913 1.932875
H -0.865154 -0.188328 0.424973
H 4.008961 -0.453949 -0.646169
H 1.750405 0.895094 0.655755
H 1.635335 0.125774 2.991151
H 0.233474 -1.337609 -1.186322
H -1.012702 -3.129206 -0.202472
H -2.388681 -2.442803 -0.256089
H -1.239816 -1.503174 1.141371
H 2.407122 -2.125727 -0.206605
H 2.766439 -1.405018 1.092128
H -0.337117 0.285657 -2.302561

20
oh.6h2o, E= -534.645268
O -2.227794 0.492702 -0.835302
O 0.356242 -0.366316 -1.595157
O -1.161192 -2.072016 -0.152103
O 0.238167 -0.868585 1.965082
O 1.142853 1.992851 -0.595415
O 2.312606 -0.704968 0.028497
O -0.873156 1.664677 1.371766
H -1.367265 0.292832 -1.263642
H -1.952998 0.976318 -0.030198

H -0.177121 1.949441 0.745750
H -0.533052 0.810783 1.713736
H 1.054765 -0.750232 1.441578
H -0.325058 -1.393866 1.355701
H -1.904016 -1.445282 -0.199031
H -0.527955 -1.613511 -0.765728
H 1.916283 1.605884 -0.167385
H 0.777089 1.209395 -1.095022
H 2.642860 -1.606595 -0.014305
H 1.577829 -0.640401 -0.687713
H 0.516827 -0.501511 -2.532687

20
oh.6h2o, E= -534.642390
O 0.853640 0.122690 1.462716
O 1.875842 1.501430 -0.675858
O -1.500545 1.352820 1.121525
O -2.903967 -0.990088 -0.175332
O -0.199736 -1.554159 -0.141735
O -0.879817 0.839333 -1.547225
O 2.729031 -1.210532 -0.156203
H 1.586047 1.178876 0.210854
H 1.054358 1.471420 -1.192050
H 2.216869 -0.866156 0.608698
H 2.731054 -0.411259 -0.709176
H -0.609406 0.953615 1.347437
H -2.101092 0.594114 1.066155
H 0.153395 -0.958306 0.613166
H 0.617828 -1.923209 -0.503326
H -2.746979 -0.342454 -0.876316
H -2.038680 -1.443230 -0.128921
H -0.563749 -0.035698 -1.245142
H -1.123822 1.264517 -0.696207
H 1.028594 0.025808 2.401728

20
oh.6h2o, E= -534.642290
O 0.296271 -1.188676 -1.578275
O 1.500532 -1.173988 0.995951
O -0.737837 0.231289 1.439544
O 3.148855 0.697759 -0.036456
O -2.576251 1.394730 -0.210402
O 0.417592 1.489765 -0.593891
O -2.057711 -1.521426 -0.077525
H -0.561454 -1.457719 -1.184821
H -1.582249 -1.041069 0.654704
H -2.579452 -0.794838 -0.449757
H -1.908590 1.754566 -0.807670
H -2.009427 1.040678 0.523709
H -0.840589 0.498179 2.356023
H 0.317519 -0.223752 -1.410494
H 1.378638 1.500210 -0.447215
H 1.208891 -1.466038 0.110230
H 0.691009 -0.673111 1.306429
H 2.620432 0.018030 0.466059
H 3.290645 0.276099 -0.890449
H 0.043020 1.133142 0.261671

20
oh.6h2o, E= -534.642096
O 2.317781 -0.732953 -0.397308
O 0.688537 -0.904176 1.952873
O -0.398052 1.703435 1.351705
O 1.025263 1.756923 -0.854803
O -0.129516 -0.393439 -1.711153
O -1.078390 -2.057231 0.143037
O -2.387985 0.492379 -0.415543
H 1.522714 -0.793573 -0.976404
H 1.937385 -0.851881 0.495130
H 0.050180 -1.425615 1.413903
H 0.332461 0.000268 1.914140
H -0.356849 2.550441 1.803194
H 0.164424 1.800122 0.519600
H 1.815647 1.239535 -0.616570
H 0.501686 1.027455 -1.335511
H -1.852278 -1.482925 0.253711
H -0.640919 -1.600029 -0.631162
H -1.924752 0.984649 0.280355
H -1.650580 0.232079 -1.024492
H -0.200218 -0.600026 -2.646356

20
oh.6h2o, E= -534.641771
O -0.112225 -0.100849 2.046432
O 1.146487 -1.794865 0.227531
O 2.490827 0.096000 -0.622996
O 1.013548 1.804640 0.371140
O -1.128685 -1.458190 -1.322390
O -2.555892 0.015418 0.693357
O -1.129364 1.431471 -1.371004
H 0.279317 0.691765 1.621812
H -1.779982 -0.043807 1.294288
H -2.286718 0.703574 0.056203
H -0.987293 0.490143 -1.586840
H -0.371550 1.671259 -0.796348
H 1.498577 2.608846 0.570944
H 1.710711 1.099099 -0.079198
H 1.783820 -1.007573 -0.163850
H 2.411626 0.134270 -1.581973
H -1.759858 -1.205911 -0.621944
H -0.312308 -1.693485 -0.832114
H 0.321407 -0.830279 1.556277
H 1.694686 -2.566907 0.386190

20
oh.6h2o, E= -534.641114
O -0.306527 -0.279918 2.018657
O 0.991054 -1.812175 0.283610
O -0.958284 -1.291787 -1.431607
O -2.633179 0.087039 0.530103
O -0.900214 1.517069 -1.382372
O 2.429040 -0.042360 -0.656969
O 1.134553 1.822726 0.630526
H 0.153352 0.544724 1.795160
H -1.885911 -0.068550 1.153183
H -2.286191 0.774423 -0.063832
H -0.791044 0.554587 -1.547584
H -0.237163 1.732420 -0.703696
H 1.704037 2.575703 0.807331
H 1.705430 1.137872 0.152069
H 1.886661 -0.878071 -0.239495
H 2.112911 0.007275 -1.565469
H -1.702683 -1.138327 -0.824651
H -0.221389 -1.599539 -0.827399

H 0.176167 -0.957942 1.461429
H 1.334280 -2.689329 0.467371

20
oh.6h2o, E= -534.641072
O 0.460420 0.037369 1.443421
H 0.360608 0.008413 2.397690
H -2.640010 -1.003560 -0.091598
O -3.324863 -0.326386 0.026199
H -2.796753 0.488567 0.085352
H 1.451480 1.180043 0.775694
O 1.828295 1.850811 0.132374
H 1.100745 2.480376 0.056730
H -0.670254 1.108931 0.680930
O -1.202337 1.676651 0.059629
H -0.901509 1.384845 -0.812228
H 0.176375 -0.467861 -1.356510
O 0.732564 0.135243 -1.885739
H 1.204511 0.703705 -1.245109
H 1.682947 -1.130559 0.801783
O 2.176854 -1.761513 0.224237
H 2.161694 -1.317778 -0.633680
H -0.396753 -1.022158 0.594505
O -0.752571 -1.624315 -0.135121
H -0.079981 -2.315849 -0.173560

20
oh.6h2o, E= -534.640928
O 0.356469 -0.259832 1.991055
O -1.310234 1.360994 0.761358
O 0.468469 1.869076 -1.021762
O 2.764947 0.023096 0.306061
O 1.068183 -0.762344 -1.631313
O -2.734629 -0.308755 -0.555435
O -0.644683 -2.054051 0.102630
H -0.299418 -1.556270 0.878839
H 1.186833 -0.038324 1.538963
H 3.250364 -0.730890 0.653384
H 2.226634 -0.341109 -0.449588
H 0.704327 0.152259 -1.604748
H 0.435199 -1.305846 -1.102074
H -1.480162 -1.585291 -0.113553
H -2.248660 0.399390 0.011046
H -2.499795 -0.074054 -1.458812
H 1.285860 1.883464 -0.510334
H -0.267000 1.750123 -0.317163
H -0.306711 0.410006 1.636134
H -1.735639 2.091072 1.217162

20
oh.6h2o, E= -534.639497
O -0.149694 0.001184 1.969950
O -1.034267 -1.775464 0.002024
O -2.699858 -0.023390 -0.551075
O -1.079871 1.761467 0.009783
O 1.425158 1.503748 -1.161814
O 1.384030 -1.363185 -1.327352
O 2.485583 -0.103723 0.997096
H -0.461136 -0.765930 1.447797
H 1.634181 -0.051759 1.482189
H 2.290786 -0.741244 0.282860
H 1.334534 -0.416516 -1.560300
H 0.510441 -1.557964 -0.924346
H -1.420528 -2.653254 -0.047845
H -1.799617 -1.064906 -0.277131
H -1.830163 1.030218 -0.274751
H -3.384953 -0.032242 0.125950
H 1.926381 1.214148 -0.377000
H 0.514958 1.645008 -0.821053
H -0.471797 0.760626 1.442712
H -1.491730 2.628709 -0.007971

20
oh.6h2o, E= -534.638951
O 1.961100 1.282947 1.138539
O 2.254535 -0.262660 -1.533591
O 0.375671 -2.143528 -0.626910
O -4.144580 0.441547 -0.136649
O 0.427688 1.471559 -0.868977
O -1.458851 -0.021273 0.067298
O 0.495222 -0.971577 1.985076
H 1.328417 1.482783 0.342760
H 1.077796 -0.215572 1.761301
H 0.584414 -1.571500 1.219508
H -0.392769 -1.543208 -0.628327
H 1.089653 -1.602685 -1.028680
H 1.538367 0.444856 -1.459771
H 2.779457 -0.087409 -0.743228
H 0.260830 2.296231 -1.330033
H -0.802843 0.636014 -0.350123
H -1.051556 -0.245141 0.923563
H -3.170806 0.366953 0.009108
H -4.333263 -0.318024 -0.695495
H 1.806023 1.980588 1.781120

20
oh.6h2o, E= -534.638741
O 0.867232 -1.724684 -0.092636
H 1.059801 -2.660060 0.011573
O 0.182425 -0.173028 1.943176
H 0.447081 -0.852148 1.252358
H 0.500230 0.660709 1.566922
O 2.749854 -0.198077 -0.554252
H 3.325556 -0.325321 0.206535
H 1.965274 -0.936955 -0.413939
O 1.212499 1.844474 0.057617
H 1.832387 1.106989 -0.239259
H 1.655789 2.672928 -0.141773
O -1.437602 1.668910 -0.993337
H -1.913873 1.244445 -0.252188
H -0.519051 1.738925 -0.674696
O -2.454758 -0.133394 0.982784
H -1.604957 -0.151593 1.474363
H -2.282111 -0.735014 0.229263
O -1.436695 -1.276415 -1.397645
H -0.559579 -1.493580 -0.972728
H -1.370182 -0.331599 -1.612085

20
oh.6h2o, E= -534.638335
O 1.171751 -1.739033 -0.071925
O 2.934065 0.142040 -0.837274
O -1.636959 1.687183 -1.048895
O -1.452702 -1.392022 -1.242665

O -2.120533 -0.262516 1.047988
O 0.785909 1.854707 0.216104
O 0.320252 -0.166633 1.812785
H 0.881684 -1.222335 0.751230
H 0.367723 -1.752357 -0.618231
H -2.060309 -2.099052 -1.475953
H -1.753437 -1.063114 -0.339585
H -2.200961 0.604774 0.615273
H -1.185313 -0.236994 1.474245
H 0.560798 -0.207244 2.740662
H 0.635209 1.150807 0.924581
H 1.462346 1.454930 -0.354655
H -0.750483 1.838970 -0.633736
H -1.552233 0.818625 -1.465762
H 2.298571 -0.607837 -0.667361
H 3.282141 0.331020 0.040353

20
oh.6h2o, E= -534.636459
O -1.877288 -1.211736 0.236005
H -2.134103 -2.043519 -0.171301
O -3.530360 0.868449 0.008014
H -3.039499 0.003635 0.132411
H -3.036035 1.471773 0.572047
O -0.728735 0.447220 -1.237986
H -1.195963 -0.318406 -0.672747
H -1.477885 0.968993 -1.545255
O 0.504290 -1.229156 1.391377
H -0.442861 -1.332685 1.069750
H 0.593404 -0.262813 1.481197
O 0.746006 1.607771 0.813839
H 1.659506 1.519452 0.491935
H 0.192628 1.303993 0.058978
O 1.589465 -1.090218 -1.140261
H 0.863047 -0.553182 -1.507881
H 1.236903 -1.321393 -0.245003
O 3.359382 0.651485 -0.054571
H 3.479840 0.192127 0.783294
H 2.798945 0.021501 -0.578756

20
oh.6h2o, E= -534.636263
O -0.138822 -1.258340 -0.680348
O -3.015555 -1.097811 -0.516252
O -2.220371 1.188864 0.653176
O -0.048712 1.340276 -0.711656
O 2.770637 1.073663 -0.850090
O 2.403928 -1.574552 0.435320
O 0.425603 0.246099 1.767519
H -0.097164 -0.267278 -0.874180
H -1.087549 -1.464669 -0.631172
H -3.147140 -0.797612 -1.421008
H -2.801677 -0.261063 -0.017357
H -1.398287 1.350435 0.056651
H -1.805996 1.003006 1.505303
H 1.801120 1.214863 -0.787803
H 2.876530 0.151167 -0.570434
H 2.150100 -0.975809 1.153175
H 1.558492 -1.684416 -0.043938
H 0.071059 -0.487051 1.236513
H 0.520335 0.926050 1.069154
H -0.053483 1.946783 -1.456258

20
oh.6h2o, E= -534.635054
O -0.155977 -1.118685 -0.424236
H 0.601030 -1.346165 -0.976897
O 0.584173 0.983184 0.878531
H 0.499008 0.832173 1.824513
H 0.134871 -0.300526 0.117944
O 2.963055 0.974893 -0.034489
H 3.558981 1.261454 0.662867
H 2.012836 1.042532 0.367816
O 2.829375 -1.736414 -0.451729
H 2.971003 -0.764221 -0.357388
H 2.180118 -1.935763 0.231776
O -2.611850 0.216006 -0.990806
H -1.779187 -0.288449 -1.042260
H -2.320634 1.070985 -0.613264
O -1.309969 2.533952 -0.012034
H -0.566121 1.969235 0.388795
H -0.921966 2.890932 -0.816660
O -2.527587 -2.003368 0.930600
H -1.620611 -1.981289 0.582933
H -2.919089 -1.247438 0.463134

23
oh.7h2o, E= -611.109645
O 0.000082 1.025976 -1.474615
H 0.000151 1.544091 -2.284142
O -2.455071 1.139845 -0.329680
H -1.622141 1.101597 -0.873538
H -2.137335 1.582593 0.470109
O -0.000707 -1.494530 -1.563399
H -0.000408 -0.451029 -1.615312
H -0.000463 -1.823862 -2.465418
O 2.455501 1.138340 -0.330141
H 1.622348 1.100637 -0.873730
H 2.138473 1.581451 0.469723
O 0.001537 1.936029 1.222355
H 0.000415 1.766980 0.259731
H 0.000662 1.033223 1.616918
O 2.057740 -1.554554 0.348142
H 2.379568 -0.650908 0.145052
H 1.409519 -1.717081 -0.364433
O -0.000131 -0.706970 2.091937
H 0.772847 -1.074705 1.614138
H -0.773511 -1.073915 1.614149
O -2.058826 -1.553298 0.348583
H -2.380159 -0.649467 0.145516
H -1.410962 -1.716302 -0.364204

23
oh.7h2o, E= -611.108553
O -2.066514 1.564554 0.086953
O -2.479276 -1.209930 -0.068918
O -0.105899 0.973994 2.026852
O 0.283521 -1.754995 1.327609
O -0.110981 -1.290442 -1.292102
O 2.582077 -1.013911 -0.275832
O 1.929888 1.679243 0.241561
O 0.006817 1.186659 -1.771095
H -0.026433 0.152135 -1.666462
H 0.012740 1.377168 -2.712180

H -1.405832 1.598478 -0.632533
H -2.398536 0.644096 0.039707
H -1.659036 -1.278507 -0.639815
H -2.162240 -1.505091 0.792902
H -0.848350 1.269113 1.458363
H -0.041985 0.009335 1.871075
H 0.083436 -1.704401 0.356796
H 1.250903 -1.680456 1.300359
H 1.748408 -1.145370 -0.770069
H 2.545940 -0.060854 -0.050321
H 1.301464 1.525917 0.981395
H 1.348059 1.668120 -0.541599
H -0.065592 -1.951061 -1.987842

23
oh.7h2o, E= -611.107008
O 0.715186 -0.471717 -1.552785
H 0.937156 -0.688518 -2.462477
O 2.679175 0.829546 -0.254491
H 1.987104 0.440495 -0.857567
H 2.891861 0.069642 0.303867
O 1.727448 -1.914409 0.617833
H 1.451617 -1.503834 -0.232854
H 1.169514 -1.465063 1.274372
O -1.210799 -1.791472 -0.552259
H -0.461833 -1.286296 -1.053731
H -0.770061 -2.551706 -0.158082
O -0.977573 1.646500 -1.249585
H -0.372952 0.890718 -1.469891
H -1.782358 1.234375 -0.890533
O 0.526034 2.085357 1.037406
H -0.041681 2.127553 0.234995
H 1.390575 1.792109 0.680952
O -0.464331 -0.344058 1.873251
H -0.103604 0.549184 1.650871
H -0.743470 -0.728493 1.025207
O -3.038375 0.145364 0.205189
H -2.591576 -0.670006 -0.100257
H -2.614405 0.308950 1.058657

23
oh.7h2o, E= -611.106674
O 2.083700 1.637560 0.013439
O 0.097990 1.075604 1.920418
O -1.933614 1.636008 0.004901
O -0.061189 1.133251 -1.724481
O 2.522411 -1.125802 -0.214145
O -0.135894 -1.685778 1.440925
O -2.529636 -1.176097 -0.172404
O 0.069475 -1.433587 -1.191538
H 2.422894 0.724543 -0.095228
H 1.463110 1.550879 0.769164
H 0.081493 0.097105 1.832518
H -0.675020 1.368406 1.404441
H -2.383607 2.455133 -0.217210
H -1.235038 1.488278 -0.718297
H 0.751334 1.432901 -1.261220
H -0.014908 0.128953 -1.658972
H -1.104790 -1.694140 1.460235
H 0.015610 -1.711218 0.453075
H -2.568867 -0.211641 -0.064996
H -1.682655 -1.308092 -0.659658
H 1.650542 -1.260140 -0.696407
H 0.061173 -2.136420 -1.846194
H 2.312787 -1.413823 0.681837

23
oh.7h2o, E= -611.106661
O -0.127962 -1.366119 -1.627815
H -0.364234 -1.884372 -2.400638
O -1.899664 -1.263374 0.065378
H -0.961993 -1.397928 -0.918458
H -2.573205 -1.946848 0.099911
O 0.138803 1.363222 -1.612819
H 0.043483 0.394362 -1.760324
H 0.034647 1.449417 -0.648854
O 2.089290 -1.538295 0.047487
H 1.386551 -1.565622 -0.635250
H 2.384766 -0.607960 0.056849
O 0.007578 -1.151924 1.951695
H -0.757927 -1.304375 1.333966
H 0.790455 -1.424990 1.433317
O -0.028438 1.509234 1.344429
H 0.015698 0.577111 1.679807
H -0.969549 1.616684 1.093485
O 2.526491 1.312926 0.012726
H 2.076462 1.417433 -0.841931
H 1.805120 1.525389 0.633296
O -2.508611 1.298609 0.023183
H -2.386121 0.299604 0.061120
H -2.104049 1.537853 -0.820399

23
oh.7h2o, E= -611.105635
O 0.340913 -0.387719 -1.569448
O 2.266717 1.305269 -0.820316
O 2.145532 -1.718975 -0.216277
O -1.399122 1.474330 -0.676548
O 0.517898 2.066800 1.268405
O 0.658920 -0.644159 2.006949
O -3.408446 -0.099053 0.047042
O -1.121948 -1.808509 0.208486
H -3.834062 -0.253864 -0.801499
H -2.694445 0.573301 -0.159705
H -0.656462 -1.330908 -0.528030
H -1.995691 -1.378711 0.256072
H 1.468069 -1.263969 -0.813729
H 1.707964 -2.535125 0.047701
H -0.779694 0.817138 -1.095024
H -0.863652 1.834616 0.063370
H 1.211528 1.983405 0.582845
H 0.509730 1.173493 1.674294
H -0.100392 -1.053251 1.534285
H 1.393860 -0.873677 1.411039
H 1.524162 0.768930 -1.221192
H 2.822334 0.620885 -0.429177
H 0.283045 -0.586126 -2.507597

23
oh.7h2o, E= -611.104970
O -0.018090 0.008377 -1.559780
O 1.208951 -1.752999 -0.037708
O 1.277911 1.762991 -0.215924
O -2.384101 1.098811 -0.903439

O 3.535281 -0.027744 0.053069
O -0.999244 1.725302 1.462687
O -0.723052 -1.013197 1.955877
O -1.898096 -1.831920 -0.489454
H -1.271780 -1.283085 -1.003987
H -1.650031 -1.627990 0.435955
H -2.789677 0.246207 -0.689656
H -1.507633 0.803805 -1.272144
H 0.784565 1.091600 -0.819323
H 1.357565 2.571462 -0.729873
H -1.613017 1.619765 0.704939
H -0.133667 1.849220 1.026500
H -0.843603 -0.038655 1.917102
H 0.120152 -1.173115 1.495220
H 2.878428 0.690042 0.035550
H 0.800966 -1.081537 -0.669251
H 0.233952 -0.068656 -2.484131
H 2.978618 -0.820647 0.084180
H 0.658680 -2.535383 -0.153708

23
oh.7h2o, E= -611.104777
O 0.613606 0.997314 1.345827
H 0.790457 1.464488 2.166944
O 2.072650 -1.229931 1.135845
H 1.629887 -0.343247 1.268260
H 1.371535 -1.834564 1.412009
O 1.510776 1.875088 -0.900035
H 1.243834 1.580584 0.042348
H 2.413727 2.197190 -0.832152
O -1.321377 2.270705 -0.024307
H -0.702603 1.813434 0.609569
H -0.725689 2.476920 -0.755628
O -0.953098 -1.307949 1.520054
H -0.461775 -0.453923 1.512871
H -0.840225 -1.671737 0.617288
O -2.847138 -0.032638 -0.365300
H -2.536382 -0.453399 0.454228
H -2.408815 0.845224 -0.342194
O -0.906347 -1.948427 -1.232685
H -1.584972 -1.248835 -1.261076
H -0.058973 -1.536694 -1.509291
O 1.630355 -0.853695 -1.619230
H 1.565622 0.118909 -1.547591
H 1.908951 -1.118095 -0.716918

23
oh.7h2o, E= -611.104614
O -0.437006 1.027292 -1.240866
O -1.816350 -0.907527 1.670810
O 1.456498 1.857700 0.473589
O 3.454003 -0.292572 -0.188787
O 0.987641 -1.109024 -1.176948
O 1.014994 -0.639692 1.609865
O -1.991765 -1.336809 -1.158068
O -2.652728 1.371669 0.205236
H -2.091520 -0.006495 1.410096
H -0.851996 -0.841946 1.817489
H -1.536120 -0.485395 -1.329397
H -1.972741 -1.397117 -0.182166
H -1.793650 1.381649 -0.308875
H -3.174332 0.726832 -0.288013
H 0.726750 1.669526 -0.181999
H 2.248554 1.465641 0.075068
H 0.487126 -0.222392 -1.282840
H 0.299906 -1.763336 -1.356833
H 3.147764 -0.486315 0.707802
H 2.749738 -0.695474 -0.732249
H 0.973607 -0.980273 0.692722
H 1.102351 0.327121 1.454627
H -0.437735 1.539684 -2.054076

23
oh.7h2o, E= -611.104284
O 0.000007 -0.000124 -1.786355
O 0.524740 1.964624 -0.208671
O -0.524879 -1.964781 -0.208451
O 0.000114 0.000061 1.757590
O -2.729121 -0.723078 1.165969
O 2.729272 0.723413 1.165702
O -2.277317 1.335813 -0.877667
O 2.277209 -1.335931 -0.877522
H -1.563978 0.818150 -1.317332
H -2.622667 0.721193 -0.207301
H -0.363659 2.349760 -0.216354
H 0.438241 1.251399 -0.926926
H -0.438448 -1.251635 -0.926752
H 0.363587 -2.349780 -0.216093
H 1.563813 -0.818370 -1.317245
H 2.622617 -0.721167 -0.207322
H 0.078664 0.761691 1.144491
H -0.078519 -0.761664 1.144628
H -2.029635 -0.306212 1.696724
H 2.220426 1.392813 0.676975
H 2.029873 0.306545 1.696572
H -0.000107 -0.000035 -2.746186
H -2.220400 -1.392664 0.677366

23
oh.7h2o, E= -611.104046
O 0.019525 -1.170665 2.074025
O 2.302636 -0.204674 0.821391
O 0.870615 2.276672 0.165712
O 0.112009 0.998219 -1.953841
O 1.278078 -1.167861 -1.397077
O -0.863205 -2.250712 -0.331639
O -2.389245 0.235840 -0.712040
O -1.288901 1.131330 1.528645
H 0.911655 -0.887469 1.763659
H -3.335329 0.400669 -0.692011
H -2.025642 0.591815 0.152696
H 0.588965 1.819904 -0.701767
H 1.133252 3.169888 -0.069977
H -1.510661 -1.564169 -0.554770
H -0.327585 -1.687368 1.309380
H -0.552973 1.677412 1.188719
H -0.844788 0.301453 1.854845
H 2.028653 0.719065 0.712457
H 2.025926 -0.630295 -0.050453
H 1.796032 -1.626912 -2.062174
H -0.021269 -1.942563 -0.795514
H 0.621318 0.093517 -1.825864
H -0.819649 0.779855 -1.790633

23
oh.7h2o, E= -611.103703
O -2.490826 -1.186185 -0.504902
O -1.735955 1.448528 -1.213500
O -0.483550 1.725004 1.380546
O -1.055752 -0.780997 1.904932
O 0.265505 -1.791822 -0.104471
O 0.629718 0.186675 -1.931365
O 2.055456 1.595777 0.121804
O 2.763080 -1.102812 0.261153
H -1.555721 -1.484305 -0.539870
H -2.429308 -0.258663 -0.811158
H -0.931905 1.061222 -1.630819
H -1.415079 1.726274 -0.338921
H -0.635034 2.258532 2.165059
H -0.689126 0.770907 1.640473
H -1.891225 -0.884318 1.413728
H -0.432874 -1.252426 1.260200
H 1.195675 0.759333 -1.378243
H 0.478550 -0.616044 -1.368257
H 1.223753 1.705900 0.619509
H 2.387905 0.707263 0.368743
H 1.799620 -1.417023 0.190080
H 3.051971 -1.087299 -0.657552
H 0.261376 -2.742698 -0.246546

23
oh.7h2o, E= -611.102161
O -0.921844 -1.081324 -1.226490
O -0.114981 -2.159279 0.990770
O 0.149859 0.489033 2.034477
O 1.711014 -1.474159 -1.278139
O -1.482919 1.498980 -1.544706
O -2.532511 -0.075027 0.844853
O 2.754666 0.632536 0.157860
O 0.565618 2.419377 0.066428
H -2.067366 -0.491625 0.085571
H -1.807011 0.233282 1.413413
H -0.489376 -1.833241 0.107487
H -0.889518 -2.250796 1.555608
H 0.741216 -1.254329 -1.409854
H 1.672429 -2.139486 -0.580214
H -1.218918 0.534112 -1.567700
H -2.146681 1.496152 -0.841105
H -0.095851 2.171295 -0.618091
H 1.403094 1.986109 -0.179898
H 2.326153 0.478674 1.010563
H 2.439287 -0.107932 -0.412545
H 0.240846 1.193969 1.353765
H 0.232028 -0.373639 1.585801
H -1.371543 -1.643640 -1.863222

23
oh.7h2o, E= -611.101899
O 1.095328 -0.529174 -1.387017
O 2.653951 -1.566024 0.616722
O -0.412356 -1.765805 0.376496
O -1.105181 0.996972 -1.701304
O -2.829907 -0.945370 -0.355788
O 2.301063 1.384553 0.010318
O -1.346575 0.306300 2.024064
O -0.258381 2.339953 0.580691
H 2.225636 -1.243052 -0.215380
H 1.904498 -1.980038 1.062020
H 1.899696 0.779155 -0.673841
H 2.633947 0.741824 0.651636
H -0.280554 0.434259 -1.665029
H -1.809447 0.416390 -1.370663
H -3.373901 -1.603574 -0.796247
H -1.967649 -1.399651 -0.131453
H 0.152701 -1.357976 -0.352704
H -0.476014 -1.082073 1.075662
H -2.162137 0.158829 1.527570
H -0.939518 1.103717 1.596098
H 0.696646 2.125694 0.496827
H -0.627669 2.029146 -0.276857
H 1.340229 -0.893895 -2.241101

23
oh.7h2o, E= -611.101312
O -0.314619 0.918772 -1.009216
O -2.567783 -0.331986 -0.918168
O 0.177309 0.181336 1.560051
O 0.829908 -1.590003 -1.176310
O -1.988231 2.017606 0.885673
O 2.771177 -0.825148 0.675488
O -1.212900 -2.151427 0.756129
O 2.557145 1.736099 -0.617508
H 0.470684 -0.673661 -1.258980
H 0.176197 -2.028518 -0.599531
H -1.840115 -1.627476 0.219177
H -0.724533 -1.459386 1.243001
H 0.222379 0.456600 0.615782
H -0.489149 0.797671 1.900120
H 2.198209 -1.217025 -0.021771
H 2.128944 -0.517100 1.331761
H -1.684630 0.164653 -1.056382
H -2.773823 -0.737320 -1.765043
H 1.600417 1.585353 -0.680434
H 2.868585 0.911377 -0.201630
H -1.337585 1.850933 0.161651
H -2.692629 1.399813 0.652263
H -0.139005 1.452095 -1.789106

26
oh.8h2o, E= -687.568371
O -1.027785 -1.595726 -1.444712
O 1.277495 -0.479180 -1.463815
O -0.813632 -2.070089 1.318778
O -0.137222 1.895223 -1.348563
O 1.941756 -1.842877 0.773696
O 2.762081 0.992238 0.191734
O 0.317452 2.086148 1.356974
O -1.941232 0.458814 1.793982
O -2.609927 0.673438 -0.898361
H -2.241361 -0.179662 -1.191173
H -2.510264 0.641690 0.081689
H 0.368042 1.061034 -1.495617
H -1.073778 1.601169 -1.300662
H 2.652588 1.917147 -0.051381
H 2.227036 0.485775 -0.486462
H -0.095601 -1.160655 -1.503371
H 1.768915 -0.635290 -2.275343
H 0.121495 2.148764 0.390469

H 1.112818 1.532503 1.365422
H -1.164501 1.055284 1.783285
H -1.560231 -0.444601 1.764981
H 1.725436 -1.477590 -0.124871
H -1.015115 -2.026873 0.364084
H 0.164387 -2.036580 1.328939
H -1.037902 -2.321372 -2.073792
H 2.406154 -1.104650 1.186114

26
oh.8h2o, E= -687.567214
O -0.005984 1.642446 -0.692828
O 2.044594 1.446012 0.738352
O 0.697762 -0.767668 -1.462397
O 3.520977 -0.405537 -0.715541
O -1.905990 1.473853 1.260942
O -1.416420 -1.217923 1.864971
O -1.843671 -1.920300 -0.814948
O -2.665947 0.710611 -1.389549
O 1.364591 -1.221948 1.226882
H -1.750540 1.042908 -1.316820
H -2.551548 -0.256527 -1.279845
H -2.587073 1.269913 0.596820
H -1.140482 1.660232 0.663529
H 1.022144 -1.244466 0.308141
H 1.602562 -0.277902 1.321057
H 1.187472 1.605555 0.147909
H 2.104631 2.172423 1.363525
H -1.592097 -0.254952 1.773623
H -0.444228 -1.291105 1.866103
H -1.760525 -1.778089 0.154457
H -0.951665 -1.747491 -1.160010
H 3.197024 0.410691 -0.284357
H 0.403007 0.177719 -1.279827
H 1.653306 -0.708989 -1.620467
H 0.039273 2.384164 -1.303056
H 3.249444 -1.080463 -0.077854

26
oh.8h2o, E= -687.567154
O 0.213193 -1.850295 -0.301590
O -2.589934 -2.150873 -0.030839
O -2.387857 0.349797 -0.968186
O 0.188385 0.299525 -1.746716
O 2.881846 0.566689 -0.907716
O 2.623847 -1.550441 1.058516
O 0.248945 0.072373 1.736083
O -1.822261 2.019660 1.295050
O 0.658088 2.226835 -0.077393
H 0.214393 -1.104414 -0.984799
H -0.700036 -2.192609 -0.279535
H -2.662620 -2.023441 0.920354
H -2.609572 -1.224226 -0.403259
H -1.456085 0.354868 -1.328639
H -2.364571 0.988774 -0.230893
H 2.011352 0.374598 -1.319952
H 2.992083 -0.149095 -0.255910
H 2.144770 -0.971598 1.670938
H 1.888186 -1.923616 0.533753
H 0.112639 -0.647402 1.080916
H 0.530585 0.825799 1.176775
H -1.057117 2.438504 0.862656

H -1.395226 1.267859 1.741357
H 0.397839 1.593022 -0.821653
H 1.625265 2.172873 -0.114667
H 0.214095 0.353942 -2.705115

26
oh.8h2o, E= -687.566997
O 1.610515 2.288916 0.264713
O 1.513071 0.531401 -1.870043
O -1.157680 0.055505 -1.597369
O -0.347584 -2.242469 -0.527971
O -1.127647 -0.883550 1.718342
O 1.557413 0.043898 1.850363
O 2.387156 -1.546040 -0.091596
O -3.296780 -0.094327 0.064292
O -1.134856 1.889318 0.306676
H 0.604535 -0.150138 1.946235
H 1.616938 0.917059 1.388289
H -1.053062 1.202137 0.984506
H -1.135150 1.334064 -0.530047
H -0.654830 -1.453625 -1.059891
H 0.621380 -2.187139 -0.513519
H 1.930988 -0.238505 -1.451806
H 0.534237 0.354273 -1.812323
H 1.711636 1.768485 -0.566312
H 0.640081 2.420745 0.298460
H -0.812220 -1.490377 1.002738
H -2.020895 -0.654711 1.393872
H 2.120303 -0.983035 0.694519
H 3.196505 -2.000996 0.153451
H -2.583539 -0.175493 -0.629859
H -1.486881 0.145413 -2.495785
H -3.258892 0.850625 0.258223

26
oh.8h2o, E= -687.565918
O -1.074742 -0.165361 -1.702619
O 0.899910 -1.955117 -1.300830
O 2.691177 0.053256 -0.657964
O 0.718610 1.823453 -1.451532
O 0.047495 2.223721 1.349338
O 1.556947 0.108482 1.818238
O -0.059800 -2.153450 1.454578
O -2.241169 -1.365506 0.283267
O -2.420022 1.460634 0.126034
H 0.468391 2.161711 -0.576810
H 0.049024 1.103111 -1.629612
H -1.674972 1.821449 0.635172
H -1.986238 1.036742 -0.651928
H 1.012353 -0.703000 1.805857
H 2.083226 0.070237 0.976725
H 2.213225 -0.749882 -0.967621
H 0.669724 -2.234354 -0.400869
H 0.173463 -1.310779 -1.540262
H -1.463510 -0.226748 -2.579444
H 0.593676 1.412058 1.575779
H 0.369470 2.931904 1.912539
H 2.128055 0.776484 -1.020047
H -0.947804 -1.853625 1.071363
H -0.238718 -2.920750 2.003716
H -1.831318 -1.041427 -0.589094
H -2.565302 -0.514029 0.626444

26
oh.8h2o, E= -687.565780
O 0.311391 -1.016496 1.499065
O -0.303541 -1.916753 -0.815386
O -1.946400 0.193110 1.693912
O 1.693813 1.345133 1.515052
O -2.946439 -1.029466 -0.741696
O -2.022316 1.707686 -0.653945
O 0.733129 2.172263 -1.009916
O 1.627228 -0.218835 -2.075095
O 2.929703 -0.981123 0.293416
H 2.071230 -1.125141 0.737266
H 2.657830 -0.721122 -0.612859
H 2.485795 0.909280 1.159890
H 1.106206 0.561831 1.641490
H -2.371131 0.921291 -1.106931
H -1.965495 1.376714 0.264548
H -1.040082 -0.289317 1.655850
H -1.998081 0.608556 2.558537
H 1.038846 2.010498 -0.092694
H -0.240687 2.075416 -0.973474
H 1.344675 0.686426 -1.806887
H 0.882544 -0.785508 -1.802897
H -2.906697 -0.748593 0.186251
H -0.042716 -1.615773 0.133524
H 0.402924 -1.653665 2.213474
H -2.070902 -1.443480 -0.883501
H 0.033194 -2.811578 -0.914844

26
oh.8h2o, E= -687.565057
O -1.301384 -1.598642 0.495329
O 0.435360 -0.183471 1.759802
O 0.583054 -1.444099 -1.396667
O -2.417801 -0.599111 -1.676214
O 2.762710 -1.260332 0.496680
O -2.286649 0.663694 1.893804
O 0.747572 1.174468 -0.603001
O -2.016848 1.967974 -0.633702
H -1.549793 -2.474999 0.799448
H -2.125673 -1.034233 -0.819891
H -1.662805 -0.754412 -2.255751
H -0.187285 -0.856494 1.309302
H -0.214270 0.351624 2.245121
H -0.116826 -1.639473 -0.710505
H 1.421838 -1.593538 -0.921026
H -2.159079 -0.208746 1.464120
H -2.288697 1.273075 1.128852
H -2.298297 1.141317 -1.082072
H -1.045411 1.911560 -0.680315
H 0.635310 0.822029 0.314153
H 0.644556 0.340930 -1.123299
H 2.100102 -0.940950 1.134318
H 3.178175 -0.442786 0.165783
O 3.446358 1.374442 -0.426048
H 2.471444 1.419034 -0.598912
H 3.577739 1.926678 0.350810

26
oh.8h2o, E= -687.564467
O -1.300302 -0.938170 -1.777585
O -2.945724 0.539564 0.009968
O -1.266644 -0.765068 1.950270
O -0.405585 -2.536749 0.234641
O 2.049560 -1.631850 -0.570617
O -1.059391 2.517991 -0.560045
O 0.675448 1.231618 1.445900
O 0.864092 0.678109 -1.258779
O 3.321871 0.842659 0.440384
H -0.516734 -0.336480 -1.694863
H -2.014624 -0.466698 -1.304864
H -2.406704 1.335118 -0.190182
H -2.492994 0.126164 0.766324
H -1.535825 -1.197809 2.764671
H -0.919445 -1.492685 1.338832
H -0.812967 -2.127896 -0.571302
H 0.553646 -2.375001 0.081608
H -0.679274 2.622986 0.323842
H -0.412370 1.885486 -0.968377
H 0.048924 0.562517 1.777508
H 0.754769 1.033083 0.485324
H 1.591204 -0.827742 -0.946494
H 1.240608 1.062162 -2.056094
H 2.700559 0.916605 -0.307783
H 2.720032 1.002386 1.185243
H 2.714600 -1.227023 0.003516

26
oh.8h2o, E= -687.563911
O -0.076624 2.331366 -0.577966
O 1.829021 0.543007 -1.295502
O 0.215647 -1.429276 -1.272220
O 2.223365 1.334577 1.163606
O 2.958054 -1.530015 0.208785
O -2.708721 1.411207 -0.300706
O -2.508787 -0.540730 1.735737
O -2.601825 -1.366145 -1.024293
O 0.322800 -0.764907 1.462833
H 0.564266 1.683368 -0.973579
H 0.297593 2.501703 0.294055
H 0.821604 -0.620992 -1.434605
H 0.824449 -2.172770 -1.203882
H 2.698405 -0.818687 -0.418965
H 2.214539 -1.552400 0.829292
H 2.192425 1.140087 0.178246
H 2.926062 0.755237 1.479426
H 0.819498 0.077353 1.454105
H 0.152966 -0.966091 0.519117
H -1.804782 1.762075 -0.443450
H -2.748500 0.588536 -0.822604
H -1.542529 -0.608987 1.848153
H -2.632839 0.293582 1.238732
H -1.650844 -1.458680 -1.217678
H -2.650737 -1.359262 -0.049245
H 2.294979 0.843260 -2.079307

26
oh.8h2o, E= -687.563463
O -0.358277 1.109161 -1.502014
O 2.014854 0.232885 -1.628373
O -1.676150 -1.174534 -1.529026
O -0.667496 -2.177522 0.695982
O 0.224708 1.851734 1.062014

O -1.640745 0.149585 2.119667
O 2.113028 -1.761212 0.349183
O 2.924725 0.887561 0.956833
O -2.956595 0.953723 -0.229540
H -2.108158 1.227596 -0.632444
H -2.684718 0.703318 0.679287
H -1.093606 -0.368990 -1.677640
H -2.462545 -0.726513 -1.162706
H 1.040850 0.609953 -1.609138
H 2.225694 0.061075 -2.549430
H 0.017316 1.674841 0.110408
H 1.162290 1.601737 1.165194
H 2.855908 0.920307 -0.013751
H 2.734559 -0.056153 1.117074
H 1.179656 -1.949463 0.561414
H 2.066426 -1.220707 -0.464560
H -0.980869 -3.081798 0.784624
H -1.034157 -1.841428 -0.179196
H -0.925472 0.787244 1.879697
H -1.311174 -0.705162 1.794846
H -0.506427 1.793094 -2.161487

26
oh.8h2o, E= -687.562614
O -0.226730 1.363777 -1.213241
O 2.354648 1.755516 -0.207909
O 2.088538 -0.199943 1.533673
O -0.383618 -1.132262 1.481917
O 0.197634 -1.381306 -0.995373
O -2.569479 -1.714341 -0.319409
O -3.110144 1.070071 -0.730951
O -1.423924 1.330907 1.465588
O 2.939193 -1.102529 -1.096108
H -0.097630 0.388613 -1.208996
H 0.659134 1.710055 -0.999654
H 2.733945 2.546321 0.184422
H 2.210075 1.108557 0.547981
H 1.134166 -0.519968 1.615469
H 2.497695 -0.816635 0.902974
H -1.963532 -1.531609 0.428210
H -2.902325 -0.829100 -0.561758
H -2.799225 1.186643 0.192648
H -2.310228 1.316876 -1.220745
H -0.881835 1.559097 0.691273
H -1.121248 0.402640 1.651869
H 1.995078 -1.327348 -1.232045
H 2.960594 -0.136854 -1.148576
H 0.040927 -1.378085 0.016191
H -0.643430 -1.742092 -1.315277
H -0.441112 -1.856233 2.110521

26
oh.8h2o, E= -687.562558
O -0.216161 0.857224 -0.814522
O 1.210735 -0.533765 1.934462
O 2.932733 -1.569809 -0.428057
O 0.071418 -1.700653 -0.394665
O -3.301198 0.864313 -1.170151
O -2.698361 -1.651920 0.015296
O -1.596144 0.514367 1.469105
O 2.341432 0.880330 -1.589449
O 1.221010 2.144899 0.885500
H -0.053092 -0.745925 -0.692907
H 0.973111 -1.947346 -0.663381
H 2.739671 -1.346734 0.494431
H 2.833766 -0.712502 -0.901129
H 0.584740 1.796062 0.178328
H 0.663612 2.623660 1.506152
H 1.349103 0.852736 -1.480347
H 2.596634 1.551350 -0.944626
H -3.087810 1.264122 -0.318914
H -3.166542 -0.087932 -0.987701
H -1.096509 0.654443 0.613771
H -0.914221 0.175289 2.065894
H -1.825696 -1.975286 -0.272471
H -2.453530 -1.030031 0.728036
H 0.734324 -1.010622 1.223886
H 1.328985 0.375068 1.594866
H -0.930248 1.123763 -1.404054

26
oh.8h2o, E= -687.561377
O 0.050678 -1.405246 1.980593
O -0.403935 1.189910 1.908594
O 1.169692 2.206295 -0.031307
O -0.711950 1.420997 -1.851315
O -0.288864 -1.169925 -1.915108
O -1.982130 -1.571128 -0.074697
O -2.661255 0.903257 0.281190
O 1.701193 -1.760622 -0.360272
H 0.108812 -1.661619 2.904416
H -0.083697 -0.407973 1.973028
H 1.215475 -1.787594 0.480549
H 0.977133 -1.558997 -1.048099
H -0.369638 -1.642865 -2.746962
H -0.517356 0.437931 -1.975284
H -3.612450 0.977673 0.390797
H -2.460053 -0.084712 0.137171
H -1.336622 -1.485406 -0.887319
H -1.392910 -1.759525 0.674043
H -1.488499 1.426077 -1.270808
H 0.556947 1.979189 -0.776124
H -1.268928 1.234046 1.455638
H 0.228789 1.610437 1.263630
H 1.946229 1.620265 -0.140217
O 3.288600 0.345887 -0.069757
H 3.478889 0.290829 0.871731
H 2.721645 -0.463152 -0.249549

26
oh.8h2o, E= -687.560990
O -1.426038 2.682320 -0.286502
H 2.747561 1.750746 -1.447114
O 2.414349 1.063980 -0.865021
O 0.813641 1.936625 0.805258
O 1.219306 -1.324892 -1.415916
O 3.192503 -1.172852 0.704181
O -3.113893 0.457982 -0.343633
O -2.219281 -1.533470 1.454607
O -1.620698 -1.799398 -1.334023
O 0.451191 -0.608905 1.355265
H -1.164704 2.811144 -1.202958
H -0.568240 2.425000 0.188086
H 1.548028 -0.397032 -1.430863

H 1.903377 -1.761429 -0.882217
H 3.218062 -0.340848 0.197086
H 2.372937 -1.072275 1.223682
H 1.766926 1.521580 -0.143359
H 1.155181 2.484434 1.516866
H 0.534970 0.391402 1.274300
H 0.420430 -0.895428 0.428364
H -2.554982 1.262252 -0.277734
H -2.632882 -0.145915 -0.937741
H -1.324381 -1.197085 1.655263
H -2.676994 -0.764761 1.058040
H -0.656971 -1.699683 -1.426614
H -1.776963 -1.983230 -0.386804

26
oh.8h2o, E= -687.560555
O -2.337431 0.956136 0.531517
O -1.795872 -1.538337 1.067685
O -0.987821 -1.782580 -1.598860
O 1.467242 -2.193152 -0.560190
O 2.810829 0.125707 -0.154857
O 1.249359 2.120614 -1.004537
O -0.144282 1.690522 1.484104
O 1.005799 -0.557138 1.933542
O -1.301442 0.955305 -1.896925
H -2.131031 -0.597956 0.906863
H -3.154274 1.401707 0.766314
H 1.200871 -2.001112 0.351300
H 0.619949 -2.082666 -1.069753
H -1.112726 1.456352 1.141290
H 0.298486 2.084377 0.715104
H 2.447249 0.024694 0.744194
H 2.471372 -0.704453 -0.568039
H 1.096492 -0.644648 2.886605
H 0.530304 0.342597 1.776252
H -0.447914 1.378033 -1.714876
H -1.796646 1.014828 -1.024680
H 1.778004 2.784875 -1.453927
H 1.871057 1.381069 -0.760386
H -1.403588 -1.826483 -0.708943
H -0.915880 -1.396011 1.443329
H -1.082767 -0.831806 -1.842479

29
oh.9h2o, E= -764.028865
O 1.516045 -1.870039 1.054726
O 2.340311 0.447961 1.024526
O 0.400064 2.229191 1.449052
O -1.932578 0.802011 1.809133
O -1.196257 -1.890062 1.510785
O -1.811978 -1.607147 -1.275916
O -2.364110 0.913800 -0.860908
O -0.046748 2.332451 -1.431322
O 1.924877 0.664665 -1.554445
O 0.976746 -1.969761 -1.652427
H 1.926311 -0.862658 1.105635
H 2.101968 -2.456883 1.538725
H 1.155435 1.578118 1.385200
H 3.210447 0.631998 1.386966
H -1.087568 1.311822 1.817314
H 0.237267 2.479528 0.525332
H -1.668099 -0.142378 1.851320

H -1.484946 -1.946482 0.583237
H -0.214306 -1.901774 1.452659
H 1.225688 -2.070560 -0.706852
H 0.005564 -1.890380 -1.634244
H 2.198387 0.616079 -0.584107
H 1.621744 -0.247187 -1.755974
H 0.717513 1.667428 -1.533276
H 0.110509 3.029627 -2.072875
H -2.321456 0.941638 0.133012
H -1.608667 1.465366 -1.151528
H -2.030254 -0.626419 -1.173104
H -2.546514 -2.001437 -1.753066

29
oh.9h2o, E= -764.027903
O -0.247948 2.111772 -1.708269
O -2.136635 0.091850 -1.716979
O -0.839448 -2.305706 -1.194120
O 2.153417 1.001591 -1.208576
O 1.958724 -1.618294 -1.170527
O -1.151168 -1.741475 1.410999
O -2.398815 0.385077 1.067911
O -0.611955 2.489799 1.047665
O 1.844679 1.158997 1.582647
O 1.391668 -1.390061 1.613064
H -0.909703 1.393604 -1.846528
H -2.390884 0.241452 -0.784707
H -1.690670 -0.785359 -1.683492
H 0.225513 2.078321 1.329400
H -0.418370 2.404885 -0.786522
H 1.295997 1.415400 -1.502465
H 2.195712 1.199780 -0.253606
H -0.998224 -2.193307 -0.215716
H 0.112482 -2.140201 -1.298775
H 1.652314 0.161763 1.648527
H 2.433394 1.373891 2.310516
H 2.642547 -1.980672 -1.739382
H 2.023919 -0.617005 -1.249333
H -1.903706 -0.534453 1.274837
H -1.541516 -2.427813 1.958008
H -1.283199 1.777880 1.138486
H -3.217042 0.390827 1.570382
H 1.688613 -1.673132 0.730504
H 0.382670 -1.554267 1.609337

29
oh.9h2o, E= -764.027468
O 2.617638 -1.501848 0.750344
O 2.690577 1.195069 -0.075582
O -0.081332 -1.846660 1.665119
O -1.247859 0.627583 1.996776
O 0.455972 2.409819 1.159571
O -2.865180 0.750028 -0.217505
O 0.357510 -1.960810 -0.990418
O 1.032029 0.192538 -2.098661
O -2.288338 -1.949132 -0.666661
O -0.775531 2.177827 -1.395627
H -2.221127 1.274314 -0.742352
H -1.933154 0.669287 1.285086
H -0.853526 -0.274609 1.929547
H -2.747501 -0.178283 -0.510525
H -1.316443 -1.946942 -0.909196

H -0.176939 1.480915 -1.739261
H -0.352300 2.453084 -0.562489
H 0.855836 -1.731066 1.894953
H 0.000706 -1.963363 0.682392
H 2.220763 0.910277 -0.884837
H 2.005339 1.663748 0.436258
H 1.964195 -1.716611 0.054989
H 2.788588 -0.550121 0.599282
H 0.752840 -0.731793 -1.690555
H 0.455117 3.140705 1.783007
H -0.169602 1.709125 1.537132
H 0.597526 -2.787786 -1.417880
H 1.244840 0.042968 -3.023214
H -2.279042 -2.219158 0.258828

29
oh.9h2o, E= -764.026398
O -0.145905 -0.406222 -1.509080
O -1.399899 -2.739247 -0.588419
O -2.567479 0.546402 -0.629734
O -2.260376 -1.141669 1.569952
O 0.448976 -0.259695 1.968081
O 1.334339 -2.007127 -0.018557
O 3.124222 -0.171097 -1.275953
O 0.477886 1.711145 -0.074641
O 2.898641 1.343728 1.087602
O -1.945983 3.096235 -0.286675
H -1.124191 -1.914814 -1.052368
H -0.547971 -3.093135 -0.298279
H 0.810397 -1.436815 -0.657701
H 2.247025 -1.790913 -0.262964
H 0.250529 0.975783 -0.720166
H -0.264464 2.346445 -0.103405
H 2.204918 -0.011569 -1.536459
H 3.223909 0.363254 -0.460763
H -1.736036 0.198292 -1.039359
H -2.630342 0.042905 0.211404
H 0.808877 -0.962017 1.383338
H 0.327481 0.499320 1.362725
H -2.052185 -1.848519 0.925400
H -1.384956 -0.843440 1.886238
H -2.089984 3.491183 -1.151593
H -2.295124 2.161555 -0.371659
H 2.092715 1.715811 0.665650
H 2.516477 0.746854 1.746679
H -0.072450 -0.419790 -2.467324

29
oh.9h2o, E= -764.025546
O -1.787449 -1.404894 1.590718
O -1.822158 1.223284 1.456436
O 0.712150 2.191607 1.441898
O 0.905593 -2.170958 1.019626
O 2.378216 -0.071126 1.409141
O 0.595521 2.353947 -1.305732
O -2.016563 1.208506 -1.325148
O -1.797466 -1.359275 -1.302038
O 0.665664 -1.929998 -1.525344
O 2.249387 0.184814 -1.491623
H -1.783972 -0.398831 1.590847
H -2.363410 -1.672900 2.311167
H -2.026199 1.364159 0.510228
H -0.907669 1.595536 1.562786
H -0.807919 -1.621053 -1.474747
H -1.947060 -1.585830 -0.369582
H -0.004073 -2.000556 1.321213
H 0.842184 -2.174908 -0.005560
H 0.726261 2.384702 0.472943
H 1.326027 1.437861 1.539110
H 1.664524 -0.625953 -1.604085
H 0.906778 -2.669097 -2.089240
H 3.103814 -0.314404 1.989437
H 1.800574 -0.900520 1.307520
H -0.298664 1.985355 -1.424087
H -1.923574 0.192118 -1.373735
H -2.691279 1.454119 -1.963096
H 2.512242 0.145547 -0.557996
H 1.208254 1.597406 -1.486583

29
oh.9h2o, E= -764.025304
O -0.137579 0.040712 -1.589312
O 1.649489 1.536825 -0.400852
O 3.501087 -0.170165 0.630170
O -1.517029 1.200848 0.288642
O -3.784027 -0.280835 0.515638
O 1.860647 -1.820223 -1.124916
O -1.854506 -1.979168 -0.869460
O 0.516021 0.131564 1.852244
O 0.173363 -2.442522 0.976648
H -1.070871 0.767708 -0.517367
H -0.965835 0.863798 1.030999
H 1.012282 0.562045 1.131664
H 0.398447 -0.814717 1.581023
H -0.655728 -2.411113 0.451606
H 0.859713 -2.428709 0.272615
H -3.037949 0.365378 0.448288
H -3.650574 -0.706354 1.368731
H 1.012311 0.993535 -0.965679
H 1.203733 2.390441 -0.260092
H -1.246826 -1.281909 -1.223348
H -2.627505 -1.492794 -0.537334
H 1.176940 -1.159382 -1.394399
H 2.550219 -1.313062 -0.662179
H 3.018019 0.596380 0.254092
H 2.993535 -0.381626 1.423636
H -0.866675 2.816479 0.085023
O -0.315726 3.607116 -0.143040
H -0.551784 3.805136 -1.054590
H -0.285358 0.235551 -2.518797

29
oh.9h2o, E= -764.024443
O -0.455856 -2.660241 0.591589
O 0.562950 -1.091952 -1.328005
H 1.054006 -1.526814 -2.031538
O -2.095872 -1.254098 -1.695880
O -3.150234 0.790603 -0.096770
O -1.502607 -0.349747 1.830734
H -1.254292 -1.208584 1.433212
H -2.192446 0.026236 1.239989
H -2.563182 1.569337 -0.166967
H -2.842616 0.162916 -0.783732
H -1.107265 -1.135017 -1.669875

H -2.241411 -1.989694 -1.089537
H -0.084709 -2.158735 -0.182520
H 0.317731 -2.739084 1.165447
H 0.775944 0.336410 -1.314341
O 0.940198 1.372722 -1.183081
H 1.164435 1.743718 -2.041185
O 2.213953 -1.248773 0.942979
H 1.851508 -0.506893 1.463727
H 1.699139 -1.205834 0.105775
O 3.768059 0.655545 -0.588488
H 2.896567 1.051293 -0.743698
H 3.550040 -0.085281 0.002888
O 0.787003 1.108138 1.742653
H 0.955420 1.317046 0.812555
H -0.066976 0.604862 1.766178
O -1.145294 2.773351 -0.008936
H -0.843630 2.665587 0.902763
H -0.446652 2.314156 -0.523493

29
oh.9h2o, E= -764.024150
O -2.785211 -0.609105 -1.282161
O -3.254865 1.760546 0.307783
O -0.964204 0.583905 1.086360
O -0.027280 0.235715 -1.413275
O 2.796936 0.007131 -1.619012
O 2.499528 -1.279571 0.949437
O 0.463951 -2.195860 -0.408147
O -1.946847 -2.042580 0.994352
O 1.551133 1.176986 2.013791
O 1.578951 2.322386 -0.523307
H -1.833221 -0.396711 -1.384590
H -3.142754 0.172310 -0.822183
H -2.921150 2.495234 -0.216819
H -2.452711 1.422735 0.776543
H -0.642585 0.497819 0.149764
H -0.161782 0.832122 1.600371
H 1.815808 -0.024354 -1.679122
H 2.973729 -0.465905 -0.789574
H 1.698253 -1.692773 0.493601
H 2.967765 -2.000200 1.379036
H -0.353019 -2.338100 0.116399
H 0.262293 -1.379712 -0.940022
H 1.668033 1.723723 1.205296
H 1.950799 0.318757 1.776002
H -1.625503 -1.175086 1.311381
H -2.448005 -1.774487 0.198190
H 0.863027 1.709527 -0.821154
H 2.350325 1.867501 -0.902885
H -0.266040 0.531168 -2.296800

29
oh.9h2o, E= -764.023469
O -0.301493 -1.459751 -1.123917
O -2.813474 -1.402823 -0.697077
O -2.654765 -0.470279 1.740536
O 0.156308 -0.641950 1.286348
O -2.355100 1.986397 0.351325
O 0.519966 1.869700 0.216914
O -0.975972 1.006168 -2.026146
O 3.303294 1.393771 0.070168
O 2.507697 -0.989425 -1.336472
O 2.925003 -1.106035 1.468945
H -0.705281 0.071109 -1.852723
H -1.654014 1.219699 -1.361801
H -1.802064 -1.501718 -0.901366
H -3.106119 -0.703881 -1.291944
H 0.023652 -1.086224 0.384845
H -0.719226 -0.656502 1.706450
H 1.528445 -0.989494 -1.318637
H 2.782950 -0.073345 -1.145974
H 3.327112 0.694723 0.750437
H 2.402966 1.764796 0.146768
H 0.236491 1.685176 -0.702344
H 0.424219 0.997380 0.668942
H -1.419816 2.212442 0.504535
H -2.536453 1.261098 0.974215
H -2.795223 -0.877988 0.830610
H -3.306736 -0.860036 2.328545
H 2.900763 -1.349452 0.522521
H 1.978320 -0.995542 1.674124
H -0.051706 -2.298420 -1.522198

29
oh.9h2o, E= -764.023179
O -0.269874 2.146877 1.313134
O -2.456970 0.663448 1.162837
O -1.444296 -2.043129 1.259996
O 1.319399 -1.627168 1.614124
O 1.865214 0.854575 1.511624
O 2.414548 0.699771 -1.335813
O 1.558903 -1.816949 -1.129512
O -1.153065 -1.779316 -1.578499
O -2.069316 0.610155 -1.514443
O 0.011363 2.347791 -1.289603
H -1.648765 1.242155 1.315740
H -0.331616 2.952753 1.832495
H -2.170526 -0.245408 1.369195
H -0.506770 -1.914476 1.500905
H -1.431261 -2.080522 0.286905
H 1.828661 -2.021997 2.326350
H 1.525997 -0.613399 1.623338
H 2.249035 0.981799 0.629503
H 0.973757 1.425203 1.499195
H -0.102174 2.382161 -0.285403
H 0.843880 1.865532 -1.416705
H -2.358140 0.673929 -0.563552
H -1.329576 1.269174 -1.569713
H -1.505630 -0.814283 -1.601694
H -1.646404 -2.269612 -2.240983
H 1.548815 -1.912670 -0.153201
H 0.611848 -1.848978 -1.388028
H 2.095124 -0.246017 -1.317884
H 3.146498 0.726206 -1.957222

29
oh.9h2o, E= -764.022728
O -2.528415 -0.628149 -1.270289
O -3.219041 1.958161 -0.141894
O -0.667037 1.531306 0.563750
O 0.120709 0.077107 -1.445659
O 2.562929 1.010324 -0.751144
O 3.548953 -1.576839 -0.108195
O 0.899272 -1.968756 0.034866

O -1.982036 -2.684298 0.477310
O -0.568899 -0.844676 2.122160
O 1.734209 2.945916 0.875076
H -1.571416 -0.386295 -1.363107
H -2.936382 0.144997 -0.842487
H -3.106489 2.442404 -0.965690
H -2.304361 1.912755 0.236351
H -0.359877 1.058300 -0.271754
H 0.070666 2.123184 0.814439
H 1.677667 0.653506 -1.040648
H 3.056051 0.224464 -0.447671
H 2.588960 -1.813360 0.018819
H 3.776920 -1.961778 -0.959816
H 0.572885 -1.598143 0.885129
H 0.606905 -1.286379 -0.637218
H 1.776240 3.703538 0.284118
H 2.152364 2.205516 0.351999
H -0.675601 0.028820 1.694734
H -1.281833 -1.405150 1.759167
H -1.065811 -2.824977 0.191375
H -2.318393 -2.014716 -0.161958
H 0.136350 0.232553 -2.393627

29
oh.9h2o, E= -764.022492
O 2.476621 0.289707 -1.616522
O 3.532884 -0.858578 0.733098
O 1.050517 -1.953083 0.617768
O -0.109840 -0.571237 -1.436926
O -2.919362 0.508709 -1.585793
O -2.859648 1.707470 1.008739
O -0.236522 1.352072 0.365846
O -1.271752 -0.728926 1.997350
O 2.480002 2.054739 0.348732
O -2.223599 -1.941561 -0.311377
H 1.537641 -0.042971 -1.586079
H 2.983375 -0.329721 -1.056420
H 3.371694 0.039979 1.049470
H 2.658551 -1.312724 0.816987
H 0.667116 -1.568086 -0.207706
H 0.393532 -1.746302 1.302381
H -1.959636 0.343462 -1.623186
H -3.044527 1.001464 -0.751903
H -1.910065 1.847948 0.809865
H -2.826472 0.936313 1.592881
H 0.659309 1.715614 0.492494
H -0.186546 0.744020 -0.422096
H -1.396378 -1.631028 -0.754537
H -2.870588 -1.324524 -0.692130
H 2.521071 1.500336 -0.484509
H 2.780423 2.934706 0.105488
H -0.820369 0.009612 1.544198
H -1.715483 -1.222770 1.271347
H -0.197052 -0.769832 -2.373873

29
oh.9h2o, E= -764.022314
O -2.135551 -1.538671 -0.972773
O 0.288868 -1.994229 -0.851392
O 1.179022 -0.929444 1.937814
O 2.862458 -1.189101 -0.118848
O 0.388616 0.731898 -1.475137
O 2.949874 1.488727 -0.875077
O 0.747943 1.758807 1.056108
O -2.189794 1.798366 1.189460
O -1.856605 -0.927023 1.575867
O -2.263117 1.037111 -1.578714
H -0.766032 -1.898258 -1.019979
H 0.324928 -2.114893 0.104910
H -0.567533 0.949338 -1.663004
H 0.389417 -0.236045 -1.302480
H -2.358329 0.051437 -1.428647
H -2.387685 1.424450 -0.689713
H -2.044577 -1.220629 0.629895
H -0.897304 -0.988275 1.681771
H -2.176668 0.841445 1.423650
H -1.253360 2.048875 1.251203
H 0.851624 0.945598 1.579256
H 0.526304 1.419250 0.156660
H 1.535515 -1.286293 2.755962
H 1.888253 -1.033937 1.242174
H 3.043290 -0.272784 -0.428362
H 2.162295 -1.530376 -0.708321
H 2.122930 1.360477 -1.385191
H 2.599653 1.855610 -0.047278
H -2.766430 -2.206509 -1.250980

29
oh.9h2o, E= -764.021692
O -0.186752 0.175018 -1.291555
O 0.821022 -2.306503 -0.732019
O 2.199976 -1.283369 1.608585
O 3.130200 -0.009187 -0.445398
O 1.607375 1.932756 -0.941881
O -0.226620 0.052195 1.541766
O -0.305006 2.819034 0.747892
O -2.446568 1.579595 -0.588612
O -2.000002 -2.307709 -1.017072
O -2.898134 -0.645694 1.140281
H 0.528964 -1.399534 -0.975930
H 1.229089 -2.209631 0.143211
H 0.525354 0.919311 -1.289595
H 1.884072 2.517497 -1.652084
H -2.768954 0.924809 0.063242
H -1.824481 2.162538 -0.097562
H -1.814007 -1.527355 -1.555810
H -1.099058 -2.606344 -0.785623
H -2.725506 -1.329262 0.464774
H -2.016595 -0.492807 1.531134
H 0.590266 -0.405018 1.815280
H -0.173829 0.067273 0.561033
H 2.883029 -1.513250 2.243024
H 2.637901 -0.730447 0.879833
H 2.569179 0.858533 -0.640669
H 2.860317 -0.630118 -1.132780
H -0.241267 2.074461 1.366869
H 0.443422 2.629983 0.117044
H -1.051829 0.640278 -1.231284

29
oh.9h2o, E= -764.021605
O -1.144918 -1.547646 -0.398315
O -2.272671 -0.137991 1.417827
O 1.230977 -2.362312 -1.405623

O 1.885547 0.287436 -1.528582
O -0.225079 1.677465 1.244748
O 2.345158 2.732433 0.097366
O 2.076807 0.050830 1.207014
O -0.804230 0.870414 -1.433696
O 0.658858 -2.478869 1.503023
O -3.698657 1.068926 -0.813581
H -0.074392 -2.212131 0.903469
H 1.170018 -1.666479 1.635772
H 0.303164 -2.084229 -1.173737
H 1.529730 -2.705677 -0.550422
H -0.983116 -0.071582 -1.135329
H -0.667881 1.346345 -0.592417
H -1.834530 -0.757011 0.718337
H -2.374305 -0.665264 2.215046
H 2.367658 2.078146 -0.618347
H 1.418521 2.743214 0.379332
H 0.481804 1.015699 1.372733
H -1.047237 1.184271 1.458706
H 2.601541 0.838900 1.402184
H 2.030670 0.062046 0.220022
H 1.846520 -0.690423 -1.653149
H 0.951230 0.565977 -1.675237
H -2.844331 1.128449 -1.270556
H -3.455535 0.688065 0.044947
H -1.833866 -2.083794 -0.802811

32
oh.10h2o, E= -840.490439
O -2.757824 1.409235 -0.633138
O -2.840321 0.165862 1.668534
O -1.991064 -0.820202 -1.942942
O -2.104334 -2.341962 0.447887
O 0.609024 -1.634543 0.446803
O 3.245778 -2.170127 0.674655
O 0.691991 -0.242394 -1.753561
O -0.062388 2.041189 -0.753476
O 3.104652 0.396895 -0.632359
O 2.229418 2.531937 0.810507
O -0.097787 0.737431 1.701924
H -2.597475 0.626577 -1.225724
H -2.125554 -1.479266 -1.232442
H -2.438737 -1.595975 0.975551
H -2.878120 0.684737 0.802682
H 0.254604 1.232944 -1.267971
H 0.648305 -1.226343 -0.475549
H 0.448026 -0.851834 1.020418
H -0.183414 1.210234 0.842257
H 2.668886 1.781945 0.337163
H 3.330086 -0.401077 -0.121759
H 1.728030 2.100657 1.515247
H -1.901470 1.881042 -0.666792
H -1.128827 -2.265306 0.511618
H 0.955199 -0.448079 -2.654347
H -1.018766 0.568273 1.970852
H 0.737736 2.487408 -0.415451
H 2.258794 -2.065389 0.690713
H 3.408941 -2.776075 -0.054588
H 2.250900 0.167612 -1.084790
H -1.017142 -0.633254 -1.921922
H -3.617160 0.414595 2.176161

32
oh.10h2o, E= -840.490063
O 3.019833 0.657174 -0.014806
O 1.890458 -0.803910 2.076836
O 1.492357 -2.515661 -0.108883
O 1.137939 -0.511670 -1.635411
O -2.804481 1.152095 -1.216044
O -3.308016 1.134737 1.621115
O -0.151997 1.505882 -0.579987
O -0.832707 -0.024203 1.624749
O -1.226604 -2.477748 0.506408
O 2.080437 3.142522 -0.102792
O -1.551663 -1.292675 -1.939434
H 2.417950 0.219870 -0.669537
H 2.757162 0.240826 0.835132
H 0.978367 -0.460694 2.143018
H 1.809531 -1.540064 1.438477
H 1.385594 -1.753291 -0.798512
H 2.140321 -3.130787 -0.460621
H -1.895316 1.480983 -1.061384
H -3.169441 1.070688 -0.315606
H -3.094621 2.051950 1.819312
H -2.453583 0.654912 1.756529
H 0.494176 2.214635 -0.392543
H 0.355442 0.794746 -1.077540
H -0.615240 -0.992014 -1.904057
H -2.090747 -0.491040 -1.779093
H 2.527560 2.252972 -0.004506
H 2.424918 3.500893 -0.926112
H -0.550220 0.517703 0.851472
H -1.016856 -0.931583 1.266585
H -0.297871 -2.737909 0.360470
H -1.508451 -2.167933 -0.387032
H 1.436875 -0.527210 -2.548468

32
oh.10h2o, E= -840.488586
O -0.106148 0.060477 -1.245077
O 3.338226 0.072945 0.143958
O -1.736449 1.819042 0.091954
O 0.675059 2.749624 1.210164
O 1.687817 1.864437 -1.234652
O 1.852198 -1.897411 -1.292976
O 0.744350 -0.213541 1.413193
O -3.167311 0.107534 -1.929866
O 0.595138 -2.899517 0.950607
O -1.859172 -1.779305 -0.076955
O -2.075874 -0.045846 2.114607
H 2.635783 -0.010319 0.812420
H 2.998001 0.788955 -0.424824
H 1.131083 -1.238531 -1.427477
H 2.562967 -1.361724 -0.892143
H -3.039870 0.868336 -1.346515
H -2.973238 -0.658743 -1.362969
H 0.388066 -0.076238 0.512063
H 0.774747 -1.200857 1.472465
H -1.141753 1.207408 -0.411645
H -1.097351 2.414958 0.528046
H 0.952027 1.147573 -1.348470
H 1.754764 2.341728 -2.065506
H 0.804727 1.906896 1.669101
H 1.122059 2.610088 0.350261

H -1.169816 -1.203812 -0.479788
H -2.092048 -1.308898 0.751626
H -1.113857 -0.101910 2.241006
H -2.152133 0.703715 1.484920
H 1.111233 -2.748297 0.124207
H -0.331828 -2.840331 0.655288
H -0.706232 0.052473 -2.001717

32
oh.10h2o, E= -840.486404
O -2.646397 0.051843 1.853407
O 0.064295 0.032673 1.646093
O 0.233646 2.472258 0.550725
O -2.472418 1.745234 -0.329558
O -1.537204 -0.390799 -1.709362
O 0.077987 -1.878279 -0.349863
O -2.885482 -1.945940 -0.111374
O 0.601770 1.340348 -2.006478
O 3.085941 0.548045 -1.061795
O 2.876778 -2.118672 -0.043001
O 2.899165 0.229705 1.716790
H -2.707126 0.762912 1.174143
H -2.819463 -0.752517 1.325074
H 2.978032 -1.560174 0.748405
H 3.125282 -0.424828 -1.027214
H 2.233089 0.763215 -1.500778
H 1.913378 -2.151436 -0.192645
H -0.437185 -1.313438 -1.017220
H 1.949746 0.202937 1.920183
H 2.957212 0.552284 0.791963
H 0.465112 1.894380 -1.216615
H -0.137617 0.689843 -1.956678
H 0.210497 1.573297 0.976408
H 1.061772 2.873027 0.831745
H -3.785227 -2.096037 -0.413952
H -2.455913 -1.326534 -0.793601
H -2.157169 1.020682 -0.932192
H -1.688269 2.280929 -0.143675
H -0.887614 0.024018 1.918492
H 0.123294 -0.656584 0.946207
H -1.743682 -0.534779 -2.637180
H -0.582784 -2.512520 -0.045534

32
oh.10h2o, E= -840.486213
O -1.187490 -2.202463 -0.356926
O -3.683276 -0.890787 -0.980049
O -2.080314 1.017730 0.103561
O 0.000164 0.063913 -1.210664
O 1.144503 2.465865 -0.635799
O 3.317267 1.053509 0.456110
O 2.602969 -0.944085 -1.390584
O 1.527111 -2.983787 0.330791
O 1.106274 -0.415401 1.408739
O -1.510596 -0.849743 2.122469
O -1.023119 3.572956 0.444972
H -0.712205 -1.403900 -0.718307
H -2.112889 -2.056218 -0.619421
H -3.675371 -0.645652 -1.910246
H -3.268462 -0.120292 -0.525864
H -1.282895 0.706632 -0.424154
H -1.913148 1.965646 0.283893
H 1.693597 -0.571121 -1.422925
H 2.459938 -1.788878 -0.924939
H 1.470525 -2.194940 0.906349
H 0.614158 -3.081046 0.016593
H 0.260765 -0.489973 1.904354
H 0.820505 -0.166381 0.512140
H -0.136698 3.211453 0.164453
H -1.233472 4.226014 -0.228917
H -1.837858 -0.081560 1.612707
H -1.460719 -1.537480 1.429515
H 0.741619 1.607077 -0.921648
H 1.987903 2.189368 -0.220102
H 3.279858 0.361266 -0.239627
H 2.748974 0.671026 1.145167
H -0.152075 0.097295 -2.159993

32
oh.10h2o, E= -840.486191
O 2.201144 -1.362661 -1.474678
O 2.338458 1.223677 -1.309027
O -0.237453 2.132691 -1.204187
O -0.488371 -2.153693 -1.334559
O -1.937748 0.124280 -1.980980
O -3.820673 0.045016 -0.098307
O -1.841724 0.183914 1.915820
O -0.575880 -2.000482 1.461255
O 2.200678 -1.565532 1.366867
O 2.395733 1.276714 1.402441
O -0.174936 2.069241 1.252343
H 2.249612 -0.348358 -1.470823
H 2.853817 -1.666796 -2.110560
H 2.324491 -1.615875 0.401969
H 1.244258 -1.725577 1.489639
H -3.157012 0.121201 -0.843611
H -4.056462 -0.887757 -0.091585
H 1.447806 1.579451 1.454060
H -0.338755 2.896429 1.714016
H -1.419629 -0.697727 -1.827651
H -1.328682 0.862001 -1.743468
H 2.505186 1.325333 -0.334498
H 1.432454 1.597703 -1.416323
H -0.468095 3.007136 -1.528598
H -0.245114 2.168640 -0.106880
H -0.575506 -2.197530 -0.361927
H 0.450882 -1.929814 -1.483225
H -1.076118 -1.128593 1.660239
H -0.891776 -2.649212 2.095576
H 2.367325 0.311122 1.553396
H -2.580863 0.243680 1.277448
H -1.211647 0.949213 1.696889

32
oh.10h2o, E= -840.485318
O -1.769751 -0.665370 -1.456059
O -2.495578 1.788437 -1.076905
O -2.027992 1.796037 1.500870
O -2.342637 -1.099420 1.117666
O 0.694649 1.730843 0.914899
O -0.091513 -2.600410 1.456382
O 1.692994 -0.725862 1.533680
O 0.205203 -2.550195 -1.264761
O 0.335225 1.021167 -1.778864

O 3.548410 1.845616 0.193603
O 2.475803 -0.627577 -1.180545
H -0.396211 0.352027 -1.767470
H 0.397637 1.350950 -0.858375
H -2.332723 0.793492 -1.251230
H -1.735976 2.189034 -1.520158
H -2.215426 -1.005900 0.130475
H -2.274618 -0.191446 1.451510
H -0.501764 -1.860334 -1.371616
H 1.043143 -2.081062 -1.413025
H 2.255211 -0.706695 -0.232265
H 1.790499 -0.021279 -1.542559
H 2.644951 2.091822 0.441504
H 3.426014 1.083725 -0.394222
H 0.992925 0.860199 1.256963
H -0.196791 1.878324 1.283028
H -2.266170 1.863449 0.524743
H -2.622380 2.384885 1.973226
H 1.022733 -1.482975 1.609127
H 2.381649 -0.848461 2.191851
H -0.951585 -2.111505 1.479759
H 0.027924 -2.784013 0.492694
H -2.287545 -1.060356 -2.163695

32
oh.10h2o, E= -840.483002
O 2.290731 0.763937 -1.319277
O 2.445738 0.899043 1.117645
O 0.093441 2.031927 -1.662801
O 1.837890 -1.619602 1.641036
O -2.004369 0.193781 -2.149841
O -0.861955 -2.133865 -1.278543
O 1.925012 -1.929926 -1.242433
O -3.502005 -0.113792 0.115383
O -1.881843 0.889362 2.168118
O -1.011377 -1.647170 1.496160
O 0.128069 2.499349 1.110015
H 0.987519 1.532582 -1.609784
H 3.060948 0.994817 -1.845314
H -3.117041 -0.961452 0.371270
H -3.049221 0.093164 -0.740123
H -1.566447 -0.675323 -1.983906
H 2.015639 -2.061678 -0.285779
H 2.064426 -0.955746 -1.359907
H 2.095817 -0.660208 1.529744
H 2.233644 -1.919125 2.463604
H 0.193876 2.710343 -2.335627
H -1.301644 0.853341 -1.994764
H 0.113432 -2.123282 -1.389666
H 0.001785 2.467750 0.141789
H 0.941790 1.974816 1.246890
H 3.257797 1.314606 1.418783
H 2.459015 0.874392 0.013715
H -0.045064 -1.693394 1.610004
H -0.991338 -1.993123 -0.317611
H -1.263781 -0.749686 1.817824
H -1.210836 1.517241 1.820634
H -2.554978 0.795602 1.464525

32
oh.10h2o, E= -840.480735
O -1.191193 -1.571295 1.992296
O 1.408250 -1.851240 1.708854
O 2.695274 0.289519 0.294692
O 1.688715 2.564119 -0.935385
O -0.343390 1.305208 -2.307724
O -2.691715 1.127169 -0.624623
O -2.253174 -1.438995 -0.658289
O -0.166187 -1.391852 -2.207676
O -1.699090 1.213747 2.001121
O 1.794213 -2.067592 -0.911566
O 0.910335 2.171770 1.787165
H -0.194895 -1.665602 1.905009
H -1.456844 -2.172309 2.693438
H 2.456574 -0.463689 -0.283539
H 2.396461 -2.619736 -1.415044
H 0.926085 -1.802431 -1.543224
H -2.587812 0.120770 -0.661766
H -1.916370 -1.640158 0.232728
H -2.092756 1.326752 1.114305
H -3.593323 1.319212 -0.895554
H 1.860234 -0.991215 1.702656
H 1.532288 -2.136377 0.766032
H -1.455013 -1.503076 -1.281711
H -0.277997 -1.810113 -3.065690
H 1.412131 1.382872 1.530877
H -0.018336 1.872403 1.897721
H -1.581553 0.250061 2.091856
H -1.160138 1.423840 -1.795660
H -0.214847 0.319332 -2.327406
H 0.971529 2.151197 -1.473181
H 1.282802 2.739127 -0.066723
H 2.495466 1.094669 -0.236030

32
oh.10h2o, E= -840.480518
O -0.713763 -1.520515 -1.977625
O 1.415718 -0.031083 -2.092063
O 1.421128 2.349364 -0.917422
O -2.914199 -0.971426 -0.225452
O -3.088048 1.749039 -0.422515
O -0.814127 2.498751 0.474297
O 0.167301 0.463669 1.630154
O -1.546294 -1.707811 2.050166
O 0.402778 -2.556469 0.352197
O 2.756475 -1.422854 -0.005808
O 2.841475 1.161703 1.300240
H 0.059416 -0.875967 -2.064894
H -0.840546 -1.919936 -2.842010
H -0.061667 -2.379026 -0.488926
H -0.266498 -2.370850 1.061034
H -2.193154 2.082060 -0.034572
H -0.861142 3.291534 1.016163
H 3.037950 0.301788 0.893476
H 1.936638 1.014734 1.651423
H -3.051283 0.007623 -0.244548
H 2.043809 -0.488982 -1.505284
H 1.371185 0.905457 -1.734891
H 0.542126 2.491619 -0.446061
H 2.041729 2.135717 -0.195064
H -2.170402 -1.472864 1.322340
H -2.218928 -1.129180 -0.886810
H -1.083499 -0.865021 2.225493
H 3.410914 -2.125851 -0.034007

H 1.889964 -1.860999 0.237482
H -0.276045 1.312654 1.226647
H 0.298689 -0.119246 0.871736
H -3.036795 1.965790 -1.358078

32
oh.10h2o, E= -840.480441
O -1.904872 -1.962407 -0.882146
O -2.686177 0.403277 -1.722746
O 0.078878 1.202629 -1.871971
O -0.231834 1.946930 0.861049
O 0.635842 -0.385203 1.460875
O 3.209632 0.013501 1.063635
O 3.380168 -2.038125 -0.649096
O 0.659827 -1.235391 -0.887062
O -2.392980 -1.376729 1.816425
O -2.825542 1.156429 1.026220
O 2.344961 2.439993 -0.231235
H -2.071814 -1.923755 0.082173
H -2.531523 -0.414391 1.594655
H -2.945817 1.030070 0.067353
H -2.421319 -0.530712 -1.449454
H 0.642922 -0.924299 0.128532
H 0.108701 1.033750 1.195538
H -0.224154 1.821140 -0.108459
H 0.372393 0.290478 -1.610793
H 3.413174 -1.237465 -0.051052
H 3.107051 0.865629 0.599759
H 1.882439 2.157638 -1.034064
H -0.919862 -1.873517 -0.965226
H -1.931542 1.580984 1.089848
H 0.051714 -0.881473 2.042640
H -0.869779 1.093721 -2.053157
H 1.543116 -1.623932 -1.023802
H 1.606721 2.515220 0.402832
H 3.317728 -2.777217 -0.036133
H 2.284974 -0.176900 1.394251
H -3.198844 -1.649323 2.264371
H -3.359513 0.305114 -2.401398

32
oh.10h2o, E= -840.480180
O -0.210821 -0.326247 1.468606
O -1.925903 0.039400 -1.919656
O 1.327684 1.252317 -0.036562
O -1.080806 2.502631 -0.840974
O -2.071875 1.483358 1.344111
O -3.807489 -0.283492 -0.090583
O -2.248429 -2.272737 1.299091
O -0.211460 -1.748269 -0.707462
O 3.975596 1.406621 1.068575
O 4.365914 -1.305714 0.004596
O 2.256375 -0.790313 -1.635817
H -1.569384 0.931586 -1.749579
H -1.236063 -0.591828 -1.603317
H -1.563653 -1.606226 1.530904
H -2.946391 -1.736620 0.889045
H 3.073798 1.510757 0.717949
H 4.297050 0.593798 0.645009
H -0.078591 -1.217991 0.143920
H -0.852117 -2.412507 -0.411990
H 0.789988 0.689100 0.594949
H 0.680424 1.873164 -0.411287
H -1.347011 0.797323 1.525290
H -2.837022 0.958292 1.055517
H -1.321396 3.423093 -0.973354
H -1.498479 2.204982 0.031518
H 3.895739 -1.576953 0.799585
H 3.648322 -1.191331 -0.667252
H 1.496263 -1.383014 -1.502442
H 1.962169 0.035262 -1.188517
H -4.682017 -0.276270 -0.487921
H -3.148954 -0.171951 -0.838470
H 0.287034 -0.513107 2.269031

32
oh.10h2o, E= -840.479831
O -0.387334 0.841482 0.834638
O 1.878671 0.595570 1.828118
O 0.179054 0.242723 -1.637685
O 3.310038 -1.402494 0.593434
O 1.199432 -2.425697 -0.913772
O -1.033389 -1.907141 0.647713
O -2.309671 -1.210658 -1.729341
O 2.807036 1.224540 -0.692410
O 0.381067 3.003830 -0.748345
O -3.230634 1.040953 -0.406261
O -2.967215 -0.134098 2.139537
H 2.066337 0.756871 -1.115172
H 2.584522 1.173498 0.261648
H 0.039958 2.396713 -0.052593
H 1.330157 2.814096 -0.763834
H -2.576136 -0.984608 1.898508
H -3.262684 0.246491 1.287178
H -0.134778 0.384386 -0.690929
H 0.136613 1.143933 -1.991294
H -0.718732 -0.986988 0.787757
H -0.264231 -2.340287 0.220627
H 0.874618 0.697681 1.497925
H 1.956761 1.044446 2.673356
H 0.980816 -1.625506 -1.415327
H 1.986790 -2.178141 -0.382220
H -2.348658 1.417249 -0.277065
H -3.057228 0.254226 -0.970869
H -1.492072 -0.789171 -2.043705
H -2.008429 -1.625745 -0.891659
H 3.634434 -0.731015 -0.024104
H 2.801491 -0.857413 1.231337
H -1.145986 0.837204 1.435425

32
oh.10h2o, E= -840.479643
O -2.141832 -1.014756 -1.850178
O -2.942821 1.240187 -0.805262
O -0.263319 2.041583 -0.748257
O 0.028603 1.286956 1.893928
O 0.541931 -1.475505 0.696027
O 2.971679 -1.938218 0.641598
O 3.336381 0.009392 -1.036042
O 0.538746 -0.374337 -1.581656
O -1.998695 -2.281109 0.530176
O -2.649117 0.193579 1.849015
O 2.411789 2.219688 0.478616
H -2.152431 -1.597740 -1.041445

H -2.354901 -1.570776 1.096839
H -2.819623 0.651352 1.005849
H -2.680805 0.373911 -1.259532
H 0.528852 -0.856481 -0.648964
H 0.387051 -0.723712 1.287439
H -0.171038 1.584166 0.977480
H 0.033560 1.174908 -1.138695
H 3.270186 -0.758234 -0.375791
H 3.311733 -1.795215 1.529581
H 2.793694 1.489276 -0.046210
H -1.189097 -0.877363 -2.024253
H -1.760194 0.485381 2.110771
H -1.011803 -2.151082 0.624652
H -1.230765 2.042789 -0.859346
H 1.481834 -0.296450 -1.792274
H 0.979915 1.465099 1.943759
H 4.195197 -0.067518 -1.459667
H 1.606007 2.462522 -0.001823
H 1.896317 -1.805232 0.718303
H -3.780447 1.510722 -1.190395

32
oh.10h2o, E= -840.479509
O 1.949697 1.491428 0.491985
O 2.902830 -0.917568 1.802050
O -3.095032 -1.578426 -1.114139
O -4.143567 -0.544460 1.283662
O 0.132472 0.055056 1.511973
O -2.203304 1.276481 0.828203
O -1.442014 0.775635 -1.839895
O 0.023602 2.833362 -0.794137
O 3.712999 0.233933 -1.065670
O -0.315809 -1.518732 -0.692272
O 2.404369 -2.234384 -0.657068
H 2.713582 -0.047855 1.400703
H 2.743472 -1.537351 1.060126
H 0.842234 0.703358 1.118362
H 0.661497 -0.504516 2.097202
H 0.805175 2.353231 -0.388728
H -0.616725 2.885079 -0.072920
H 3.046055 0.795095 -0.574415
H 4.302618 -0.063117 -0.363234
H 2.786679 -1.425821 -1.052960
H 1.442376 -2.155246 -0.792387
H -1.249102 -1.807837 -0.733589
H -0.208357 -1.013143 0.146805
H -2.925579 -0.748873 -1.584392
H -3.575942 -1.308966 -0.304867
H -3.505315 0.211268 1.200346
H -3.809017 -1.067318 2.018899
H -1.418188 0.871169 1.256397
H -2.055396 1.104335 -0.127624
H -0.849809 0.020728 -1.650370
H -0.873238 1.572288 -1.728635
H 2.333025 2.184886 1.037750

35
oh.11h2o, E= -916.947309
O -2.834026 1.944846 -0.076475
O -2.750858 0.108108 1.798389
O -0.001375 2.019153 -0.208124
O 0.076955 -0.191728 -1.696991
O 0.114889 -1.728844 0.233825
O -0.032100 0.158236 1.959533
O -3.017560 -1.984942 -0.041259
O -2.581680 -0.169558 -1.977190
O 2.915271 -0.024522 -1.950784
O 2.795043 1.829137 -0.047329
O 2.802714 0.043556 2.009745
O 2.746149 -2.030840 0.174653
H 0.023410 1.234662 -0.824947
H -0.937012 2.279710 -0.174275
H -1.789424 0.106387 2.040747
H -2.881457 -0.719790 1.291383
H 0.018806 -0.654260 1.328799
H -0.636741 -2.330556 0.236361
H -2.751173 0.654836 -1.486352
H -1.598712 -0.190901 -2.086095
H -2.845511 1.278801 0.681350
H -3.535579 2.576880 0.100850
H 0.038334 0.911539 1.341358
H 0.089038 -0.914127 -0.888149
H -2.841919 -1.371655 -0.807216
H -3.854917 -2.412310 -0.241719
H 1.750180 -2.095011 0.209910
H 2.914318 -1.515289 -0.630562
H 2.885984 -0.764820 1.449759
H 2.866866 1.264132 0.765697
H 1.874339 2.147895 -0.048759
H 2.895197 0.707789 -1.267264
H 0.951726 -0.221478 -2.114093
H 1.852375 0.064824 2.240520
H 3.644490 0.181922 -2.541254

35
oh.11h2o, E= -916.947134
O -1.338546 -2.310403 -0.150732
O -3.793318 -1.151619 -1.005321
O -2.188526 0.892565 -0.271640
O -0.019462 -0.249424 -1.289575
O 1.836854 1.845709 -1.350506
O 3.610802 0.130392 -0.018133
O 2.238724 -1.913151 -1.233866
O 1.091568 -2.995746 1.107259
O 0.949409 -0.065671 1.342691
O -1.631904 -0.559387 2.043001
O -1.500799 3.536616 0.090940
O 0.918924 2.816356 1.016471
H -0.836944 -1.596643 -0.634618
H -2.260117 -2.197601 -0.446528
H -3.843295 -1.083715 -1.963413
H -3.360761 -0.310838 -0.719551
H -1.343084 0.547212 -0.688794
H -2.086383 1.864819 -0.180515
H 1.435162 -1.354451 -1.336273
H 1.990044 -2.516935 -0.507606
H 1.171054 -2.076080 1.414805
H 0.183603 -3.041138 0.759240
H 0.069569 -0.190275 1.775714
H 0.736769 -0.085065 0.388764
H -0.639570 3.312569 0.538214
H -1.233766 3.938410 -0.741803
H -1.983828 0.094736 1.404077
H -1.612534 -1.367006 1.492764

H 1.149072 1.141622 -1.400015
H 2.622774 1.362319 -1.019185
H 3.276454 -0.673826 -0.481841
H 3.067864 0.161450 0.781545
H 1.329230 2.623811 0.137134
H 0.920469 1.933478 1.426154
H -0.141600 -0.296769 -2.242972

35
oh.11h2o, E= -916.946506
O -2.403629 -1.465591 -0.221921
O -3.900876 0.820334 -1.103148
O -1.543284 1.830355 -0.187629
O -0.246731 -0.197450 -1.276443
O 2.005211 1.316184 -1.315702
O 3.593946 -0.838013 -0.522574
O 2.425014 1.297037 1.557465
O 1.430654 -2.398501 -1.170401
O -0.394293 -3.315737 0.831462
O 0.467197 -0.681727 1.447171
O -2.044813 0.206031 2.027511
O 0.757445 3.374191 0.173548
H -1.597061 -1.056338 -0.635644
H -3.133027 -0.921752 -0.568364
H -3.880306 0.988471 -2.049927
H -3.156861 1.355066 -0.743142
H -1.023492 1.096180 -0.634109
H -0.890986 2.547142 -0.043784
H 0.822292 -1.634768 -1.298460
H 0.937780 -2.960915 -0.544301
H -0.048118 -2.527297 1.290825
H -1.242444 -3.007387 0.474587
H -0.371416 -0.332111 1.833919
H 0.346912 -0.559142 0.487842
H 1.265790 2.884087 -0.500562
H 1.127747 3.020323 0.997138
H -1.958399 0.958999 1.407023
H -2.364909 -0.503458 1.436152
H 1.177272 0.767177 -1.333497
H 2.725089 0.652564 -1.292169
H 2.895303 -1.515456 -0.687687
H 3.474478 -0.561819 0.395493
H 2.406033 1.375041 0.587609
H 1.764297 0.594266 1.724390
H -0.442692 -0.235759 -2.218033

35
oh.11h2o, E= -916.944841
O -3.746239 0.489659 0.498742
O -2.239828 2.572862 -0.374438
O 0.323216 1.888231 0.323174
O 3.038350 1.831124 -0.049192
O 3.365918 -0.142847 1.702198
O -2.495153 -1.067866 -1.410618
O -0.390019 0.729131 -2.150335
O 3.346062 -0.698373 -1.214094
O 0.854616 -1.730499 -1.750560
O -0.608590 -2.155188 0.266510
O -2.201059 -1.039117 2.253381
O 0.552359 -0.354040 1.837378
H -3.480005 -0.102731 -0.241415
H -3.310304 0.062281 1.265178
H 3.958560 0.045578 2.434721
H 3.293210 0.698049 1.167184
H 3.493580 -0.929845 -0.286373
H 2.487933 -1.115238 -1.449309
H 1.520423 -0.418423 1.919696
H 0.240737 -1.100277 1.248837
H 2.085547 2.056518 0.010238
H 3.102979 1.145317 -0.744669
H 0.263222 -1.946593 -0.913694
H -0.052515 1.147284 -1.332138
H 0.120617 -0.104180 -2.198232
H -2.052094 2.229357 -1.257452
H -2.843233 1.890143 0.020393
H -1.436882 -0.573268 2.620760
H -1.782162 -1.523476 1.503032
H -1.909362 -1.581207 -0.804936
H -1.896056 -0.379247 -1.756913
H -0.579513 2.275910 0.284134
H 0.296938 1.106026 0.926632
H -0.561846 -3.075302 0.542669
H 0.643152 -2.391287 -2.415507

35
oh.11h2o, E= -916.944432
O -3.832118 -0.152138 -0.767992
O -2.045777 1.860910 -1.286521
O -0.354031 1.568706 0.838908
O -1.534520 -1.700865 -0.992777
O 0.355085 0.296396 -1.803837
O -2.939167 0.177196 1.883354
O -0.482136 -1.092884 1.454553
O 1.834706 0.076782 2.372851
O 1.004890 -2.901781 -0.043633
O 2.557561 -1.199209 -1.611155
O 3.650104 0.497621 0.273696
O 1.897932 2.599571 -0.476253
H -3.163626 -0.844112 -0.963222
H -3.751336 -0.047252 0.200839
H 3.207649 1.324447 -0.000336
H 3.082471 -0.655288 -0.979062
H 2.151826 -1.904139 -1.061801
H 3.145076 0.234903 1.070455
H 1.057301 -0.436448 2.036355
H 1.147702 2.345114 0.100443
H 1.620117 2.282452 -1.345294
H 0.266253 -3.067136 -0.645403
H 0.603655 -2.253598 0.583500
H 0.341342 0.558576 -0.871288
H 1.188509 -0.248879 -1.882486
H -1.421776 1.450384 -1.902507
H -2.784150 1.210426 -1.194861
H -2.612545 1.082125 1.948150
H -2.114006 -0.345388 1.739307
H -1.258855 -1.553620 -0.053086
H -0.948313 -1.093647 -1.483208
H -1.067401 1.741061 0.177713
H -0.437758 0.628145 1.124060
H -0.644975 -1.626637 2.239659
H 1.492601 0.976067 2.432541

35
oh.11h2o, E= -916.944120

O -0.065053 0.123808 -1.214225
O -1.425713 -0.634516 2.086225
O 1.416439 -1.991024 -0.118056
O -0.994399 -3.062376 0.736565
O -1.873355 -1.515505 -1.445989
O -3.402903 -0.034828 0.085541
O -1.918032 2.071514 -1.134465
O 0.594475 1.151344 1.111209
O 2.613828 -0.367111 -2.337878
O -0.704467 3.607316 0.691904
O 3.270132 1.247377 -0.132527
O 2.782676 -0.631397 1.863436
H -0.679484 -0.030862 1.902703
H -2.146349 -0.313951 1.515764
H -1.199887 1.395475 -1.281843
H -2.631765 1.555861 -0.724024
H 2.460763 -1.193271 -1.857733
H 2.937350 0.244688 -1.641923
H 0.348865 0.778849 0.208859
H 0.180864 2.037179 1.150087
H 0.878327 -1.230928 -0.434507
H 0.730094 -2.602069 0.225531
H -1.029446 -0.814813 -1.424174
H -2.064064 -1.711913 -2.366816
H -1.122601 -2.307196 1.350225
H -1.376699 -2.727853 -0.096749
H 2.382903 1.522691 0.152978
H 3.455846 0.557975 0.537910
H 2.057855 -0.001500 2.003715
H 2.379865 -1.261465 1.225031
H -1.237105 3.118789 0.006549
H -0.102463 4.163013 0.187592
H -4.275496 -0.401294 0.251399
H -2.938167 -0.657966 -0.546483
H 0.641770 0.163745 -1.878011

35
oh.11h2o, E= -916.943411
O 1.207080 2.611285 -0.672457
H 0.829773 1.798824 -1.103560
H 0.457667 2.977645 -0.181741
O 1.940994 -0.529555 2.089201
H 1.046384 -0.173883 1.913042
H 2.532109 0.103732 1.608071
O 1.740236 -2.632090 0.495560
O 3.322038 1.142876 0.432491
O -1.341671 2.858095 0.829156
O -2.468101 1.163503 -1.340842
O -0.495829 0.344563 1.091824
O 0.116710 0.330989 -1.657824
O 2.651084 -0.759880 -1.475156
O -0.594494 -2.233965 -1.149134
O -3.172479 -1.453953 -0.628403
O -2.790595 -0.907150 1.926573
H -3.039142 -1.216154 1.006123
H -3.407082 -0.193730 2.118491
H -3.066416 -0.532973 -0.951444
H -2.305671 -1.878890 -0.831494
H -1.519319 0.912729 -1.495428
H -2.418388 1.872381 -0.684454
H -0.336835 0.165157 0.143007
H -1.271531 -0.193461 1.378749
H 0.097862 0.411320 -2.617026
H -0.294230 -1.330491 -1.428477
H 0.087041 -2.529919 -0.522007
H 1.751776 -0.362110 -1.578200
H 2.489411 -1.558426 -0.946417
H 2.089996 -3.422647 0.914411
H 1.845609 -1.884479 1.157383
H 2.648350 1.802662 0.161952
H 3.283575 0.487451 -0.299129
H -1.411173 3.321668 1.668262
H -1.009543 1.945845 1.041981

35
oh.11h2o, E= -916.942841
O 0.636242 -0.114287 1.032992
O 3.702162 -0.710748 -0.820707
O -1.426773 -1.583405 -0.313134
O 0.253118 -3.699217 -0.498636
O 2.018815 -2.157943 0.561875
O 2.927554 1.457941 0.696968
O -0.388366 1.871586 -0.547479
O -1.649933 0.312294 -2.505541
O -1.648895 -0.386268 2.580106
O 1.835984 3.531447 -0.574485
O -3.804266 -0.070814 -0.719839
O -2.660265 1.701953 1.115793
H 3.275904 -0.602407 -1.676431
H 3.094201 -1.336811 -0.304034
H 2.124648 0.995832 1.012983
H 3.362312 0.772178 0.139620
H -1.913297 -1.098928 1.983395
H -2.053105 0.419046 2.171463
H 0.053525 1.157551 -0.034709
H 0.295871 2.565720 -0.658670
H -0.741909 -0.998091 0.065185
H -0.957791 -2.448789 -0.416049
H 1.212415 -0.985014 0.924798
H 2.462730 -2.548275 1.320715
H 0.553348 -3.870604 -1.395839
H 1.002104 -3.152038 -0.062743
H -1.896101 1.962428 0.565010
H -3.222814 1.183237 0.501991
H -3.343941 0.255555 -1.516624
H -3.204771 -0.788455 -0.448580
H 2.347123 2.790090 -0.145219
H 1.790684 4.220017 0.095570
H -1.177027 0.988114 -1.978479
H -1.465907 -0.505033 -2.011427
H 0.038788 -0.195625 1.804764

35
oh.11h2o, E= -916.942217
O 0.170713 -0.719639 -1.840298
O 0.118234 -2.746001 -0.179681
O -0.272506 1.082651 -0.048967
O 1.385374 3.283601 -0.289675
O 2.864611 -0.617883 -1.244941
O 3.386645 1.607268 0.279136
O 1.612190 0.299592 1.981868
O 2.361247 -2.257918 1.041879
O -2.538364 -0.865416 -1.604455
O -2.949103 1.753893 -0.635402

O -4.238856 0.333824 1.552254
O -1.904778 -1.027577 1.004625
H 0.149986 -2.090667 -0.941957
H -0.604935 -2.410557 0.377531
H 2.705288 -3.031381 1.495508
H 1.494896 -2.531700 0.601059
H -2.005292 1.660156 -0.388380
H -3.441012 1.476785 0.157420
H -1.568460 -0.841655 -1.824439
H -2.800200 0.073932 -1.530905
H -0.056600 0.476743 -0.836871
H 0.247458 1.909265 -0.168451
H 1.900721 -0.629908 -1.476116
H 2.939094 -1.268208 -0.527441
H 1.316508 3.849941 0.484824
H 2.211588 2.743100 -0.143234
H -4.845366 -0.178821 1.007707
H -3.414263 -0.204709 1.556939
H -2.220401 -1.076461 0.061153
H -1.273621 -0.286980 0.971690
H 2.923368 1.206753 1.045980
H 3.310722 0.888307 -0.392529
H 1.874969 -0.620638 1.791321
H 0.905178 0.500891 1.337972
H 0.287110 -0.625351 -2.789533

35
oh.11h2o, E= -916.942106
O -1.215712 -1.551440 1.710194
O -3.382682 -1.009011 0.367813
O 0.511105 -2.048083 -0.531653
O 0.935435 0.090937 2.424850
O 2.676897 -1.549182 1.125406
O 4.237011 -0.083077 -0.677184
O 2.117117 1.413759 -1.218994
O 0.862414 2.363581 1.128030
O -0.086352 0.096236 -2.047335
O -1.197534 2.190736 -0.853677
O -3.397549 1.736882 0.544687
O -2.113104 -1.633627 -1.961833
H -1.444718 -0.889014 -2.043927
H -1.541849 -2.362959 -1.687892
H -3.051603 -1.220217 -0.539936
H -3.510577 -0.032935 0.388864
H -2.083156 -1.319733 1.245281
H -1.387717 -2.334241 2.240619
H 2.001606 -1.827932 0.465922
H 3.333456 -1.049383 0.598076
H 1.652664 -0.470068 2.031617
H 0.117509 -0.416739 2.257826
H 0.404108 -1.261414 -1.134467
H -0.109853 -1.886985 0.202286
H 3.462411 0.510965 -0.899108
H 4.318988 -0.668246 -1.436268
H 0.880962 1.498005 1.643490
H 1.206332 3.042407 1.714096
H 1.323631 0.896602 -1.535942
H 1.801313 1.864342 -0.415640
H 0.063640 0.161198 -2.995743
H -0.832740 1.415543 -1.376135
H -0.506992 2.392573 -0.200147
H -2.604862 1.924503 -0.041169
H -3.068927 1.892030 1.435869

35
oh.11h2o, E= -916.939750
O 1.311786 -2.639536 0.110151
O -1.284836 -2.458813 -1.033930
O -2.929485 -1.160781 0.876990
O 2.686320 -0.732903 -1.162664
O -1.036622 -0.023808 2.596045
O 1.262320 -1.032695 2.256501
O 2.933183 0.731126 1.014905
O 1.106973 2.759321 0.011874
O -1.259939 2.260509 1.107735
O 1.199434 0.928402 -2.204996
O -1.068017 -0.013699 -2.485708
O -2.817909 1.080571 -0.895258
H 0.424358 -2.666599 -0.300051
H 3.467207 -0.978382 -1.665477
H 1.847116 -2.001522 -0.441664
H -1.197455 -0.150418 -3.427825
H -0.080526 0.398111 -2.357021
H -1.848137 1.930859 0.400299
H -1.165624 1.500766 1.726851
H -1.154086 -1.663191 -1.582404
H -1.882568 -2.156228 -0.321110
H 0.222194 2.577712 0.438340
H 1.259963 3.703633 0.099904
H -2.283874 -0.844184 1.541174
H -3.073448 -0.392308 0.299302
H 2.963628 0.162878 0.184330
H 2.393656 1.493897 0.750946
H 1.250142 -1.720142 1.531722
H 1.904960 -0.356014 1.922639
H -1.193861 -0.057870 3.543486
H -0.098530 -0.438804 2.443332
H -2.176278 0.690054 -1.565184
H -3.494945 1.547737 -1.391221
H 1.883836 0.178238 -1.798846
H 1.206610 1.660230 -1.564691

35
oh.11h2o, E= -916.939357
O -1.974996 -0.303432 -1.633269
O -0.864616 -2.808729 -0.946363
O 1.181502 -2.426065 1.118167
O 3.518393 -1.708590 0.055355
O 0.477045 -0.030594 -1.423882
O 3.061592 0.761553 -0.733124
O 2.013032 2.439494 1.144952
O -2.300707 2.091271 -0.338561
O -3.318142 -1.432386 0.391065
O 0.206183 2.950318 -0.891492
O 0.313122 0.201081 1.378181
O -2.396214 0.630059 2.069373
H -1.287666 -1.968471 -1.229231
H -0.265383 -2.589365 -0.215726
H -0.573632 -0.055088 -1.599751
H 0.725906 -0.953931 -1.553076
H -2.278717 1.257502 -0.868561
H -2.429598 1.769951 0.577671
H -2.956491 -0.952564 -0.401843
H -2.810884 -2.253592 0.376907

H -2.801619 -0.141743 1.623451
H -1.436917 0.484650 1.962116
H 0.600674 -0.711513 1.560423
H 0.295891 0.225959 0.396430
H 1.298088 -3.147205 1.742403
H 2.074796 -2.230741 0.727022
H 3.411035 -0.736625 -0.181223
H 3.812552 -2.134787 -0.754898
H 2.176838 0.633761 -1.128085
H 2.884985 1.417636 -0.014070
H 1.486080 1.716204 1.532639
H 1.382706 2.825645 0.495251
H -0.730466 2.744461 -0.631490
H 0.457893 2.188265 -1.428182
H -2.365623 -0.300240 -2.511379

35
oh.11h2o, E= -916.938955
O 0.294695 -2.504854 0.399167
O 2.676966 -1.807382 -0.445310
O -0.959603 -1.116144 2.343920
O -2.940737 -1.086246 0.886923
O -2.912400 1.523598 0.611431
O -2.102841 1.499810 -1.864831
O -1.620762 -1.313223 -1.370558
O 1.038300 -0.839055 -2.484613
O 0.588795 1.566957 -1.228157
O 2.360475 2.556377 0.768971
O 4.202723 0.336861 0.871902
O -0.220821 1.393676 1.460685
H 1.821010 -2.092329 -0.009749
H -0.314120 -2.155420 -0.279939
H -0.364805 0.499928 1.842606
H 0.056599 1.257042 0.531512
H -2.490922 1.570627 -0.932279
H -2.720811 1.922948 -2.465951
H -0.043284 -2.105005 1.232953
H -1.136978 -1.271435 3.274506
H -2.053659 1.681303 1.055065
H -3.070979 0.542290 0.762222
H 3.245392 -2.582374 -0.462664
H -3.705863 -1.636106 1.070156
H -1.889477 -1.152357 1.794452
H 0.200259 -1.227985 -2.181228
H 0.855100 0.752442 -1.713602
H -0.302503 1.768036 -1.571026
H -2.198343 -1.321654 -0.543314
H -1.747545 -0.410938 -1.712425
H 3.652437 -0.335640 0.441237
H 1.673805 2.299860 1.404480
H 3.657196 1.144614 0.860987
H 1.910023 2.399558 -0.079731
H 1.729157 -1.230415 -1.914526

35
oh.11h2o, E= -916.937089
O 0.115144 -2.745635 -0.972573
O 2.600208 -1.735760 -0.797922
O -1.749139 -2.438576 1.077578
O -3.199239 -0.876097 -0.265936
O -2.972653 1.299300 0.996685
O -1.459007 2.644480 -0.998398
O -0.904204 -0.158790 -1.298541
O 1.690295 0.542932 -2.237565
O 1.381831 2.792477 -0.529301
O 1.874892 1.520490 1.918254
O 3.763646 -0.577177 1.656757
O -0.537460 0.053971 1.462554
H 1.725153 -2.201907 -0.763871
H -0.251924 -1.910850 -1.329641
H -0.835065 -0.871199 1.560635
H -1.352823 0.592314 1.517404
H -2.030933 2.422176 -0.241852
H -1.301082 1.772168 -1.398916
H -0.432656 -2.881530 -0.174861
H -2.227526 -2.972043 1.716640
H -3.687212 1.484064 1.610677
H -3.212233 0.439669 0.475116
H 3.202175 -2.328582 -1.256561
H -3.942237 -1.136226 -0.814326
H -2.443924 -1.896456 0.527589
H 0.751945 0.296641 -2.203716
H 1.602588 2.082288 -1.164471
H 0.416071 2.901763 -0.612179
H -1.849322 -0.439247 -1.088687
H -0.547333 0.009434 -0.398864
H 3.367554 -0.988136 0.874482
H 1.083036 0.957314 1.909536
H 3.209352 0.215401 1.805202
H 1.786544 2.047853 1.095478
H 2.135344 -0.167830 -1.737541

35
oh.11h2o, E= -916.937069
O 1.837219 1.710809 -0.657092
O 3.684380 0.074284 -1.222903
O -0.412350 0.584606 -1.237086
O -3.350808 -2.120178 -0.293822
O -1.070795 -2.049370 -1.716063
O -0.483623 -0.321973 1.357455
O 0.397512 -2.819016 0.516229
O 3.040694 -1.793217 0.514908
O 2.046485 0.642516 1.817337
O -0.190356 3.642536 0.291683
O -3.178864 0.220999 1.180741
O -2.589265 2.194343 -0.492310
H 0.472451 1.074844 -1.130068
H -1.131585 1.239612 -1.207678
H 0.336881 0.076071 1.732625
H -0.470288 -0.018890 0.416795
H 2.032741 1.178806 0.982374
H 2.479474 -0.188014 1.563117
H 0.443751 2.912162 0.197384
H -1.052745 3.246171 0.092021
H -3.364530 2.497718 -0.971104
H -2.897097 1.489262 0.148474
H -3.343226 -0.590263 0.648510
H -2.272094 0.080578 1.525923
H -3.124507 -2.810813 0.337582
H -2.557454 -2.081578 -0.898092
H -0.471629 -2.450326 -1.048584
H -0.802667 -1.105638 -1.724749
H 0.025427 -2.033164 0.971995
H 1.343704 -2.596203 0.440415

H 3.804144 -2.352468 0.681161
H 3.304665 -1.156415 -0.215916
H 2.907592 0.756956 -1.059550
H 4.467293 0.533692 -0.903701

H 2.027873 2.567186 -1.051549
